# A hyperlocal hybrid data fusion near-road PM_2.5_ and NO_2_ annual risk and environmental justice assessment across the United States

**DOI:** 10.1371/journal.pone.0286406

**Published:** 2023-06-01

**Authors:** Alejandro Valencia, Marc Serre, Saravanan Arunachalam

**Affiliations:** 1 Department of Environmental Sciences and Engineering, The University of North Carolina at Chapel Hill, Chapel Hill, North Carolina, United States of America; 2 Institute for the Environment, The University of North Carolina at Chapel Hill, Chapel Hill, North Carolina, United States of America; Trent University, CANADA

## Abstract

Exposure to traffic-related air pollutants (TRAPs) has been associated with numerous adverse health effects. TRAP concentrations are highest meters away from major roads, and disproportionately affect minority (i.e., non-white) populations often considered the most vulnerable to TRAP exposure. To demonstrate an improved assessment of on-road emissions and to quantify exposure inequity in this population, we develop and apply a hybrid data fusion approach that utilizes the combined strength of air quality observations and regional/local scale models to estimate air pollution exposures at census block resolution for the entire U.S. We use the regional photochemical grid model CMAQ (Community Multiscale Air Quality) to predict the spatiotemporal impacts at local/regional scales, and the local scale dispersion model, R-LINE (Research LINE source) to estimate concentrations that capture the sharp TRAP gradients from roads. We further apply the Regionalized Air quality Model Performance (RAMP) Hybrid data fusion technique to consider the model’s nonhomogeneous, nonlinear performance to not only improve exposure estimates, but also achieve significant model performance improvement. With a R^2^ of 0.51 for PM_2.5_ and 0.81 for NO_2_, the RAMP hybrid method improved R^2^ by ~0.2 for both pollutants (an increase of up to ~70% for PM_2.5_ and ~31% NO_2_). Using the RAMP Hybrid method, we estimate 264,516 [95% confidence interval [CI], 223,506–307,577] premature deaths attributable to PM_2.5_ from all sources, a ~1% overall decrease in CMAQ-estimated premature mortality compared to RAMP Hybrid, despite increases and decreases in some locations. For NO_2_, RAMP Hybrid estimates 138,550 [69,275–207,826] premature deaths, a ~19% increase (22,576 [11,288 – 33,864]) compared to CMAQ. Finally, using our RAMP hybrid method to estimate exposure inequity across the U.S., we estimate that Minorities within 100 m from major roads are exposed to up to 15% more PM_2.5_ and up to 35% more NO_2_ than their White counterparts.

## Introduction

Traffic related emissions are a significant source of urban air pollution [1]. Exposure to traffic related air pollutants (TRAPs) such as PM_2.5_ and NO_2_ has been associated with a myriad of adverse health effects [2–8]. Additionally, concentrations from TRAPs are the highest in the vicinity of heavily trafficked roads [9, 10]. In the U.S., around 19% of the population resides near heavily trafficked roads and in states with higher population e.g., California, up to 40% of the population live close to TRAPs [11]. These estimates not only reinforce how widespread the exposure to TRAPs is in the U.S., but it also shows that the risk is highly variable and potentially greater in areas with higher population and increased road densities. In addition, many studies have shown that a disproportionate amount of the health risk is placed on Minority populations [12–22], a group which is already found to be more susceptible to air pollution exposure.

Assessing the burden of disease from TRAPs usually involves air quality and health modeling. Typically, these nationwide modeling efforts have been conducted with chemical transport models (CTMs) at coarse (12 km x 12 km or even 36 km x 36 km) spatial resolutions [23–31]. Dedoussi et al. [31] estimated 30,800 annual premature deaths in 2011 across the U.S. due to road transportation attributable to O_3_ and PM_2.5_ using 55-km x 55-km grid resolution using the GEOS-Chem CTM but did not consider secondary organic aerosols in their estimate. Davidson et al. [30] used a 12 km x 12 km (12-km) resolution model and estimated 9,666 annual premature deaths across the U.S. that were attributable to on-road PM_2.5_. This corresponded to 9.6% of the total PM_2.5_ attributable deaths for 2011. Additionally, Davidson et al. (2020) project that in 2025 the on-road premature deaths attributable to PM_2.5_ across the U.S. will correspond to 5.6% of the total premature deaths. These estimates at coarse resolutions, however, lack the spatial gradients necessary to assess the relationship between population exposure to TRAPs and health impacts. A few studies have explored the effect of grid resolution on estimates of air pollution from CTMs on premature mortality [27, 32–34]. But even at 1 km x 1 km (1-km) resolution, these types of studies might not fully capture the fine-scale TRAP concentration gradients. This can be attributed to the fact that most TRAP concentrations are highest near roads and decrease to background levels within 150 m to 200 m from the road [10, 35, 36]. Thus, capturing variability within a grid cell at fine spatial scales is critical for risk assessment. In risk assessment, higher population density is usually positively associated with air pollution, thus a method that does not capture within-cell variability will suffer from exposure misclassification. Therefore, diverse methods have been developed to address the uncertainty due to the dramatic spatial variation of TRAPs at even smaller spatial resolutions. Some studies have developed statistical regression-based models that estimate TRAPs at a spatial resolution of < 1 km [37–44]. These models, however, use surrogate indicators of emissions that are specific to an area and ignore the physical and chemical processes (e.g., dispersion, advection/diffusion, chemical reactions in gaseous and aerosol phases, and deposition) that can better quantify road source contribution.

To overcome these challenges, hybrid approaches have been developed that take coarse scale predictions of TRAPs from CTMs and combines them with high resolution dispersion model estimates [45–51]. As opposed to CTMs which allocate emission to a grid, dispersion modeling can represent emissions in a much finer spatial resolution that retains the physical characteristics and shape of the emitted plume. Because most dispersion models lack the ability to predict secondary pollutants such as secondary PM_2.5,_ combining these models avoids underestimating the total impact of a pollutant. Thus, by combining results from CTMs with dispersion models, predictions will include regional background, detailed chemical mechanisms, and emission sources that might not be included in a dispersion model. Using a hybrid air quality model, Parvez and Wagstrom [51] studied the effect of resolution (census block group vs 12-km grid resolution) on health impacts attributable to PM_2.5_ in three locations in Connecticut. Their findings showed an underestimation of health impacts at the coarser resolution. Their study, however, upscaled hybrid PM_2.5_ concentrations from 0.04 km x 0.04 km grid resolution to census block groups (losing the fine-scale variability necessary to capture elevated air pollution from roads). The study by Chang et al. [47], which estimated TRAP concentrations using a hybrid model at census block level, concluded that their hybrid approach estimated 24% more on-road PM_2.5_-related premature mortality than a CTM at a 36 km x 36 km resolution in central North Carolina. However, neither of these studies considered the biases in either model (i.e., dispersion model or hybrid model), and results from the modeling were localized to a small region. Recently, Shukla et al. [50] developed a hybrid modeling approach that combines C-TOOLS [52], a local-scale dispersion model for primary PM_2.5_ with the CoBenefits Risk Assessment (COBRA) Screening model [53] with secondary PM_2.5_ components. This model was used to characterize ZIP-code level air pollution estimates of PM_2.5_ in New York City for performing rapid assessment for evaluating health benefits of emissions reduction measures.

Continuing these efforts, our work describes and applies a novel hybrid data fusion method that combines model predictions from a dispersion model specially designed to predict on-road emission and a CTM at 12-km resolution with observations to produce fine-scale air quality characterization of PM_2.5_ and NO_2_ at census block levels across the continental U.S. In this approach, we also consider the nonhomogeneous, nonlinear behavior of model performance and thus have the ability to correct biases at fine-scale. Additionally, by estimating air pollution at high resolution our model will be able to assess whether our method can capture the variability necessary to better characterize the spatial gradient from TRAPs to provide more accurate assessments of the health risk overall. This approach will aid in further identifying vulnerable populations, quantify their exposure inequity near and away from roads, and prevent potential exposure misclassification.

## Methods

### Hybrid data fusion overview for 2016

The hybrid data fusion approach used in this study combines the gaussian Research LINE source dispersion model (R-LINE) [54] developed for near-roadway assessments, with the Community Multiscale Air Quality Model (CMAQ) CTM [55] version 5.2.1 [56] with Carbon Bond 6 version r3 at 12-km horizontal grid resolution with aero6 treatment of secondary organic aerosols (SOA) set up for standard cloud chemistry for the year 2016. The data fusion approach then applies the Regionalized Air quality Model Performance (RAMP) method that uses observations from the Air Quality System (AQS) network to correct biases in the model [57]. This hybrid data fusion approach models total hourly average PM_2.5_ and NO_2_ concentration that consider the fine-scale resolution of on-road emissions for 2016 for each of the 11,063,509 Census blocks for a full year from January 2016 to December 2016 across the continental U.S.

### Measurement data processing

Hourly, daily, and annually observed NO_2_ and PM_2.5_ from EPA’s AQS database [58] for each space/time location during 2016 were collected from monitoring stations.

### Emissions and meteorological data processing

Emissions for CMAQ and R-LINE are based on the 2016v7.2 (beta) platform [59]. This platform was produced by the National Emissions Inventory Collaborative and contains the emission inventories for 2016. For R-LINE, specifically, this platform provided emissions factors, fleet mix, and temporal allocation tables. These datasets were combined with road network data like geometry and annual average daily traffic (AADT) for major (or primary) road types, including interstates, principal arteries, minor arteries, and major collectors from the United States Federal Highway Administration (FHWA)’s Highway performance monitoring system (HPMS). The data set consists of ~7.5 million major road segments also from 2016 and was used to calculate hourly emissions using an approach similar to previous studies [60–62]

The gridded meteorological data for 2016 were obtained from version 3.8 of the Weather Research and Forecasting Model (WRF) [63]. For CMAQ, WRF meteorological outputs were processed using the Meteorology-Chemistry Interface Processor (MCIP) package [64], version 4.3 for the continental U.S. at a 12-km resolution. The meteorological inputs for R-LINE were also based on the same 12-km resolution WRF data used for CMAQ. Given that our hybrid data fusion approach will rely on R-LINE estimates for all major roadway sources within every CMAQ grid cell, R-LINE ready meteorological files were created using the Mesoscale Model Interface Program (MMIF) [65] for every grid cell. MMIF outputs have been shown to compare well against observed meteorological data and have been applied to dispersion models to address sparse observations networks [49, 66, 67].

### Hybrid model

To combine CMAQ and R-LINE predictions we follow approaches similar to previous studies [45, 48, 49, 68] as discussed below. Thus, to create PM_2.5_ estimates, we first remove primary roadway contributions from CMAQ to avoid double counting (This is also done for NO_2_ estimates). It is of note, that R-LINE can only estimate primary PM_2.5_ and thus we rely on CMAQ to provide secondary PM_2.5_. Additionally, the effect of resolution shows greater biases for primary PM_2.5_ species than secondary species [27]. To begin, we run R-LINE for all primary roads (i.e., Interstates, principal/minor arteries, and major collectors) in each CMAQ 12-km grid cell to estimate concentrations at a spatially distributed 1-km grid of 144 receptors. Then, we average this R-LINE grid of 1-km grid resolution within every CMAQ 12-km grid cell to obtain the R-LINE_GAVG_. We then subtract this R-LINE_GAVG_ from the total CMAQ estimate (CMAQ_TOT_) that includes impacts from all sources in each grid cell to remove on-road contributions. Note that CMAQ_TOT_ represents an average value for the entire grid cell. This computed difference then corresponds to an estimate of CMAQ without primary roadway attributable PM_2.5_ at a 12-km resolution. The next step is to linearly interpolate this difference to Census Block Centroids (CBCs). These interpolated values are then added to the primary PM_2.5_ estimates from R-LINE at a CBC resolution (R-LINE_CBC_). Because we are combining models with different biases, there is the potential of negative estimates of CMAQ without primary roadways. If this is the case, we rely only on R-LINE_CBC_ for the hybrid estimate (i.e., we zero-out CMAQ without primary roads). The resulting concentration represents the hybrid PM_2.5_ at the CBCs as shown in Eq 1.


$${HYB_{\left(PM_{2.5},NO_{2}\right)}}_{}={\left[(CMAQ_{TOT}-R‐LINE_{GAVG}\right)}_{interpolate}]+R‐LINE_{CBC}$$ (1)

The hybrid estimate refines the variability of the coarse CMAQ estimate to capture variability within each CMAQ grid described by R-LINE (i.e., hyperlocal variability of primary PM_2.5_ from mobile emissions occurring within each CMAQ grid). However, note that the hybrid estimate does not refine the variability of the coarse CMAQ estimate arising from the variability of non-mobile emissions within each CMAQ grid. A similar hybrid approach is used for NO_2_, a pollutant that has been linked to increased mortality in several epidemiological studies[69]. However, in this approach we model NO_x_ using R-LINE and then use the polynomial method described by Valencia et al. [62] that transforms NO_x_ to NO_2_. This is an empirical approach based upon fitting a 4^th^ order polynomial to existing near-road observations across the continental U.S. Using this polynomial, we can calculate a yield of NO_2_ to NO_x_ with which we are able to obtain NO_2_ estimates.

### Regionalize Air quality Model Performance (RAMP) bias correction

Once we have combined CMAQ with R-LINE to create the “Hybrid” estimate, we apply the RAMP method [57]. This method tries to account for the space/time variability in model performance which has been shown to fluctuate significantly across the national domain in other studies [57, 70, 71]. In this study, the main goal of RAMP is to correct the hybrid model biases using measurements. These biases have been shown to be nonhomogeneous and nonlinear [57, 70, 71], and which RAMP can account for.

To achieve this, the first step in the RAMP method consists of selecting the most relevant observation and prediction pairs $\left(z_{k},{\tilde{z}}_{k}\right)$ in time and space at each CMAQ 12-km grid cell *G(****p****)* with the space/time coordinate *p*. In this study, we chose to pair daily averaged model/observed values for the entire year. For PM_2.5_, we obtained the 50 closest monitoring sites while for NO_2_, we obtained the closest 10 sites. The number of stations was chosen to be as spatially specific to correlate with spatial trend while still ensuring a consistent pattern not highly influenced by outliers. Additionally, more sites were necessary for PM_2.5_ given that most of the daily measurements are reported every 3 days as opposed to NO_2_ sites where most sites provide daily measurements throughout the year.

**Fig 1:**
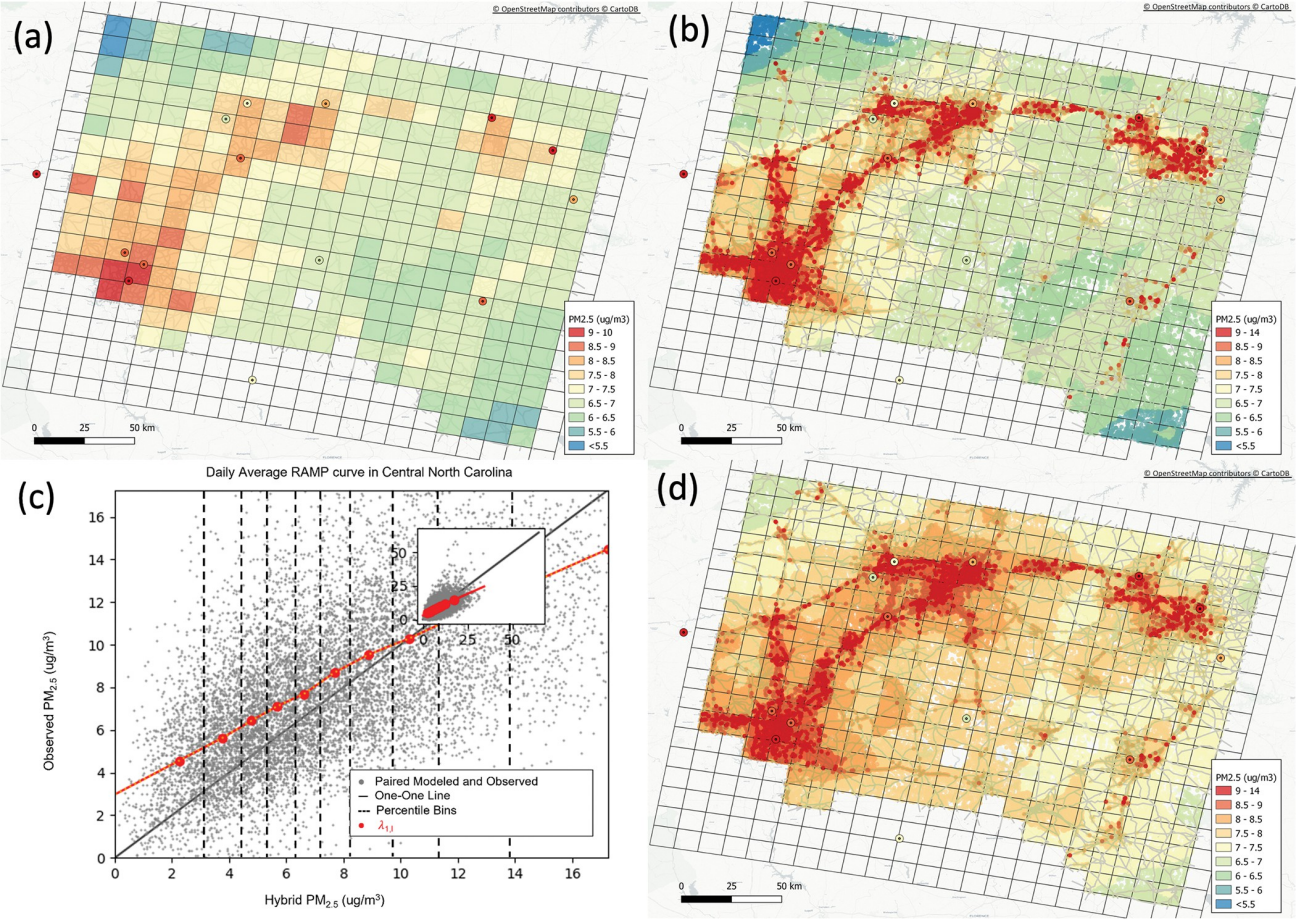
Spatial map of 2016 annual PM_2.5_ model concentrations for (a) CMAQ 12 km x 12 km grid resolution for a subdomain in central North Carolina (b) CMAQ + R-LINE Hybrid, and (d) RAMP Hybrid at census block centroids. Each map includes measurements represented as circles. The gray lines represent the major roads. The gradient bar in figures a, b, and d represents concentration levels in μg/m^3^. Also shown is one of the RAMP curves (c) used to correct biases from the Hybrid model. The grey dots represent paired observed and modeled daily PM_2.5_ concentrations for all of 2016 consisting of the 50 closest AQS measurement stations to the centroid of the CMAQ grid cell *G(p)*. The dashed vertical lines represent the 10 equally divided bins used to stratify all the paired data where each bin includes one decile of all the paired points. The solid black line is the one-to-one line between model and observed. The red dots in each bin identify $\lambda {}_{l}({\tilde{x}}_{l},G(p))$ the average of paired observed values within each decile bin. The red dots are linearly interpolated to obtain *λ*_*1*_*(p)* corresponding to the hybrid modeled data $\tilde{x}(p)$ with *G(p)*.

The second step is to stratify the pairs in 10 equal percentile bins of increasing predicted values ${\tilde{z}}_{k}$, and for each bin ${\tilde{z}}_{l}$ we calculate the mean $\lambda_{1}({\tilde{z}}_{l},G(p))$ of the observed values (see detailed equations in Reyes et al. [57] and Xu et al. [71]). The third step is to create interpolation/extrapolation lines that connect the mean of each bin $\lambda_{1}({\tilde{z}}_{l},G(p))$. This piecewise linear function is then used to obtain bias corrected Hybrid (RAMP Hybrid) value $\lambda_{1}({\tilde{z}}_{k},G(p))$ for the pertinent Hybrid value. Fig 1C shows this RAMP function and how the mean at each bin changes as a function of the average modeled value bin as well as how the method accounts for the nonlinear performance of the Hybrid model.

In addition to the steps laid out by Reyes et al. [57], we improve on the RAMP method by safeguarding that the slope between each $\lambda_{1}({\tilde{z}}_{l},G(p))$ is never negative. In other words, we make sure that the slope is always monotonically increasing [72]. To do so, we first calculate the mean of all observed values *z*_*K*_ pertinent to the specific *G(****p****)* as λ_1,M_. We then compare λ_1,M_ with $\lambda_{1}({\tilde{z}}_{5},G(p))$ the λ_1_ in the 5^th^ decile bin. If λ_1,M_ < $\lambda_{1}({\tilde{z}}_{5},G(p))$, $\lambda_{1}({\tilde{z}}_{5},G(p))$ is set to λ_1,M_. In a similar manner, we compare the 5^th^ and the 4^th^ bin making sure that $\lambda_{1}({\tilde{z}}_{k},G(p))$ < $\lambda_{1}({\tilde{z}}_{k-1},G(p))$ and set them equal if necessary. The same algorithm is applied to the other half of the RAMP curve (k = 6 to k = 10). In this case, we compare λ_1,M_ to 6^th^ bin and set $\lambda_{1}({\tilde{z}}_{6},G(p))$ to λ_1,M_ if λ_1,M_ > $\lambda_{1}({\tilde{z}}_{6},G(p))$. This improvement safeguards the ordinality of estimates of the original model with the same *G(****p****)*.

### Health impact evaluation

To evaluate the health impact of air pollution from the model, we combine population data, background health data, and concentration response functions (CRFs) from epidemiological literature that provide the relationship between PM_2.5_ and NO_2_ to health impacts. For estimating premature mortalities attributable to PM_2.5_, we apply a CRF from a published meta-analysis by Vodonos et al. [73]. In this study, findings show that there was a 1.29% (95% CI 1.09–1.5) increase in all-cause mortality for every 10 μg/m^3^ increase of PM_2.5_. For NO_2_-attributable premature mortalities, we use a CRF associating all-cause mortality to long-term effect of NO_2_ exposure with a hazard ratio of 1.04 (95%CI 1.02–1.06) per 10 μg/m^3^ increase in NO_2_ [74]. This approach is similar to what was used in a recent study that focused on NO_2_-related premature mortalities from commercial aviation [75].

Population data at census block level were obtained from the U.S. Census Bureau for 2010 and projected to 2016. The data set also included population by race/ethnicity at census block level [76]. The data was split by age with data from the U.S. Census American Community Survey for the year 2016. Given that this dataset was at census tract resolution, we estimated the percentage of the population over 25 years old for each census tract and applied that fraction to each census block inside the census tract. Average county level baseline mortalities from 1999 to 2016 (the most recent available years) were obtained from the U.S. Centers for Disease Control Wide-ranging Online Database for Epidemiologic Research (CDC-WONDER) [77].

## Results

### Hybrid data fusion

Fig 1 shows the progression of the hybrid data fusion model for 2016 annual PM_2.5_ in the central North Carolina region as an illustration. Fig 1A shows PM_2.5_ concentrations at 460 12-km grid cells. Fig 1B shows the hybrid combination of CMAQ and R-LINE at 144,276 census block centroids. Results show how the hybrid method captures fine-scale gradients near major roads that CMAQ averages out as shown in Fig 1A. Specifically, hybrid captures elevated concentrations surrounding I-40 from Winston-Salem to Statesville in the northwest and it also captures hotspots from I-95 near Fayetteville in the southeast of the domain. Please refer to Supporting Information (S1 File) for a map of central North Carolina city locations. Fig 1C shows the shape of the RAMP curve used to correct biases from the Hybrid model. From this RAMP curve, we can see that the hybrid model is underpredicting up until ~10 μg/m^3^ after which it begins to overpredict concentrations. Thus, the RAMP adjustment will increase low hybrid concentrations and decrease high hybrid concentrations. This is an example of how the RAMP methods consider the nonlinear model performance at varying concentrations of the model. Fig 1D shows the final RAMP hybrid product where hybrid concentrations have been adjusted. The bias correction that stands out the most occurs away from the big metropolitan areas where concentrations were increased by ~1.5 to ~2 μg/m^3^ to better represent measurement surrounding the area. A harder to detect correction (that happens on the other side of the RAMP) occurs on the I-285 from Winston-Salem to Lexington where concentrations also increased by ~2 μg/m^3^.

**Fig 2:**
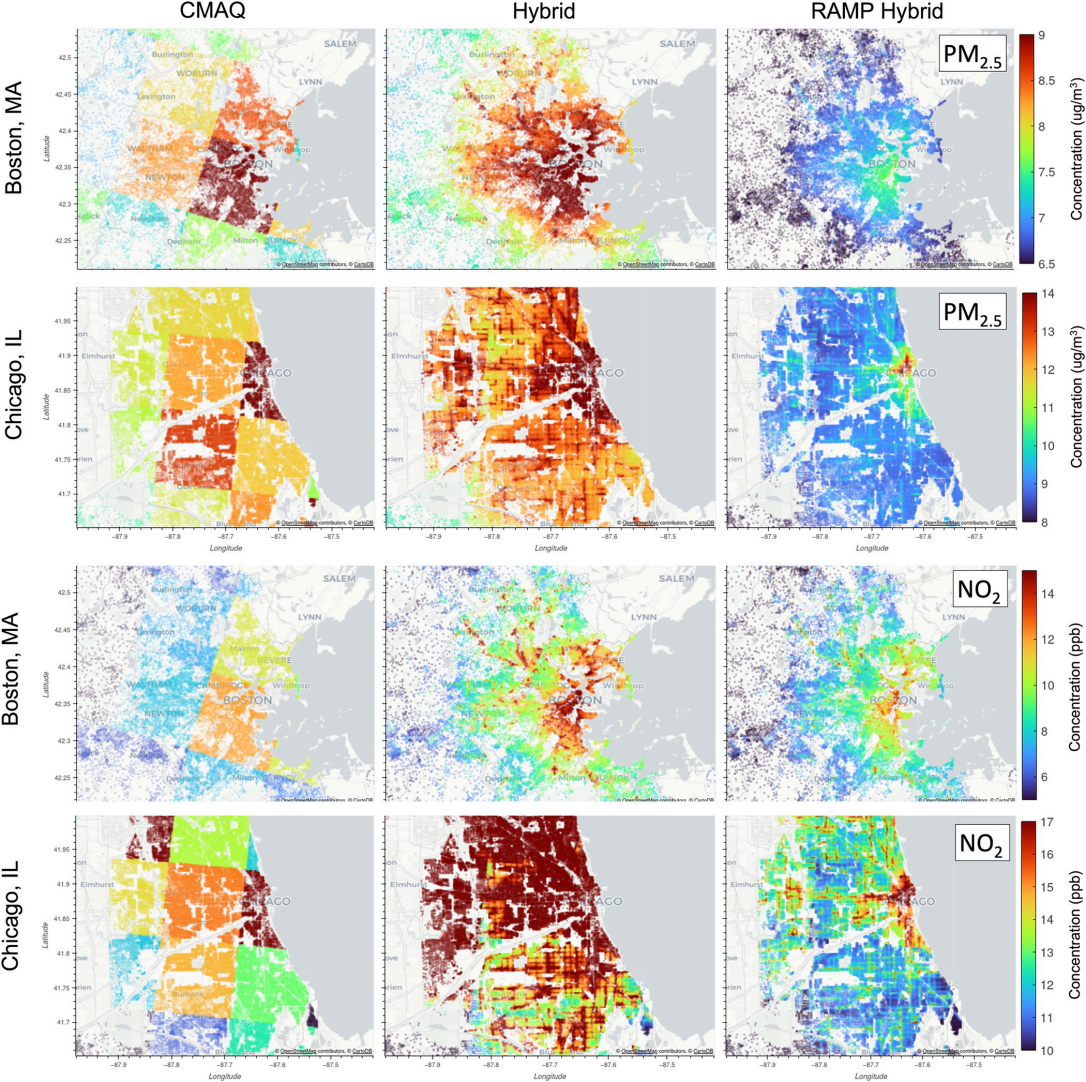
Spatial map of 2016 annual PM_2.5_ and NO_2_ model concentrations for CMAQ, CMAQ R-LINE Hybrid, and RAMP Hybrid at census block centroids at Boston, Massachusetts and Chicago, Illinois. The gradient bar represents concentration levels in μg/m^3^ that range from the smallest 10^th^ percentile of the 3 three models to the highest 90^th^ percentile of the three models for the corresponding domain.

The spatial maps from Fig 2 show annual PM_2.5_ for CMAQ, Hybrid, and RAMP Hybrid at census block level focusing on smaller domains in Boston, MA and Chicago, IL. These are also considered the most congested cities in the U.S. according to the Global Traffic Scorecard released by INRIX, a data analytics company that studies traffic globally [78]. These maps reinforce that by combining R-LINE and CMAQ the hybrid results can capture sub-grid scale (within each 12-km grid) variability, which most traditional grid-scale models cannot. For Boston, because we are attempting to capture concentrations for all models, the color scale does not highlight the near-road gradients in the domain. Thus, while concentrations of the 10^th^ and 90^th^ percentile for CMAQ and Hybrid range from 6.9 to 8.9 μg/m^3^, the RAMP hybrid concentrations range from 6.1 to 7.2 μg/m^3^. This difference in concentration range is due to the RAMP regional correction, where this area was found to be biased high across the board. Please refer to the S1 File for spatial maps of Boston that have a color scale that highlight the near-road gradients for the RAMP method. In Chicago, the spatial maps highlight the near-road concentration gradients where both Hybrid and RAMP Hybrid show better distribution of high and lows. Once again, CMAQ and Hybrid both show significantly higher concentrations than RAMP Hybrid. Concentrations of the 10^th^ and 90^th^ percentile for CMAQ and Hybrid range from 10.5 to 13.9 μg/m^3^, while the RAMP hybrid concentrations range from 8.4 to 9.6 μg/m^3^.

For annual NO_2_ shown in Fig 2, Hybrid and RAMP are capturing higher concentrations near roads in Boston. One can see the impact from the Concord Turnpike (US-2) and US-3 coming from the north into downtown Boston from both Hybrid and RAMP hybrid. Additionally, these models show peaks >14 ppb near downtown Boston not captured by CMAQ. In Chicago, the evolution from CMAQ to Hybrid to RAMP Hybrid is drastic. Both CMAQ and Hybrid have higher overall concentrations surrounding downtown. Hybrid estimates significantly high values that we truncated in this area where the 90^th^ percentile concentrations for Hybrid were ~26 ppb. Thus, the range of the color scale only shows the 10^th^ and 90^th^ percentile of CMAQ and RAMP Hybrid. These high values only seen in Hybrid emphasize the need for the RAMP bias correction. The concentration adjustments due to the RAMP method in both these areas also showcase the ability of this method to correct regional and nonlinear biases, a capability that is lacking from other linear regression methods. Additionally, this method keeps the steep near-road gradients (as seen the most in the Chicago area) and captures the within-cell spatial variability of NO_2_. Please refer to the S1 File for spatial maps of nine additional U.S. metropolitan areas (e.g., Atlanta, Houston, Los Angeles, New York, Philadelphia, Portland, San Francisco, Seattle, and Washington DC).

Overall, the RAMP Hybrid and Hybrid methods capture variability at significantly finer scales (∼0.01 km^2^) than CMAQ (12-km). To our knowledge, only a few studies have estimated PM_2.5_ and NO_2_ at census block scale across the U.S [39, 79]. These studies used statistical models and did not perform a health impact assessment. Please refer to S1 File, where we have quantified and compared to what resolution these methods capture within-cell variability.

As shown in Table 1, model performance between CMAQ and Hybrid is comparable. For PM_2.5_, the Pearson R^2^ is slightly higher for the Hybrid (0.32) method than CMAQ (0.30), while for NO_2_ CMAQ (0.63) is slightly higher than Hybrid (0.62). The mean error (ME) is higher for Hybrid for both pollutants, but Hybrid also shows that it captures higher variability having a higher standard deviation of the model values (SDZ). RAMP Hybrid shows better model performance for nearly every metric for both pollutants. Through bias correction RAMP Hybrid increases Pearson R^2^ by up to ~0.2 for both pollutants. One of the biggest successes from the RAMP Hybrid method is correcting the root squared mean error (RMSE) where CMAQ shows an RMSE of 2.29 and hybrid shows RMSE of 2.32 while RAMP Hybrid shows RMSE of 1.45 for PM_2.5_ and for NO_2_, CMAQ shows an RMSE of 3.66 and hybrid shows RMSE of 6.31 while RAMP Hybrid shows RMSE of 2.51.

**Table 1:** Model performance statistics for annual PM_2.5_ and NO_2_ for 2016 of CMAQ, Hybrid, and RAMP hybrid evaluated at AQS Sites across the continental United States. [PM_2.5_ (μg/m^3^): MO = 7.64, SDO = 2.06; NO_2_ (ppb): MO = 8.38, SDO = 5.73]. The best performing model metric has been highlighted when possible.

	Model	# Of Sites	FAC2 (%)	ME	SDE	RMSE	MZ	SDZ	Pearson R^2^	Spearman ρ^2^
PM_2.5_ (μg/m^3^_)_	CMAQ	928	95	**0.02**	2.29	2.29	7.67	2.64	0.30	0.35
	Hybrid		95	0.29	2.30	2.32	7.93	2.71	0.32	0.36
	RAMP Hybrid		99	0.08	**1.44**	**1.45**	7.72	1.64	**0.51**	**0.48**
NO_2_ (ppb)	CMAQ	444	85	-1.18	3.47	3.66	7.20	4.82	0.63	0.72
	Hybrid		85	2.61	5.36	6.31	10.99	9.04	0.62	0.76
	RAMP Hybrid		95	**0.05**	**2.51**	**2.51**	8.43	4.89	**0.81**	**0.84**

Note: FAC2 is percentage of modeled values between a factor of 2 of the observations; ME is the Mean of the Error; SDE is the Standard Deviation of the Error; RMSE is the Root Mean Squared Error; SDZ is the standard deviation of the modeled value; SDO is the standard deviation of the observed value; MZ is the mean of the modeled value; MO is the mean of the observed value

### Health risk assessment

**Fig 3:**
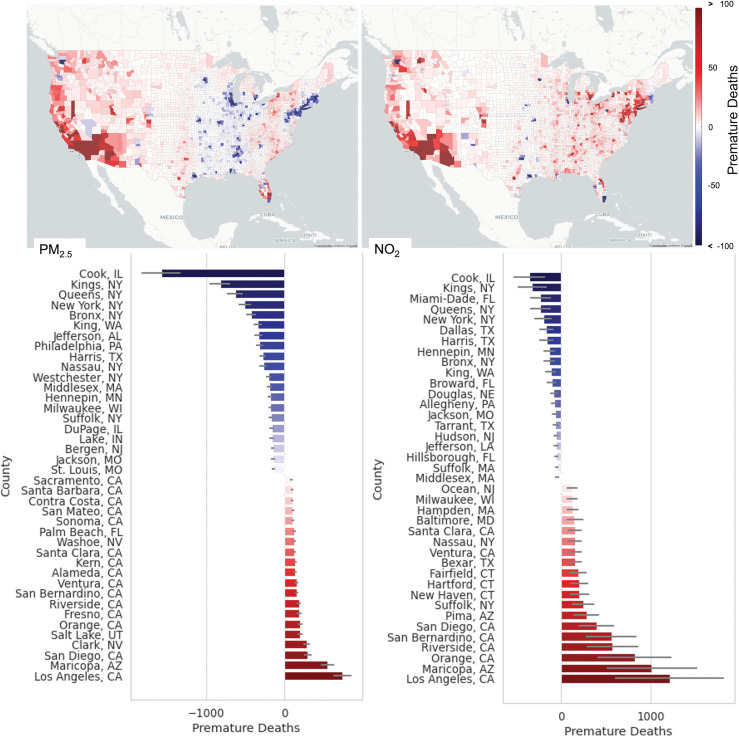
Premature mortality difference between RAMP Hybrid and CMAQ attributable to PM_2.5_ (left) and NO_2_ (right) aggregated by county. The Top Panel Show spatial differences across the continental United States. In the Bottom Panel, the blue bars represent the top 20 counties where RAMP Hybrid shows less premature mortality than CMAQ. The red bars represent the top 20 counties where RAMP Hybrid shows more premature mortality than CMAQ. The lines in each bar correspond to the 95% confidence intervals.

To estimate the difference between RAMP hybrid and CMAQ in premature mortality attributable to PM_2.5_ and NO_2_, we aggregated all-cause premature mortality for each county in Fig 3 in space. Results from this figure show that for PM_2.5_ the difference in premature mortality varies regionally. For PM_2.5_, several regions in the east of the U.S. estimate overall lower premature mortality for RAMP Hybrid than CMAQ while regions in the west show higher premature mortalities for RAMP Hybrid when compared to CMAQ. These differences between east and west are mainly due to biases in the CMAQ model (see S4 Fig in Model Performance section of S1 File for spatial maps of error). The hybrid method alone usually increases concentrations in urban areas near roads. So, the bulk of the regional differences are due to the RAMP correction rather than the hybrid method. For NO_2_, RAMP Hybrid estimates show lower premature mortalities than CMAQ mostly in counties from significantly large metropolitan areas (e.g., Boston, Chicago, Dallas, Houston, Miami, New York, Orlando, Pittsburg, Seattle, Tampa, and Portland).

The bar plots in Fig 3 shows the 40 counties that had the most difference in premature mortalities between RAMP Hybrid and CMAQ. In this figure, the red bars show the counties where RAMP hybrid estimated more premature mortalities when compared to CMAQ, and the blue bars represent the counties where RAMP Hybrid showed less premature mortalities when compared to CMAQ. For PM_2.5_, 15 out of the 20 counties that showed more premature mortalities in RAMP Hybrid when compared to CMAQ were in CA. Los Angeles County, CA was at the top where RAMP Hybrid showed 736 [622 856] more premature mortalities than CMAQ. 10 out of 20 counties where CMAQ showed the most premature mortalities when compared to RAMP Hybrid were in the Northeast. Cook County, IL (not in the northeast) was at the top where CMAQ estimated 1,574 [1,330 1,830] more premature mortalities than RAMP Hybrid. For NO_2_, regional patterns in difference in premature mortalities are not as evident. The counties where RAMP Hybrid showed the most premature mortalities when compared to CMAQ are almost evenly split between the east coast and the west coast. Los Angeles county still showed the most difference with 1,212 [95% CI, 605–1,827]. On the opposite side of the graph, CMAQ estimated that Cook County showed 354 [95% CI, 177–532] more premature mortalities than RAMP Hybrid. Additionally, most of the counties in the top 20 where CMAQ showed more premature mortalities were major metropolitan areas with dense populations (> 800 people/km^2^) and high road density.

The sum of differences in premature mortalities attributable to PM_2.5_ and NO_2_ between RAMP Hybrid and CMAQ show different findings depending on the pollutant and the distance from road. For PM_2.5_, CMAQ estimated 18,079 [95% CI, 15,276–21,023] more premature deaths than RAMP Hybrid (or 7% more premature mortalities than RAMP Hybrid) in some regions, and RAMP Hybrid estimated 15,310 [95% CI, 12,936–17,802] more premature mortalities than CMAQ (or 6% more premature mortalities than CMAQ) in other parts of the U.S. If we aggregate these values, we can estimate that overall CMAQ estimated 2,769 [95% CI, 2,340–3,221] more premature mortalities than RAMP hybrid. This corresponds to an overall ~1% decrease of estimated premature mortalities when estimating premature mortality with RAMP Hybrid when compared to CMAQ. This would seem counterintuitive, but at the core of the Hybrid method, we essentially displace concentrations in the grid away from roads to near the roads. This causes an increase in concentrations near roads while causing a decrease in concentrations away from roads. In the case of PM_2.5_, these competing effects result in a modest net difference. For NO_2_, CMAQ estimated 6,301 [95% CI, 3,150–9,451] more premature deaths than RAMP Hybrid in some regions, while RAMP Hybrid estimated 28,876 [95% CI, 14,438–43,315] more premature deaths than CMAQ in other regions. The net difference between these estimates correspond to RAMP Hybrid estimating 22,576 [95% CI, 11,288–33,864] more premature mortalities attributable to NO_2_ than CMAQ. This corresponds to an overall ~19% increase in estimated premature mortalities due to NO_2_ using RAMP Hybrid when compared to CMAQ.

To assess the effect of only fine-scale variability on risk assessment, we re-gridded our RAMP Hybrid estimates to match CMAQ’s 12-km resolution. This type of analysis comparing RAMP Hybrid vs RAMP Hybrid at 12-km resolution (RAMP Hybrid 12-km) shows a different distribution of premature mortalities. Across the U.S. for PM_2.5_, RAMP Hybrid 12-km estimated 3,203 [95% CI, 2,706–3,725] more premature deaths than RAMP Hybrid, while RAMP Hybrid estimated 1,975 [95% CI, 1,669–2,297] more premature deaths than RAMP Hybrid 12-km (or ~1% more premature mortality than the coarser model). Again, this might seem counterintuitive, but because on-road primary PM_2.5_ corresponds to a small fraction of all the ambient PM_2.5_, the difference between coarse scale and fine-scale RAMP Hybrid is modest. If we aggregate these values, we can estimate that the coarser RAMP Hybrid estimated 1,228 [95% CI, 1,037–1,428] more premature mortalities than the finer scale RAMP hybrid, but these changes represent less than 1% of the total premature mortality calculated. This implies that the difference between fine-scale and coarse scale RAMP Hybrid are minor. For NO_2_, RAMP Hybrid 12-km estimated 10,065 [95% CI, 5,033–15,098] more premature mortalities than the fine-scale RAMP Hybrid. This is equivalent to 7% more premature mortalities estimated by the coarser resolution RAMP Hybrid. Alternatively, the fine-scale RAMP Hybrid estimated 4,854 [95% CI, 2,427–7,281] more premature deaths than RAMP Hybrid 12-km. Overall, the coarser scale model estimated 5,211 [95% CI, 2,606–7,817] more premature deaths attributable to NO_2_ than fine-scale RAMP Hybrid. Furthermore, focusing on the premature deaths near major roads, we find that most of the excess premature deaths shown by the RAMP Hybrid model are within 500 m from the road (92% for PM_2.5_ and 98% for NO_2_). At distances of less than 125 m, the net premature differences between coarse and fine-scale RAMP hybrid flip, where the fine-scale RAMP hybrid estimates more premature mortality than the coarse RAMP hybrid for both PM_2.5_ and NO_2_. This is the outcome that is expected near roads. Please refer to S1 File for detailed tables of differences in premature mortality between the methods at varying distances from the road.

**Fig 4:**
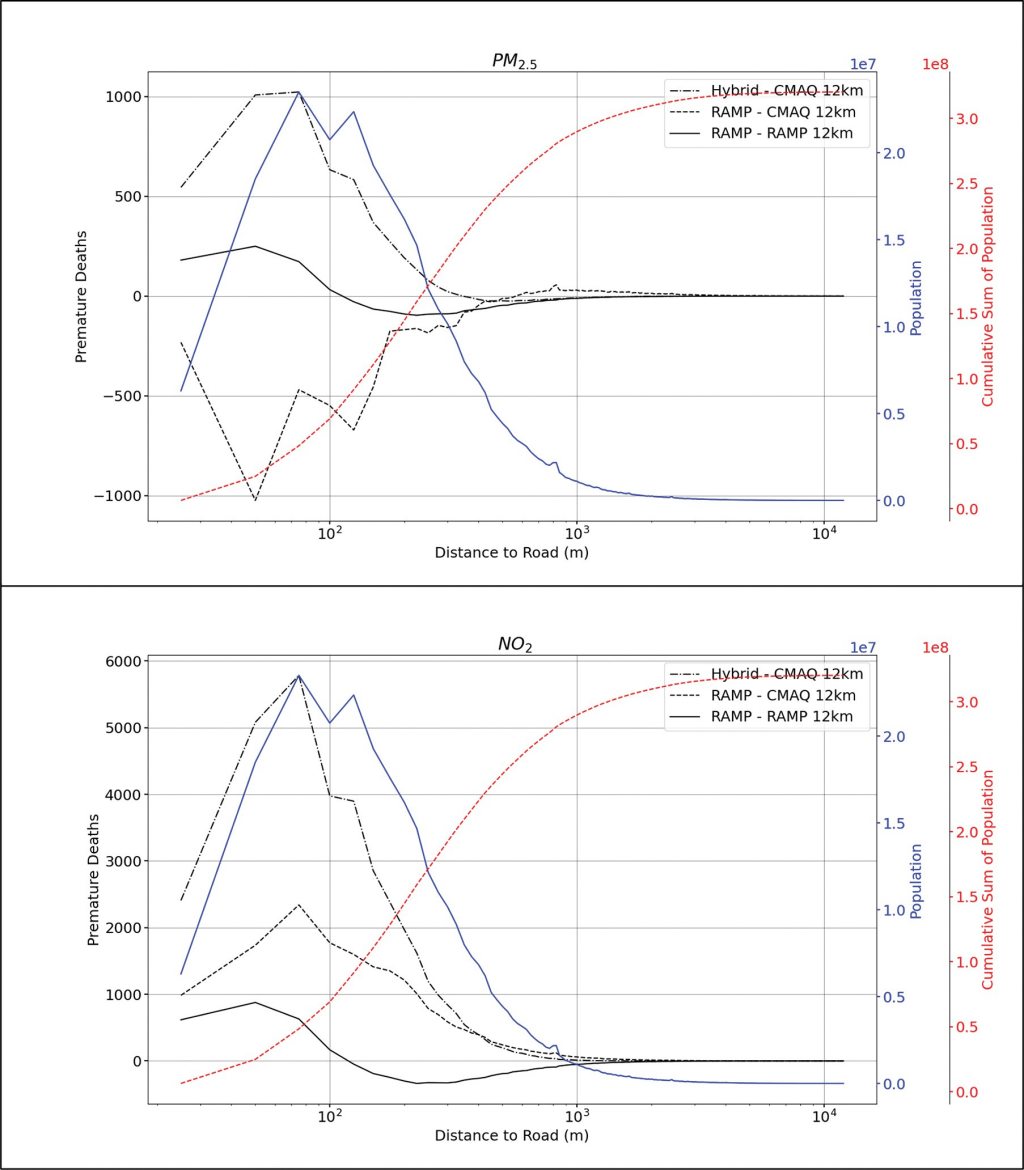
Net difference in premature mortality for PM_2.5_ and NO_2_ between models with varying resolution vs distance from primary road. Net premature mortality was aggregated at every 25 m from a primary road. The blue line represents the population aggregated at every 25 m from a primary road and the red line corresponds to the cumulative sum of the population.

Fig 4 compares the difference of premature mortality attributable to PM_2.5_ and NO_2_ between our models (e.g., Hybrid, CMAQ, and RAMP Hybrid) across the U.S. as a function of distance from road. The dashed-dot curves show net premature mortality difference between Hybrid-only minus CMAQ. The dashed curves show net premature mortality difference between RAMP Hybrid minus CMAQ. We have also added a blue line that represents the population aggregated at every 25 m from the road and a red line corresponds to the cumulative sum of the population. Most of the differences in premature mortality occur at census blocks that are less than 500 m from the road, as mentioned previously. Additionally, we can see that RAMP correction from Hybrid vs CMAQ (dashed-dot) to RAMP Hybrid vs CMAQ (dashed) causes a downward shift of the premature mortality difference for both pollutants. PM_2.5_ shows negative net premature mortality at census blocks less than 500 m from a major road which corresponds to more premature deaths estimated with CMAQ compared to RAMP Hybrid. For NO_2_, there is also less premature deaths difference between Hybrid and RAMP Hybrid, but the net difference is still positive. For both pollutants, these changes are mostly driven by RAMP rather than the effect of finer resolution due to hybrid as can be seen from the solid curve (RAMP Hybrid vs coarse RAMP Hybrid) comparison which shows smaller net differences and shows only the effect of resolution. Additionally, the solid curve shows positive net differences at census blocks closer to the road between the fine-scale and the coarse-scale RAMP hybrid which correspond to more premature mortality estimated by the fine-scale RAMP-hybrid near the road. This positive net difference occurs for ~80 million people for both PM_2.5_ and NO_2_, as seen from the cumulative population curve. Additionally, the positive net difference flips (to negative) at ~125 m from major roads. Thus, at census blocks at a distance > 125 m from major roads (where most of the population resides), we estimate higher premature mortality with coarse-scale RAMP Hybrid. For concentration differences between models instead of premature mortality differences, please refer to S1 File.

### Exposure inequity

Using the RAMP hybrid method, we calculated population weighted exposure for the entire population in the continental US, and we also stratified population-weighted exposure by race. With these, we calculated an exposure inequity ratio (EIR) which corresponds to the population weighted exposure of the Minority group (corresponds to all the non-White population) over the population weighted exposure of the White group for the continental US. The EIR ratio allows us to quantify to what degree Minorities are affected by PM_2.5_ and NO_2_ pollution when compared to their White counterparts. The EIR shows Minorities are exposed to 11% more PM_2.5_ and 39% more NO_2_ than the White population. See S1 File for details. To our knowledge this is the first time that this ratio has been applied using census block level resolution from a hybrid model that incorporates a CTM, dispersion model, and bias correction through RAMP.

Exposure to PM_2.5_ and NO_2_ varies spatially. Thus, we aggregated population weighted exposure by county across the U.S (see S1 File for map). For PM_2.5_, the highest concentrations are clustered in California with levels of ~12 μg/m^3^ or higher. These concentrations are much higher than in the rest of the country where concentrations gradually change across space with a maximum of about ~9 μg/m^3^ in the Midwest. Similarly, for NO_2,_ the highest concentrations are predicted in southern California, Colorado (specifically the Denver area), and New York/New Jersey with concentrations that are at least 15 ppb, while in the rest of the country concentrations max out at ~10 ppb in other big cities or across sizeable highways. To evaluate exposure inequity, we must determine if the population is distributed across the country in a stratified way across racial groups, since that could lead to inequity when large proportions of Minorities reside in areas with high air pollution. Analyzing the proportion of Minority groups in space, it becomes apparent that the Asian population and the Hispanic population are clustered in California where we saw higher concentrations of PM_2.5_ and NO_2_. This disproportionate distribution of Minorities residing in areas with high air pollution also extends to the Black population near the New York City area when considering NO_2_. The combination of localized minorities and localized high air pollution begins to show an expected geographical inequity nationwide because of the distribution of these populations across the nation. Please refer to S1 File for a spatial map of the proportion of minorities by county.

Using the hybrid RAMP model, we can explore EIRs at scales even finer than the county level. Table 2 shows the distribution of EIRs for PM_2.5_ and NO_2_ at County, Census Tract, and Census Block Group levels. As expected, when looking at the mean of EIRs overall, the finer the geographic unit, the smaller the exposure inequity ratio is for both PM_2.5_ and NO_2_. This is expected given that as we consider smaller geographic groupings, we expect less changes in population proportions or in air concentrations. Nonetheless, there is still exposure inequity in some counties, census tracts and census block groups as shown in the higher percentiles of Table 2. EIRs can be as high as 1.05 for PM_2.5_ and 1.4 for NO_2_ at some census tracts and 1.04 for PM_2.5_ and 1.36 for NO_2_ in some census block groups.

**Table 2:** Distribution of exposure inequity ratio estimated at county level, census tract level, and census block group level for PM_2.5_ and NO_2_.

Pollutant	Metric	County	Census Tract	Census Block Group
**PM**_**2.5**_	**Count**	3,107	71,916	215,071
	**Mean**	1.0074	1.0010	1.0006
	**STD**	0.0146	0.0059	0.0049
	**50%**	1.0044	1.0005	1.0002
	**95%**	1.0324	1.0088	1.0067
	**99%**	1.0562	1.0200	1.0162
	**99.9%**	1.1109	1.0493	1.0400
**NO**_**2**_	**Count**	3,107	71,916	215,071
	**Mean**	1.0669	1.0104	1.0060
	**STD**	0.0923	0.0494	0.0410
	**50%**	1.0502	1.0038	1.0015
	**95%**	1.2358	1.0712	1.0582
	**99%**	1.3544	1.1659	1.1398
	**99.9%**	1.6124	1.4048	1.3566

**Fig 5:**
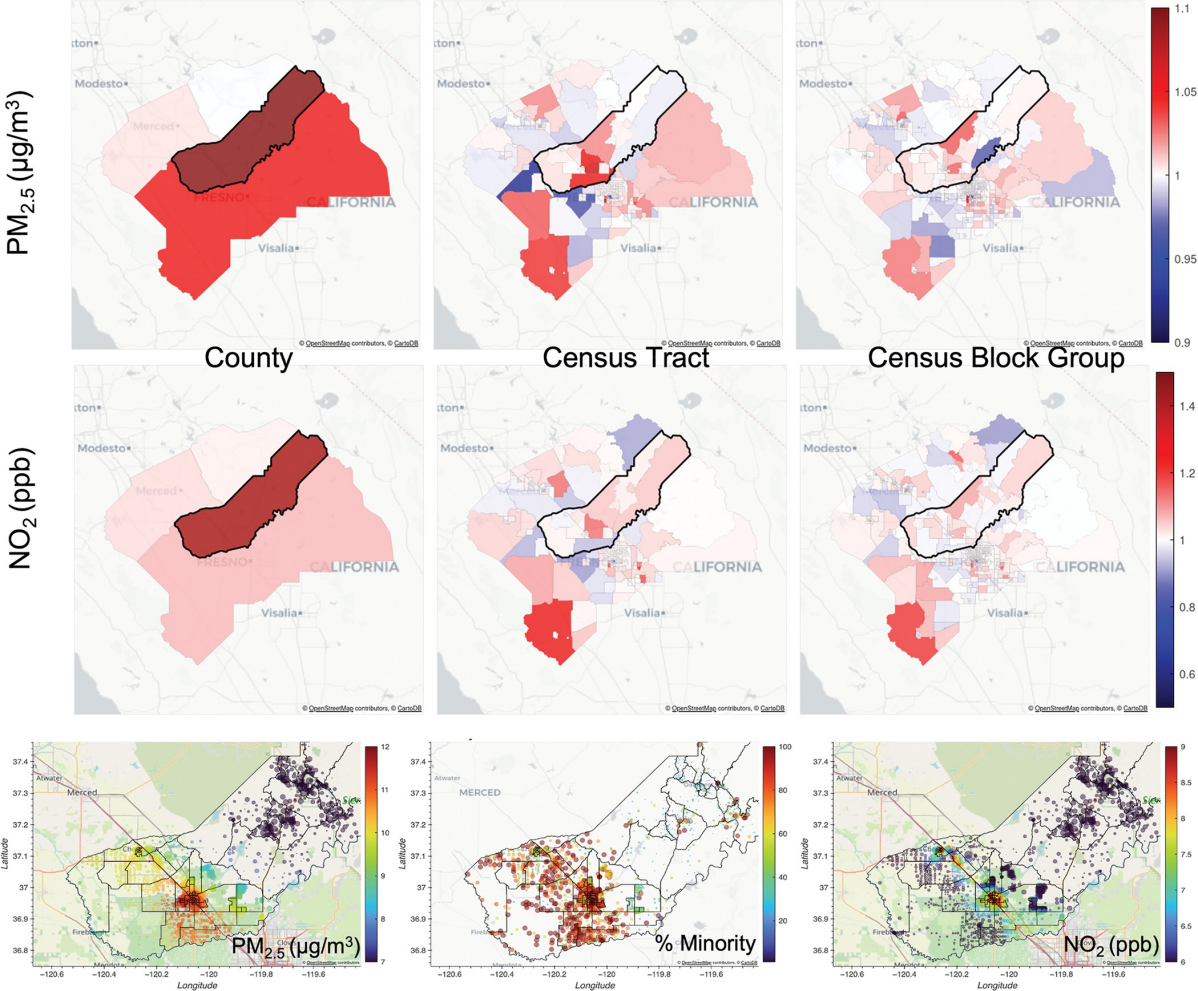
Exposure inequity ratio (EIR) at Madera County, CA. Top panel shows Exposure Inequity Ratio (EIR) at Madera County, CA (highlighted) and surrounding counties for PM_2.5_ and NO_2_ at County (left), Census Tract (middle), and Census Block Group level (right). Bottom panel shows RAMP Hybrid concentration as circles for PM_2.5_ (left) and NO_2_ (right) at census block centroids where the size of the census block centroid is proportional to population, as well as the percent of Minority population (middle) at census block centroids where size of census block is proportional to percent Minority at Madera County, CA.

Zooming into the effect of exposure inequity in a particular county, one can focus on counties with highest EIR like Madera, CA. This county has the highest EIR for both PM_2.5_ and NO_2_ among counties with a population greater than 100,000 (see S1 File for the Table listing the top 10 counties by EIR). As shown in the top panel of Fig 5, Madera County has strong exposure inequity patterns that explain within county EIR. Madera County has a high EIR of 1.2 for PM_2.5_ and 1.44 for NO_2_, at the county aggregation level, but also has high EIR within the county at the census tract and census block group aggregation levels. Focusing on PM_2.5_, the two census block groups that show the highest EIR and the lowest EIR in the middle of Madera County can help explain the effect of air pollution and distribution of the Minority population on EIR. The bottom panel of Fig 5 shows that for PM_2.5_, there are higher concentrations near the city of Madera. Furthermore, even though there is a significant amount of the population residing away from the city, the percent of Minority map shows that this population is predominantly White. Thus, when looking at the two census block groups mentioned previously, the census block group in the north with Minorities closer to Madera city has an EIR greater than 1 while the census block group in the south with a higher proportion of the White population away from Madera city has an EIR less than 1. For NO_2_, there is a similar pattern for these two census block groups. The magnitude of the EIR is larger and the effect of NO_2_ is more localized and closer to the roads as is shown in the bottom panel of Fig 5, where the highest concentrations follow Highway 99.

**Fig 6:**
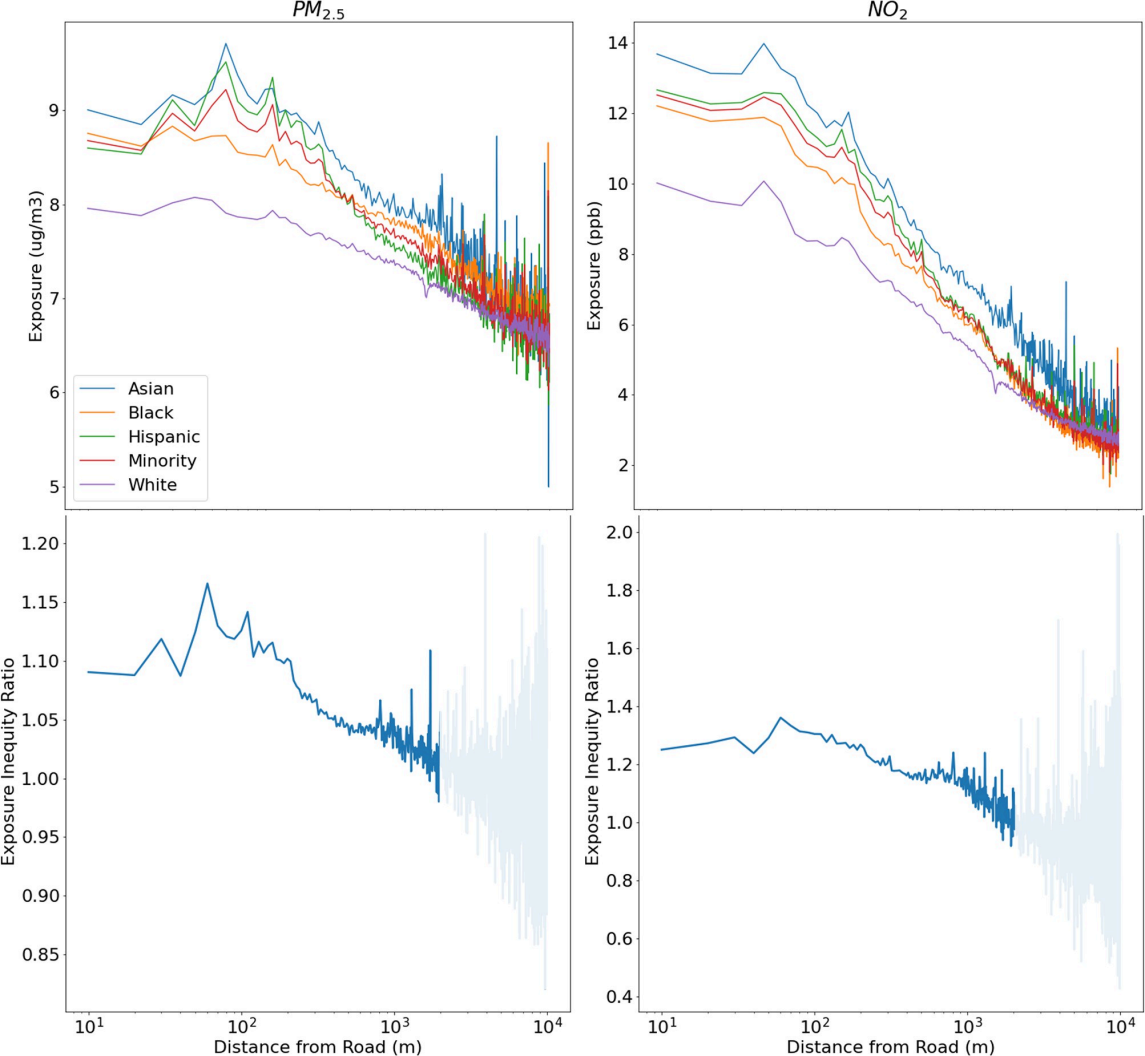
Population-Weighted Exposure using RAMP (top) for PM_2.5_ (left) and NO_2_ (right) and Exposure Inequity ratio (bottom) aggregated at 10m from major roads across the continental U.S. In the bottom two frames, EIRs has been blurred at distances greater than 2 km from the road to convey that there is high noise in the EIR results.

To follow the near-road effect nationwide, we have created Fig 6 which shows the population-weighted exposure using RAMP aggregated for every census block at every 10m from primary roads across the continental U.S. As mentioned previously, we can see how the air pollution gradients differ between PM_2.5_ and NO_2_ near road, where NO_2_ has a steeper decline of concentration when moving away from the road. This figure also shows that not only are Minorities exposed to higher air pollution than their White counterparts in the corridor very near (i.e., within 10 m of) roads, but this exposure inequity persists even at distance of 100m to 1km of roads, where Minorities can be exposed to up to 15% more PM_2.5_ than the White population and up to 35% more NO_2_ than the White population (bottom panel of Fig 6). This shows that even when controlling for distance to road, the EIR is greater than 1 for hundreds of meters away from roads, which indicates that exposure inequity is pervasive and entrenched across the nation.

## Discussion and conclusions

### Health assessment comparison with previous studies

Across the continental U.S., we estimate the total burden of premature mortalities attributable to all sources of ambient PM_2.5_ (anthropogenic and nonanthropogenic) ranges between 223,506 and 307,577 using the RAMP Hybrid approach (225,846 and 310,797 for CMAQ). These premature mortality estimates fall within those of other studies. For example, Davidson et al. [30] estimate between 100,000 and 220,000 premature mortalities were associated with mobile and non-mobile sources of PM_2.5_ in 2011. Tessum et al. [80] estimated that 102,000 premature mortalities were associated with anthropogenic PM_2.5_ in 2015. Dedoussi et al. [31] estimated that the U.S. combustion emissions of PM_2.5_ caused premature mortalities between 62,400 and 104,200 in 2011, and 49,300 and 82,900 in 2018. There are several differences between our study and the previously mentioned studies. These differences include the air quality model used, model resolution, source types considered, health incidence data resolution, causes of mortality (all cause vs specific causes), and, more importantly, varying CRFs.

In our study, the health impact estimates were based on premature mortality estimates from the most updated PM_2.5_ mortality CRF from a recent meta-analysis [73]. This CRF was based on newer PM_2.5_ studies conducted at lower and at higher concentrations of PM_2.5_ than previous studies. This CRF reported higher slopes at low concentrations which estimated higher mortality. Furthermore, a study by Burnett et al. [81], compared their earlier integrated exposure–response (IER) function [82] to a newer IER including newer studies. Their findings suggest that the newer IER produces similar results to ours (~210,000 all-cause premature mortalities attributable to PM_2.5_ across the U.S.). Burnett et al. compared the updated estimates with those of their older IER [82] and found that the updated CRF estimates were 2.22 times larger than with the older IER. They also compared the updated IER that considered all-cause mortality to the updated IER that considered only five separate causes. The IER that considered all-causes estimated 1.75 times more premature mortality than the one with five separate causes. This study [81] emphasizes a potential underestimation of premature mortality when using older CRFs or not considering all-cause mortality.

Providing a more even comparison, the Vodonos et al. [34] study which applies a similar CRF as our study estimated that premature mortalities would fall by 104,786 [57,16 135,726] attributable to a reduction of 40% of PM_2.5_. If we estimate the proportion of total deaths without reduction for the Vodonos et al. study, this is equivalent to 261,965 [142,540 339,315] premature mortalities for 2015 which compares well to our estimate of 264,516 [223,506 307,577] for RAMP Hybrid in 2016. This last estimate provides us with the most confidence in the magnitude of the effects estimated from our study for PM_2.5_.

For NO_2_, most studies have only estimated the total burden of diseases in smaller domains (e.g., Hong Kong [83], San Francisco Bay Area [21]) and to our knowledge no studies have performed a health risk assessment based upon premature mortality due to NO_2_ across the U.S. This might be in part due to the active discussion on the independent causal relationship between long-term NO_2_ and mortality. Nonetheless, meta-analysis has determined that there are likely causal relationships for long-term exposure to NO_2_ and premature mortality [74, 84–86]. Overall, in our current study, we estimate 138,550 [69,275 207,826] premature mortalities attributable to NO_2_ using RAMP Hybrid compared to 115,975 [57,987 173,961] using CMAQ.

### RAMP hybrid data fusion findings

We correct biases in the model through the RAMP approach and overall, our findings show that the difference in premature mortality between CMAQ at 12-km resolution and RAMP Hybrid at census blocks are mostly due to bias correction. When we compare RAMP Hybrid at fine-scale resolution vs RAMP hybrid 12-km, we find that the difference in premature mortality is <1%, suggesting that at a 12-km resolution annual PM_2.5_ can be captured adequately. Nonetheless, we do see a noticeable increase of exposure near the road. For NO_2_, given that on-road emission contributions are much higher than PM_2.5_ to the total anthropogenic emissions, the comparison between RAMP at fine-scale resolution and coarse-scale resolution shows bigger differences. The RAMP Hybrid shows around ~4% more premature mortality attributable to NO_2_ across the U.S. than RAMP Hybrid 12-km. RAMP Hybrid 12-km estimates ~7% more premature deaths than the fine-scale RAMP Hybrid at all distances from road. So, the overall net differences in premature mortality for NO_2_ is higher for the coarse RAMP Hybrid (~4%) than at with the fine-scale RAMP Hybrid. This finding is supported by Batterman et al.’ s (2014) Detroit, MI fine-scale study. In this local scale study, they conclude that near-road dispersion modeling at coarser resolution than census block (e.g., census tract and ZIP code levels) have the tendency to overestimate average exposures because at this scale the range of concentrations would be compressed, a smaller amount of individuals reside “very near major roads”, most exposures would be misclassified, and higher concentrations near roads would be excluded. It is of note that the Batterman et al. (2014) study compares R-LINE exposure (population-weighted) concentrations of NOx at census blocks to R-LINE at census tracts. Our analysis compares RAMP Hybrid premature mortality attributable to PM_2.5_ and NO_2_ at 12-km resolution to RAMP Hybrid at census block. However, our overall findings would not change significantly when comparing RAMP Hybrid premature mortality at census block to RAMP Hybrid at census tract. The magnitude of overestimation from the coarser scale averaged grid results would decrease by 5% for NO_2_ and 10% for PM_2.5_ (see S1 File for details). Another significant difference highlighted here is that the Batterman et al.’s study focuses only on NO_x_ concentrations/exposures from on-road emissions and does not perform a health risk assessment. Thus, their conclusions are based on population-weighted concentrations.

In general, our findings show that the RAMP Hybrid method can provide reliable estimates for both PM_2.5_ and NO_2_ that capture the near-road gradient in urban environments. These estimates produce reliable exposure estimates that can reduce exposure misclassification and can ultimately provide reliable inputs to individual-level exposure models which will improve risk assessment for epidemiological studies.

### Exposure inequity comparison with previous studies

We have shown how a coarse model (12-km resolution) can overestimate NO_2_ and PM_2.5_ concentration and mortality associated with these pollutants away from the road while underestimating these near major roads when compared to the fine-scale RAMP hybrid method. This phenomenon affects a significant amount of the population that are exposed to these pollutants and can only be captured with the fine-scale RAMP model. This has implications regarding exposure inequity given that, as mentioned previously, a significant amount of the Minority population resides near major roads. In fact, the proportion of Minorities that live near (~200 m) major roads is higher than the proportion of the White population (See S1 File). Other studies have shown that the Minorities are exposed to more PM_2.5_ and NO_2_ than their White counterparts [13–17, 20, 22, 87]. For example, a study by Clark et al. (2014) suggested that Minorities were exposed to 38% more NO_2_ than their White counterparts using estimates at census tract for 2006. A more recent study that estimates several pollutants at census blocks from 1990 to 2010 showed that nationwide in 2010, the Minority populations were exposed to 13% more PM_2.5_ and 54% more NO_2_ than the White population [13]. A similar study that looked at the effect of data aggregation at different spatial resolutions on exposure inequity reinforced that exposure inequity estimated at state and county scales underestimates disparity when compared to census tract or finer scales [79]. A nationwide study at ZIP code level estimated that in 2016, Minority populations had ~12% higher exposure of PM_2.5_ when compared to the White population [14]. Most of these studies implement national empirical statistical models which do not consider all local sources of air pollution to obtain air pollution exposure. Thus, they overlook the physical/chemical processes and the fine-scale effect of road sources that can better quantify emission contribution at hyperlocal scales. Using our RAMP hybrid method which considers these processes, we have estimated that Minorities are exposed to 11% more PM_2.5_ and 39% more NO_2_ than the White population across the US. The hyper local resolution of our model on a nation-wide basis allows us to examine and, for the first time, visualize these inequities meters away from the road. Our method reveals that within 100 meters from major roads, Minority populations can be exposed to up to 15% more PM_2.5_ and up to 35% more NO_2_ than their White counterparts.

Furthermore, recent studies have shown that fine-scale air pollution estimates of NO_2_ and PM_2.5_ can be used to identify discriminatory policies that impacted environmental exposure inequity [17]. This study leverages land use regression models to show how redlining in the U.S. disproportionately impacts Minority populations. Likewise, we saw from our results, that the fine-scale RAMP hybrid which leverages the chemical/transport process in the atmosphere showed exposure inequity that persists at the census block level. Moreover, not only are we aware that there is an increased proportion of Minorities near the road, but our results also show exposure inequity continues to persist within close vicinity of the road (~10 m) and up to 100 m from the road. Thus, other factors such as traffic magnitude, weather patterns, and certain policies can contribute to this exposure inequity. Using our novel method will allow us to better estimate, identify, and address the exposure inequity near roads that occurs at the finest scales.

### Limitations and future work

The present work is subject to several uncertainties and limitations. Both the dispersion model and the CTMs involve several assumptions/parameters and input data (e.g., emissions inventory, meteorological modeling) that have considerable uncertainties especially at fine-scale resolution. For example, we are using CMAQ v5.2.1 (latest available at the time of this study) which does not include additional aerosol updates with updated secondary aerosol chemistry. As mentioned before, R-LINE tends to overestimate under low dispersion conditions. Thus, both models have different biases that the Hybrid approach does not consider individually. Other studies have tried to treat the biases before combining these models [45]. However, for correcting R-LINE biases, this required detailed collocated near-road monitoring data for NO_2_, NOx, CO, and SO_2_ that is not readily available across the continental U.S. [88]. By correcting model biases before combining CMAQ and R-LINE, another study developed a multiplicative hybrid method [45]. In this study, this method was recommended for gaseous pollutants to avoid negative estimates of CMAQ without major roads. We attempted to implement this method for NO_2_. But since we could not correct biases before applying the hybrid method, this resulted in unrealistic hybrid estimates. Thus, we relied on RAMP to correct biases after combining the models with our additive method as other studies have also done [68]. Our RAMP method is an effective (shows good model performance) and computationally efficient method to account for the biases after the Hybrid method has been applied. Furthermore, for our analysis we are predicting concentrations at census block levels which also has uncertainties. This is the smallest geographic unit where population data is available. However, even at this scale, Batterman et al. (2014) found some effects of exposure misclassification (but less than at census tract and ZIP code level).

For our health impact assessment, we assume that an individual’s exposure is mainly representative of census block where they reside and only represents exposure when they are in their census block. A significant amount of the population travels outside of their census block (e.g., commuting for work or school) and this can change their level of exposure. Additionally, when applying our CRF, we use county-based health incidence data. But other studies have found that using county level health incidence data underestimates the risk when compared to ZIP code level [34]. Future work might explore health outcomes using higher resolution health incidence data. Additionally, given that our modeling approach can estimate short-term exposure, future work might also explore daily exposure of PM_2.5_ and NO_2_ to expand our analysis to finer temporal scales, and assess acute health impacts.

Finally, an aspect of the RAMP method not considered in this analysis is that it can consider the non-homoscedasticity between modeled and observed data. By considering the non-homoscedastic behavior of model performance we can assess how the error variance changes among predicted values, and ultimately assign a variance (i.e., uncertainty) to our predictions. Furthermore, once we apply a RAMP framework that considers the non-linear, non-homogeneous, and non-homoscedastic behavior of model performance, we can further improve model performance through the full RAMP Bayesian Maximum Entropy (BME) data fusion method [57].

## Supporting information

S1 File (DOCX)

S2 File (ZIP)
