# Supplementary material for: A hyperlocal hybrid data fusion near-road PM2.5 and NO2 annual risk and environmental justice assessment across the United States
Source: PLoS One. 2023 Jun 1;18(6):e0286406. doi: 10.1371/journal.pone.0286406 (PMC10234552; doi:10.1371/journal.pone.0286406)
Supplement: S1 File — (DOCX) [file pone.0286406.s001.docx]

A hyperlocal hybrid data fusion near-road PM_2.5_ and NO_2_ risk and environmental justice annual assessment across the United States

Supporting information

Alejandro Valencia^1,2^, Marc Serre^1^ and Saravanan Arunachalam^2*^

^1^ Department of Environmental Sciences and Engineering, The University of North Carolina at Chapel Hill, Chapel Hill, North Carolina, United States of America

^2^ Institute for the Environment, The University of North Carolina at Chapel Hill, Chapel Hill, North Carolina, United States of America

* Corresponding author

E-mail: sarav@email.unc.edu (SA)

Table of Contents

[Reference Map 2](#_Toc133996535)

[RAMP Hybrid and Model Performance Analysis 3](#_Toc133996536)

[Health Risk Assessment 13](#_Toc133996537)

[Environmental Justice Assessment 19](#_Toc133996538)

[References 27](#_Toc133996539)

#
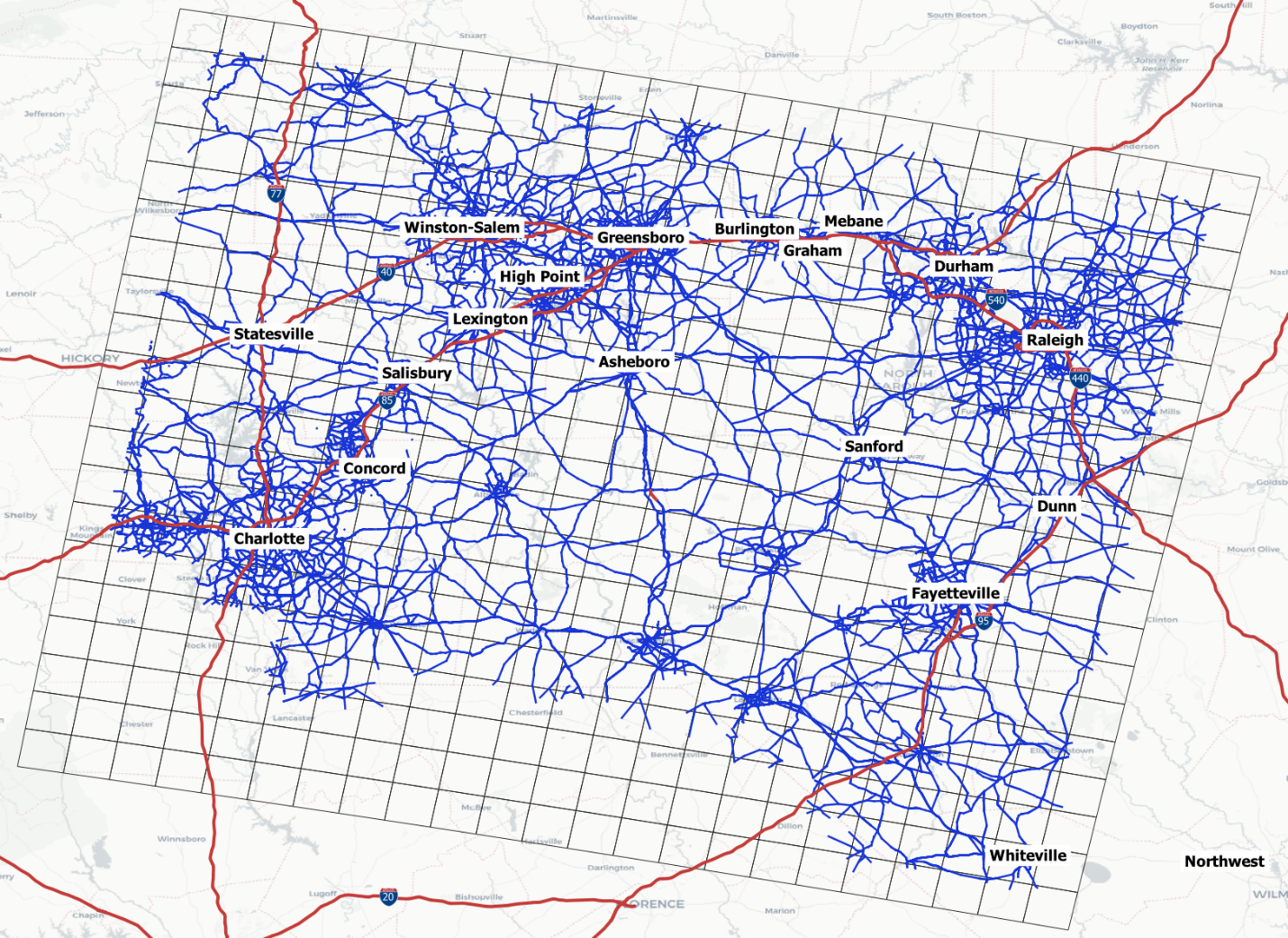
Reference Map

#### S1 Fig. Central North Carolina map. Blue lines represent major roads used for fine-scale modeling. Red lines represent interstates Black grid is 12 km x 12 km CMAQ grid resolution.

# RAMP Hybrid and Model Performance Analysis

Mean of the Observations:$MO=\frac{1}{N}\sum_{i=1}^{N} (z_{i})= \bar{z_{i}}$ (1A)

Mean of the Estimates:$MZ=\frac{1}{N}\sum_{i=1}^{N} (\tilde{z}_{i})=\bar{\tilde{z}_{i}}$ (2A)

Mean Error:$ME=\frac{1}{N}\sum_{i=1}^{N} (\tilde{z}_{i}-z_{i})$ (3A)

Variance of the Observations:$VO=\frac{1}{N-1}\sum_{i=1}^{N} (z_{i}-\bar{z_{i}})$ (4A)

Variance of the Estimates: $VZ=\frac{1}{N-1}\sum_{i=1}^{N} (\tilde{z}_{i}-\bar{\tilde{z}_{i}})$ (5A)

Variance of the Error: $VE=V(\tilde{z}_{i}-z_{i})$ (6A)

Predictions Within a Factor of Two of Observations:

$FAC2=fraction where 0.5 \leq\frac{\tilde{z}_{i}}{z_{i}} \leq2.0$ (7A)

Correlation: $R=\frac{\sum_{i=1}^{N} \left( \tilde{z}_{i}-\bar{\tilde{z}_{i}} \right)\left( z_{i}-\bar{z_{i}} \right)}{\sqrt{\sum_{i=1}^{N} \left( \tilde{z}_{i}-\bar{\tilde{z}_{i}} \right)^{2}}\sqrt{\sum_{i=1}^{N} \left( z_{i}-\bar{z_{i}} \right)^{2}}}$ (8A)

Correlation Squared:$R^{2}=R^{2}\left( \tilde{z}_{i}{,z}_{i} \right)$ (9A)

To quantify to what resolution the RAMP Hybrid model captures variability, we have created Figure S2. In this figure, we gridded the census block data with varying cell sizes from 64 km to 200m. We then calculated the standard deviation inside each grid cell. The solid line shows the median standard deviation (SD) across the U.S. for that cell size (the fill shows the 25^th^ and 75^th^ percentile of the SD). So, for example because CMAQ has a grid resolution of 12 km x 12 km there is no variability within a 12 km x 12 km cell size. For PM_2.5_, the within-cell SD for RAMP hybrid remains on par with CMAQ up until 12 km x 12 km. RAMP hybrid shows slightly less variability than the Hybrid method. Both methods retain some degree of variability (SD between 0.02 and 0.01 μg/m^3^) from 64 km x 64 km up until 200 m x 200 m grid resolution. For NO_2_, RAMP Hybrid and Hybrid show comparable variability down to 200 m x 200 m (0.3 ppb at 200m). Both these models show greater SD than CMAQ across all cell sizes. In other words, for both pollutants, CMAQ at 12 km x 12 km resolution lacks the ability to capture in cell variability (i.e., changes in concentrations) at cell sizes smaller than 12 km x 12 km while RAMP hybrid can capture variability at resolutions of a few hundred meters or less, given that in urban areas census block are ∼0.01 km^2^.


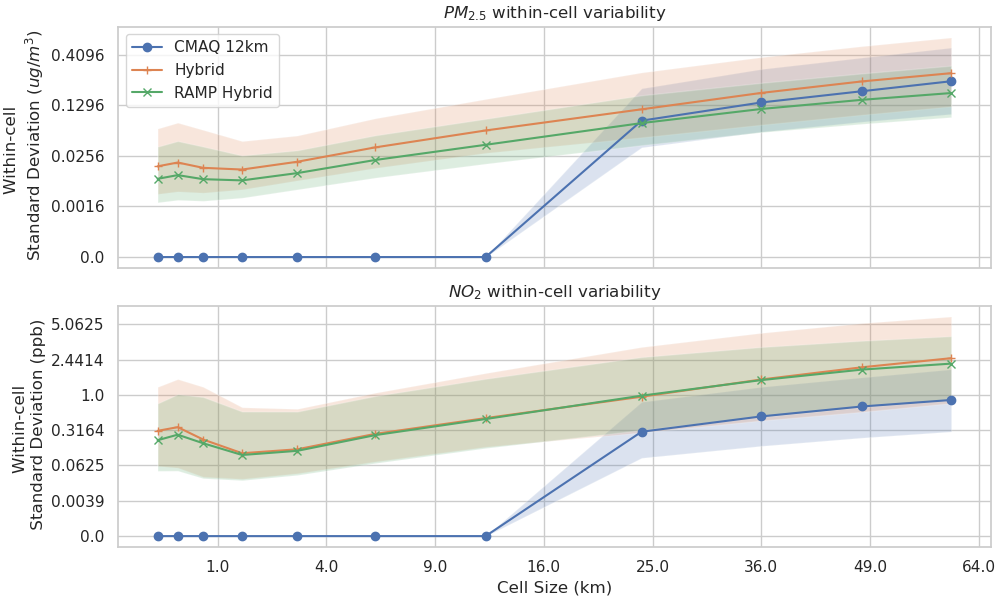


#### S2 Fig. Within-cell variability across the continental United States for PM_2.5_ and NO_2_. The solid lines represent the median standard deviation at a given cell size for CMAQ, Hybrid, and RAMP Hybrid for the domain. The fill that bound each line represents the 25^th^ and 75^th^ percentile of the standard deviation.

####
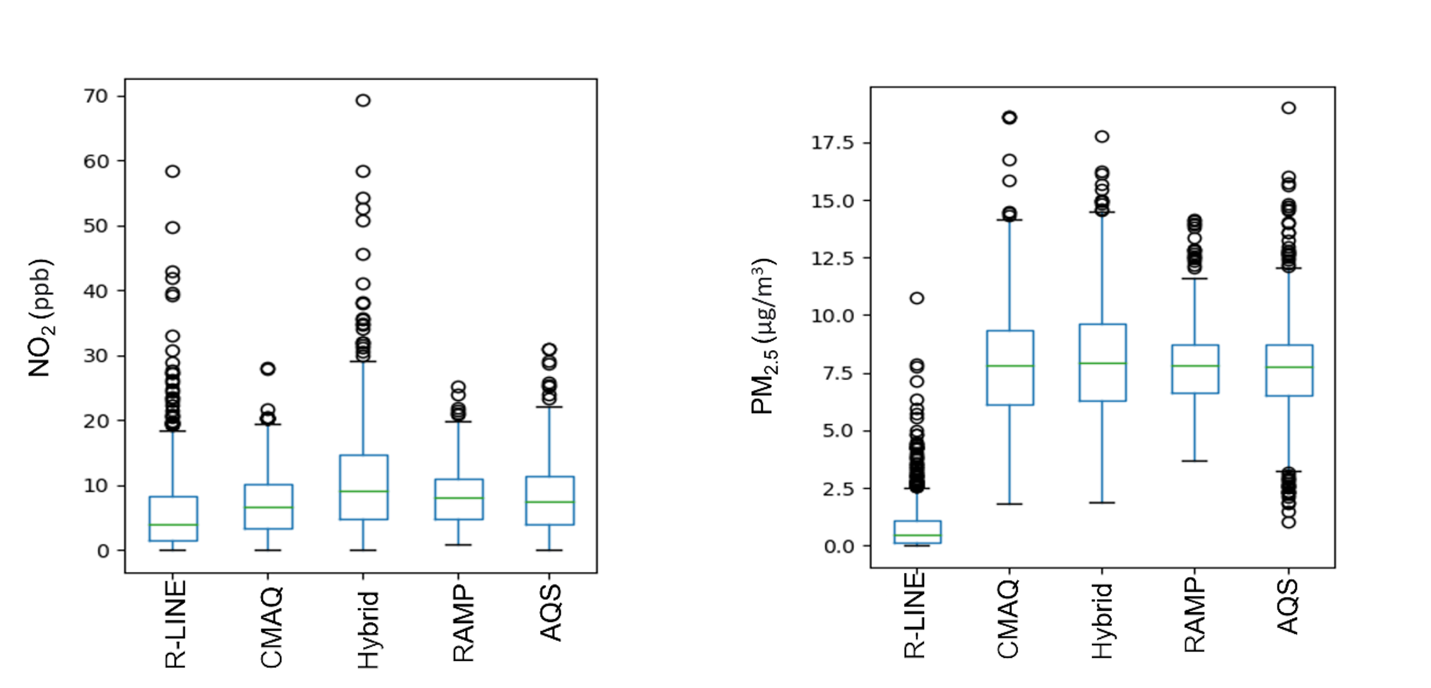
S3 Fig. Annual distribution of concentrations at AQS sites for PM_2.5_ (μg/m^3^) and NO_2_ (ppb). For R-LINE, CMAQ, Hybrid and RAMP Hybrid. The box represents the middle 50% of the data, extending from the 25^th^ to the 75^th^ percentiles; the horizontal line through the center of the box is the median; the whiskers represent 1.5*IQR (the inter-quartile range is the range from the 25^th^ to 75^th^ percentiles); the points are outliers above and below 1.5*IQR.


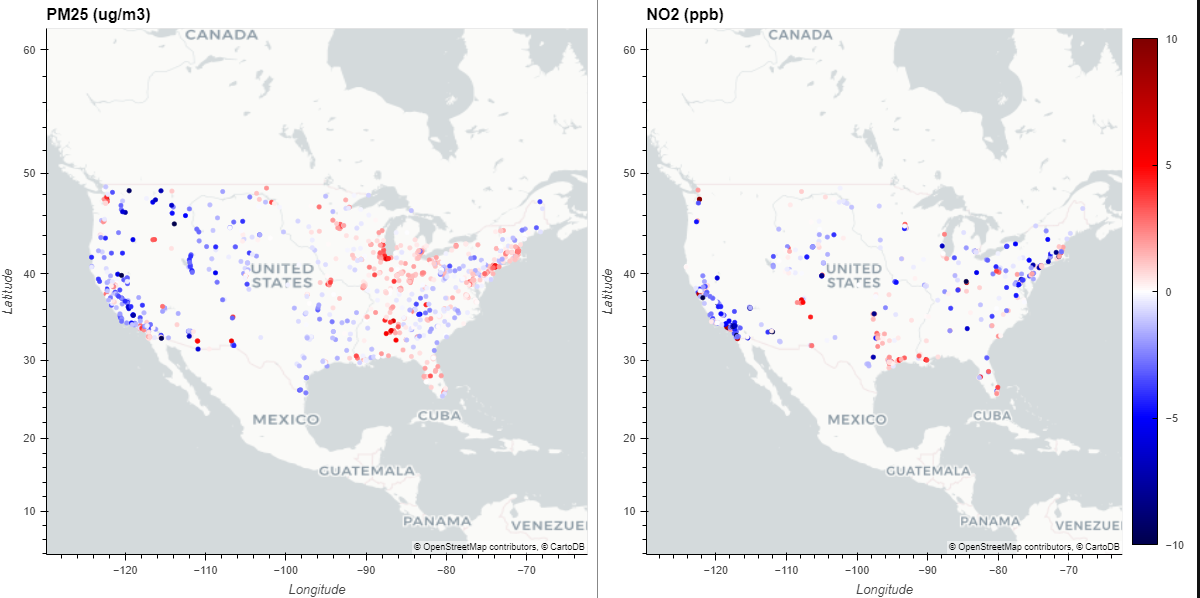


#### S4 Fig. Error (CMAQ-AQS) Spatial Maps for annual PM_2.5_ and NO_2_ for 2016.

#### RAMP Hybrid Analysis

We developed and applied a novel hybrid data fusion approach that combines a chemical transport model with a line source dispersion model intended to simulate near-road sources and correct nonlinear biases through the RAMP method. This is the first time this type of fine-scale resolution method that combines a dispersion model and CTM has been applied to a nationwide scale for PM_2.5_ and NO_2_. This Hybrid method removes the need to run multiple CTMs to obtain background emissions and includes chemical process related formation/loss from local sources that CTMs cannot simulate if emissions were zeroed out. Through this method we were able to estimate fine-scale concentrations at census block level that capture the sharp gradients near roads that are ignored when relying only on a grid-based model. We were able to rely on this Hybrid model given that we correct biases using the RAMP method. This approach can account for the nonhomogeneous, nonlinear biases of model performance, and allows us to adjust predictions differently near and far away from the road sources while at the same time keeping within-cell variability to at least 200 m grid resolution. Additionally, the RAMP Hybrid approach achieved significant model performance improvement when compared to the CMAQ and Hybrid. With a R^2^ of 0.51 for PM_2.5_ and 0.81 for NO_2_, the RAMP hybrid method improved R^2^ by ~0.2 for both pollutants (an increase of up to ~70% for PM_2.5_ and ~31% NO_2_). Additionally, RMSE for RAMP Hybrid was 1.45 μg/m^3^ and 2.51 ppb for NO_2,_ decreasing (i.e., improving) from just CMAQ and Hybrid by up to ~37% and ~53%, respectively.


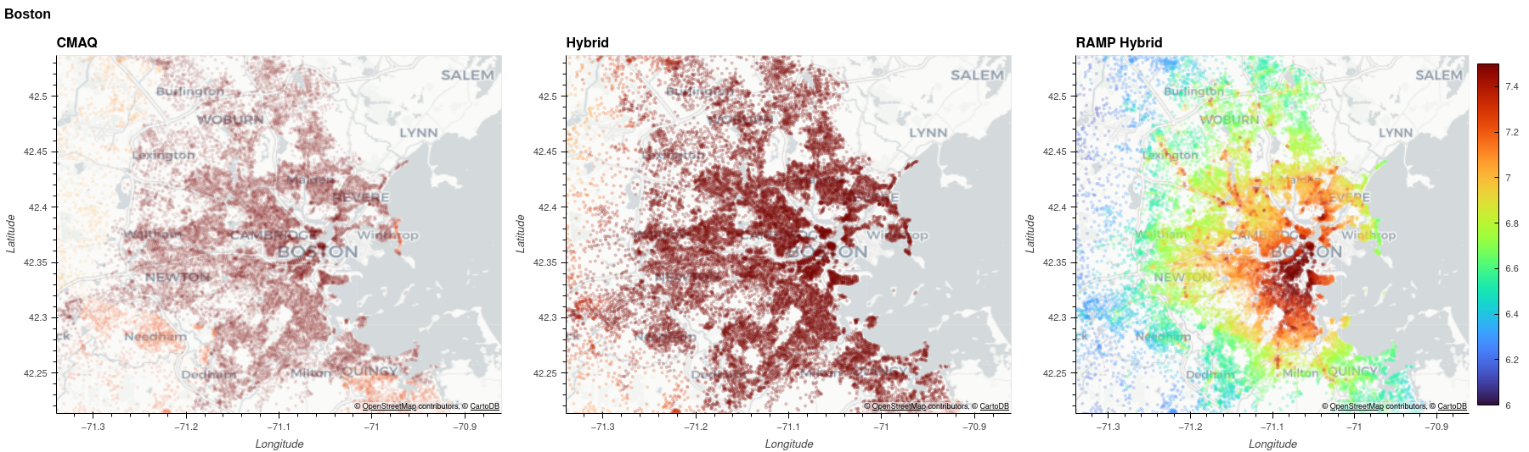


#### S5 Fig Spatial maps of PM_2.5_ (μg/m^3^) in Boston, MA with a scale that highlights near-road gradients.


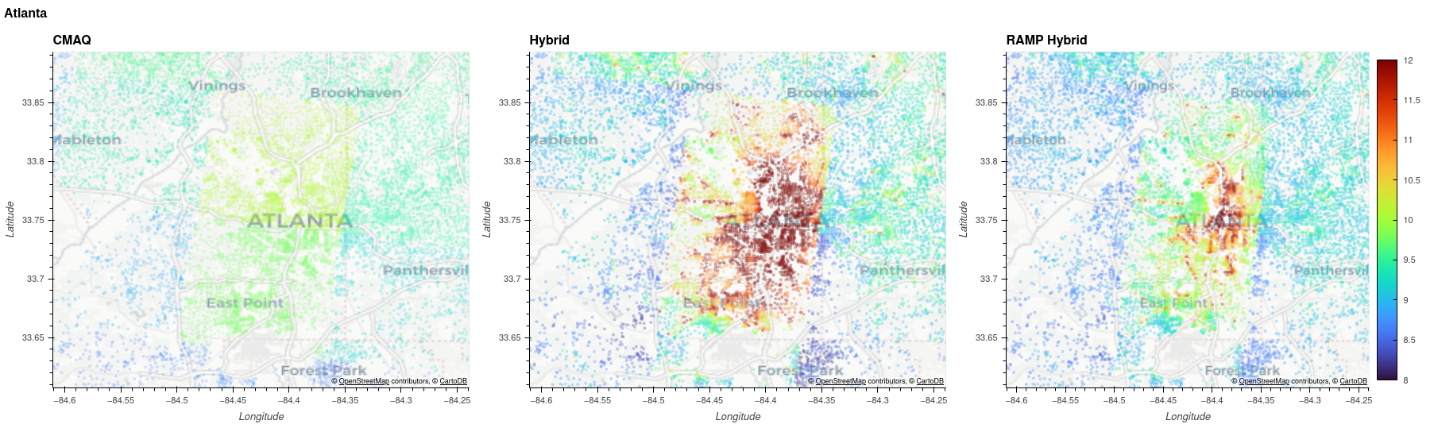

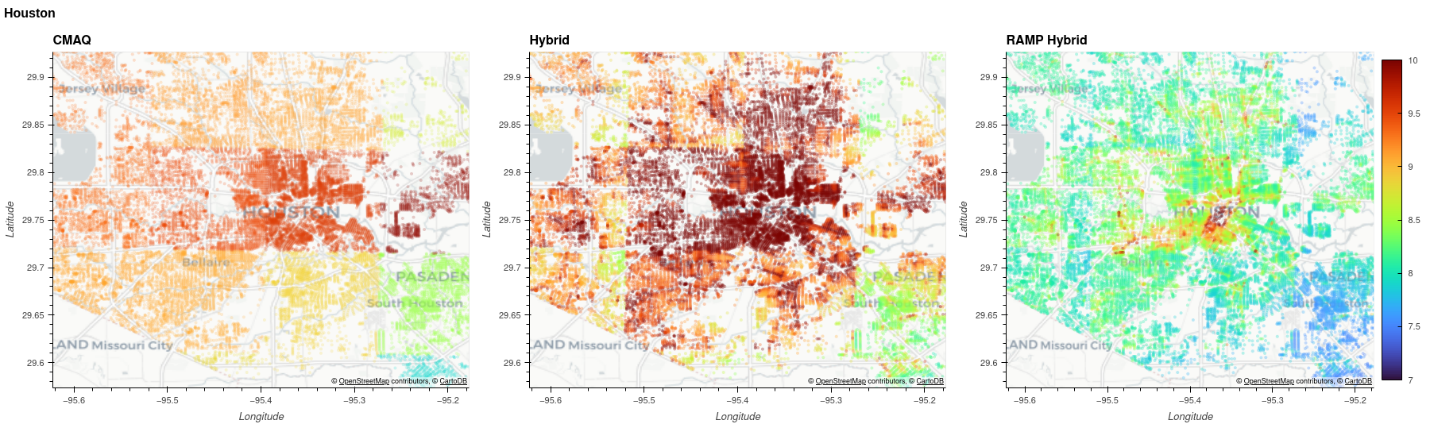

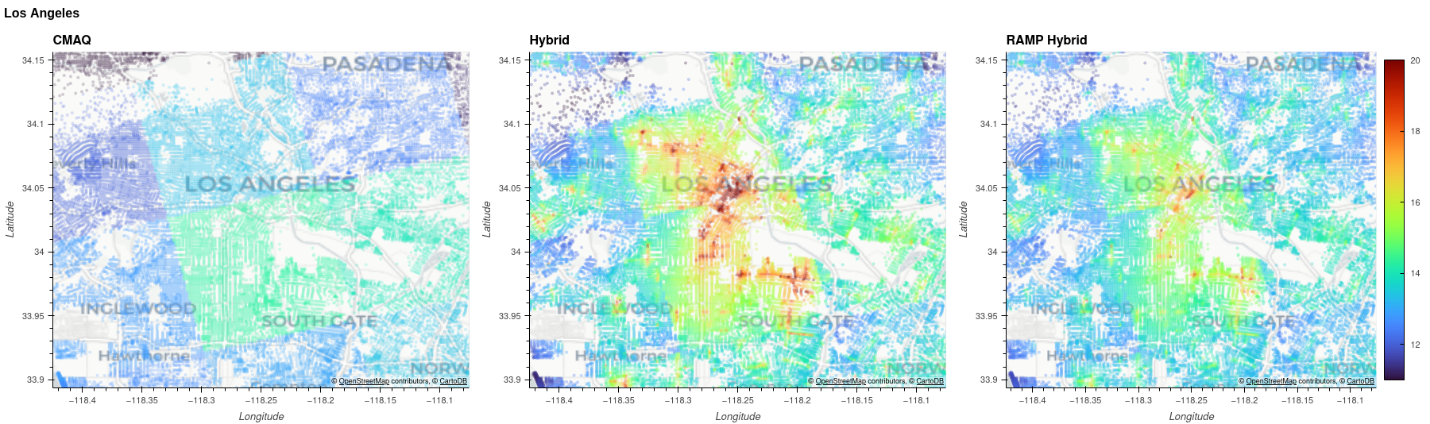

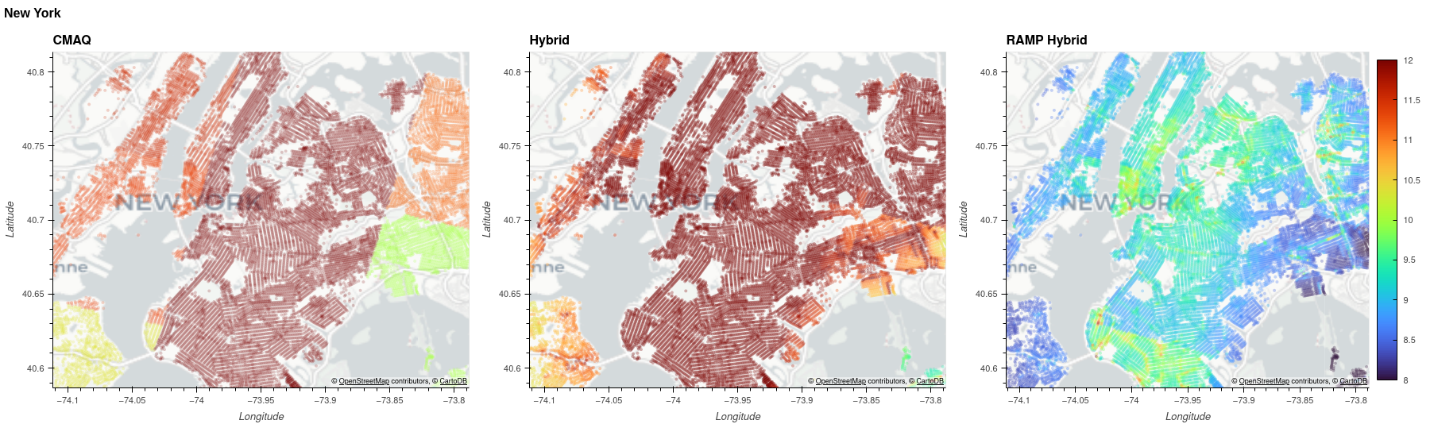

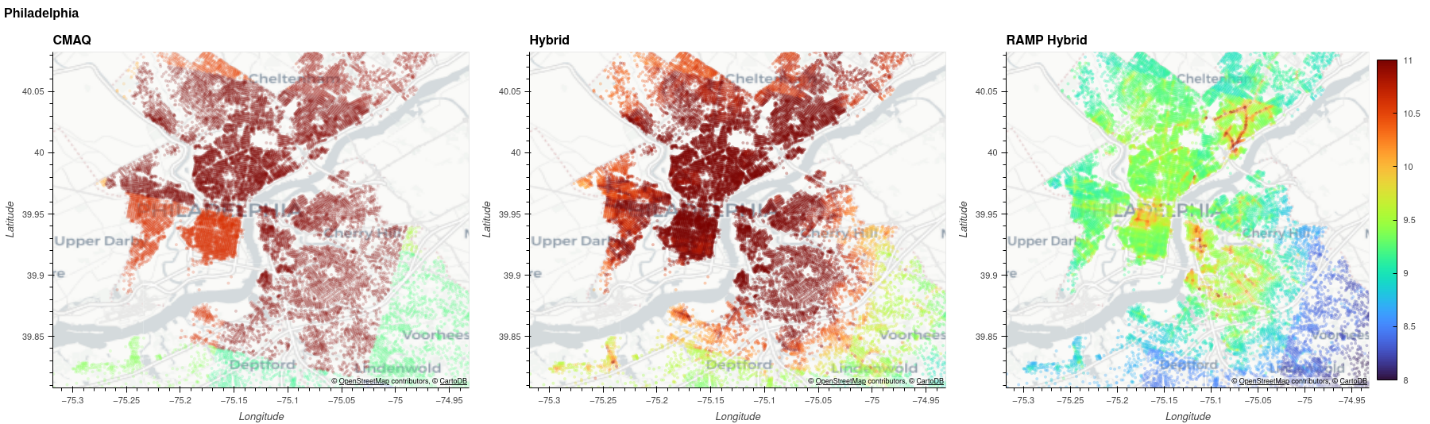

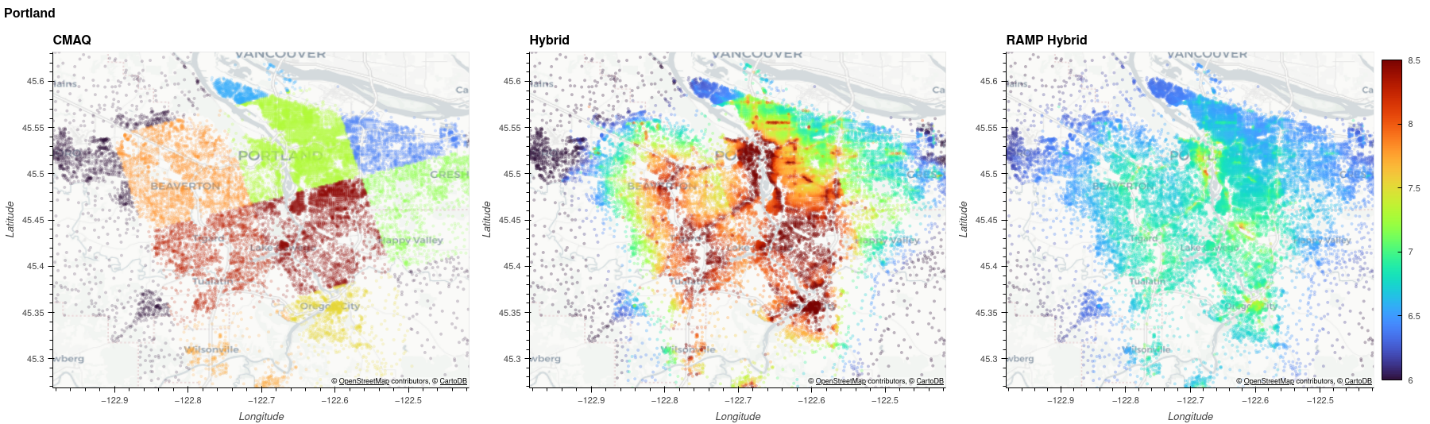

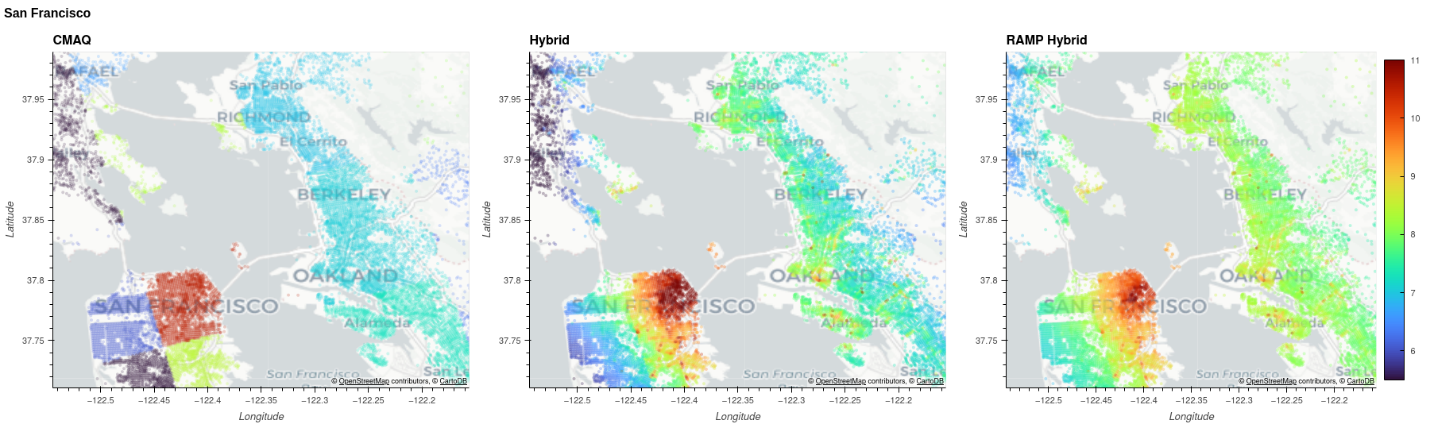

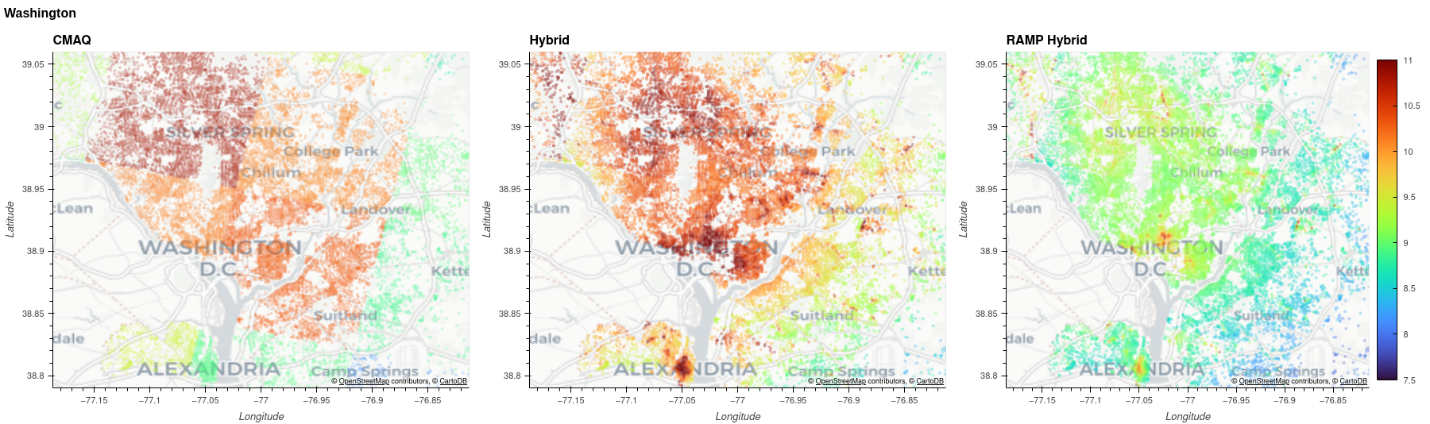


#### S6 Fig. Spatial maps of PM_2.5_ (μg/m^3^) in various high density metropolitan areas.


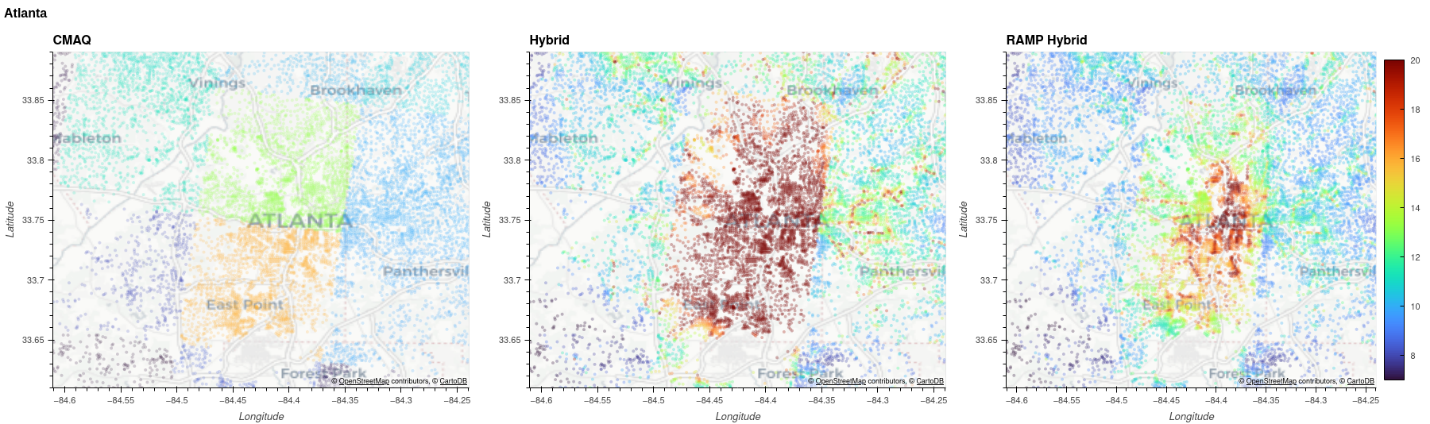

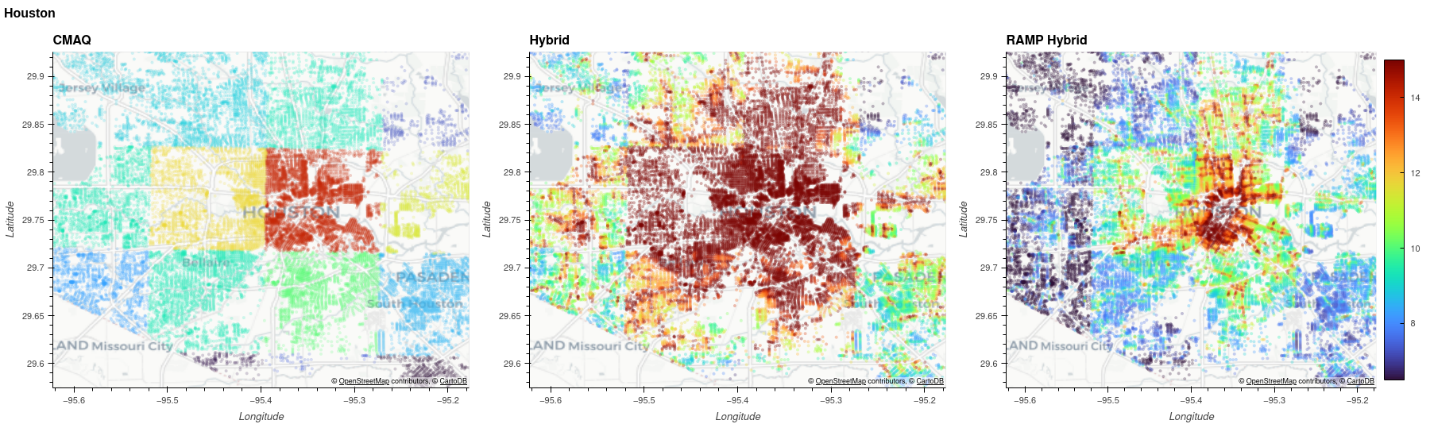

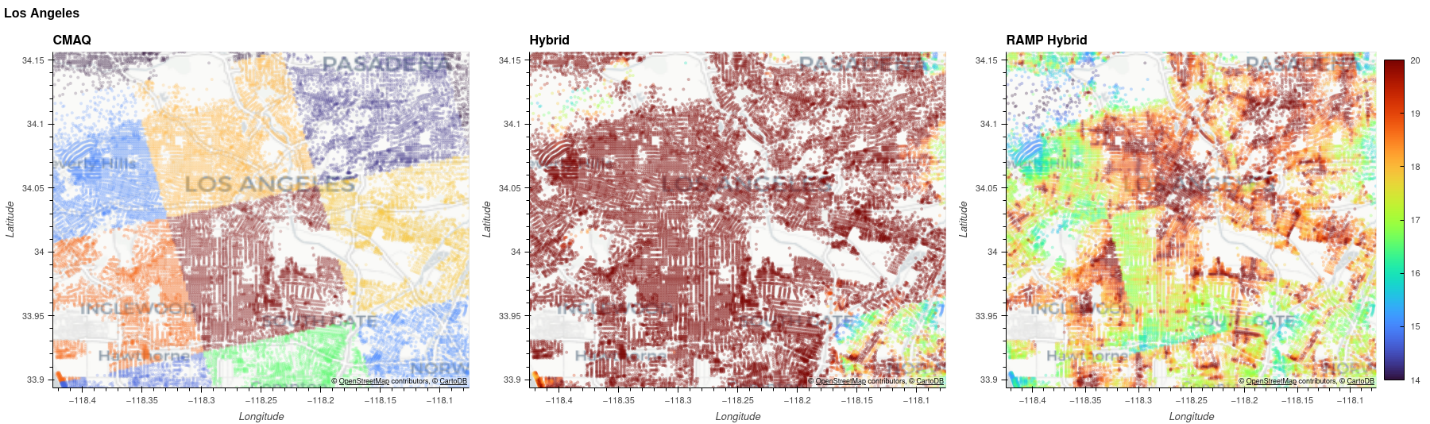

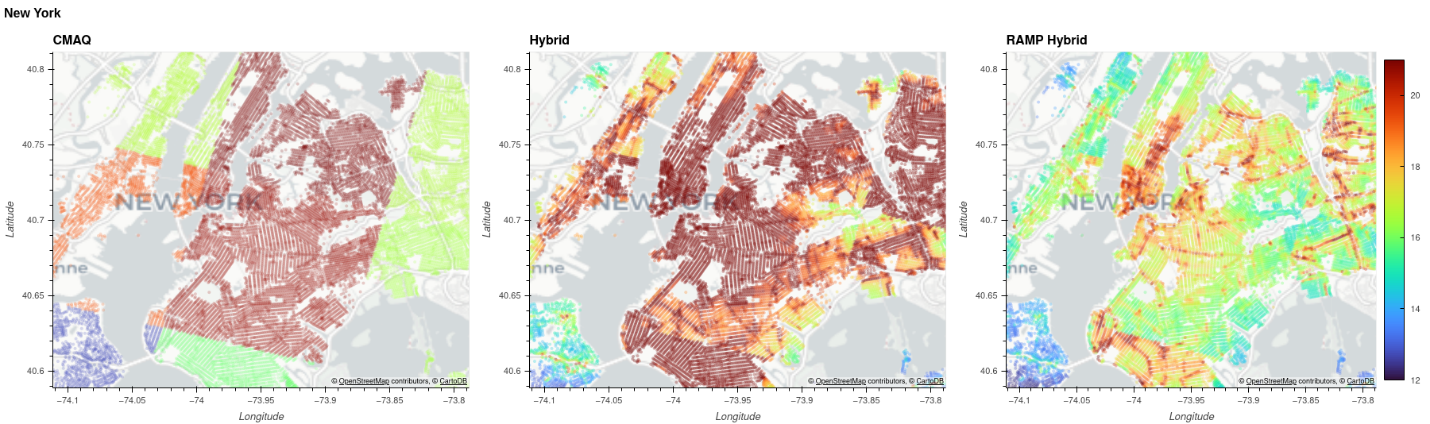

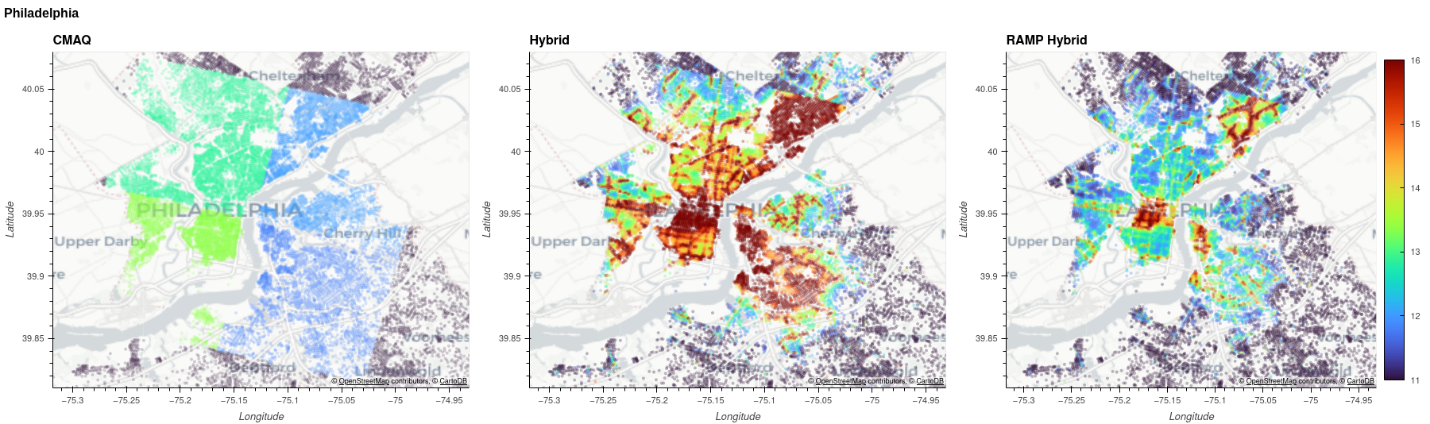

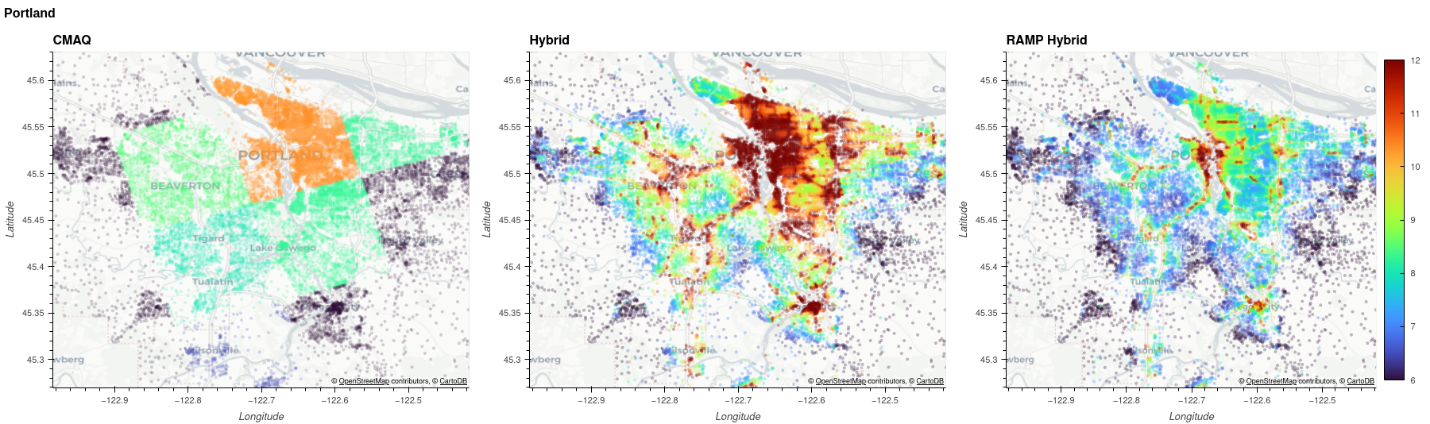

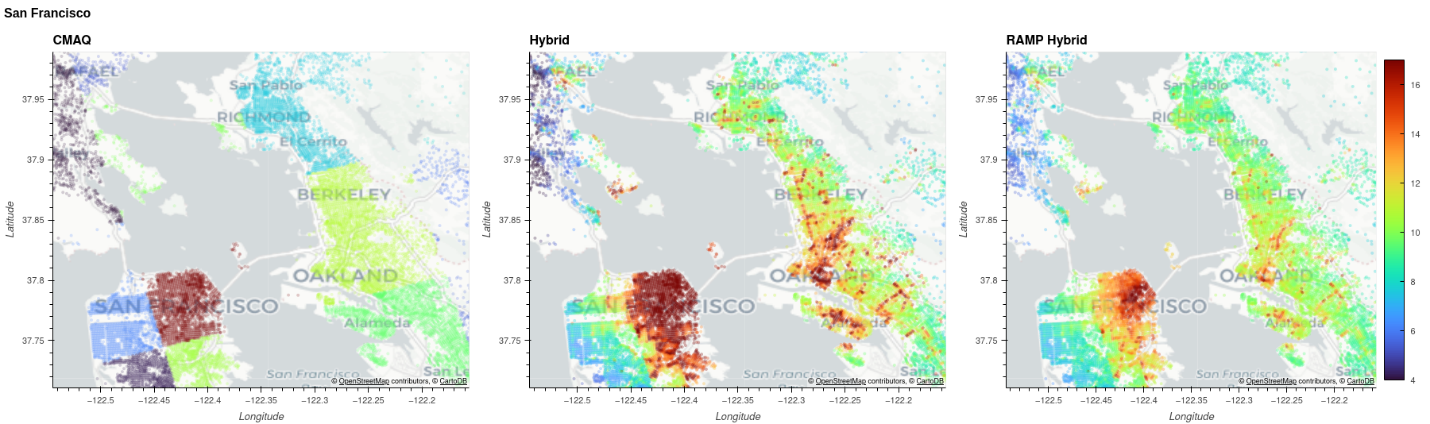

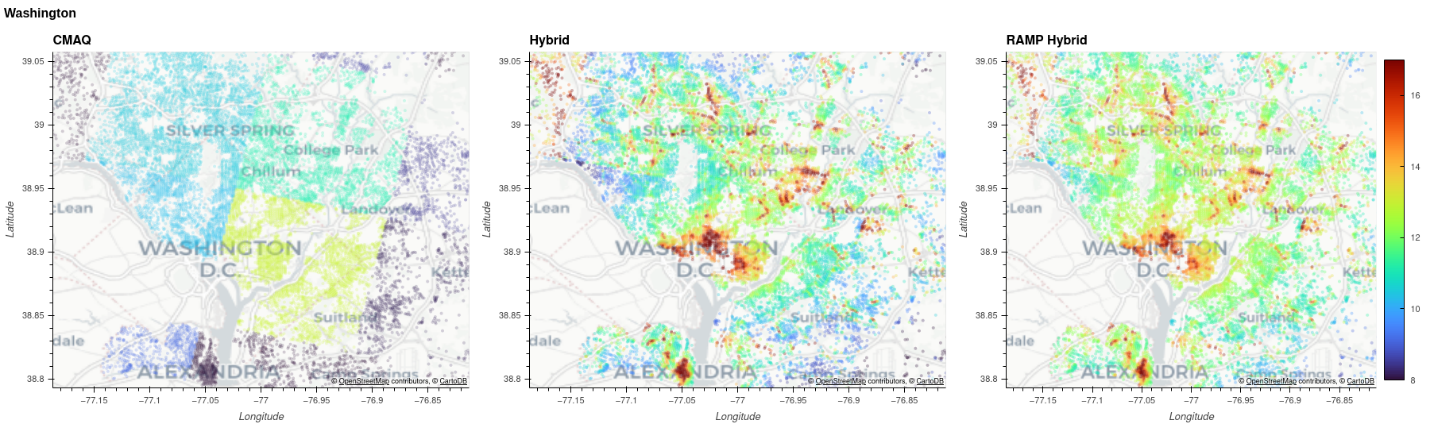


#### S7 Fig. Spatial maps of NO_2_ (ppb) in various high density metropolitan areas.


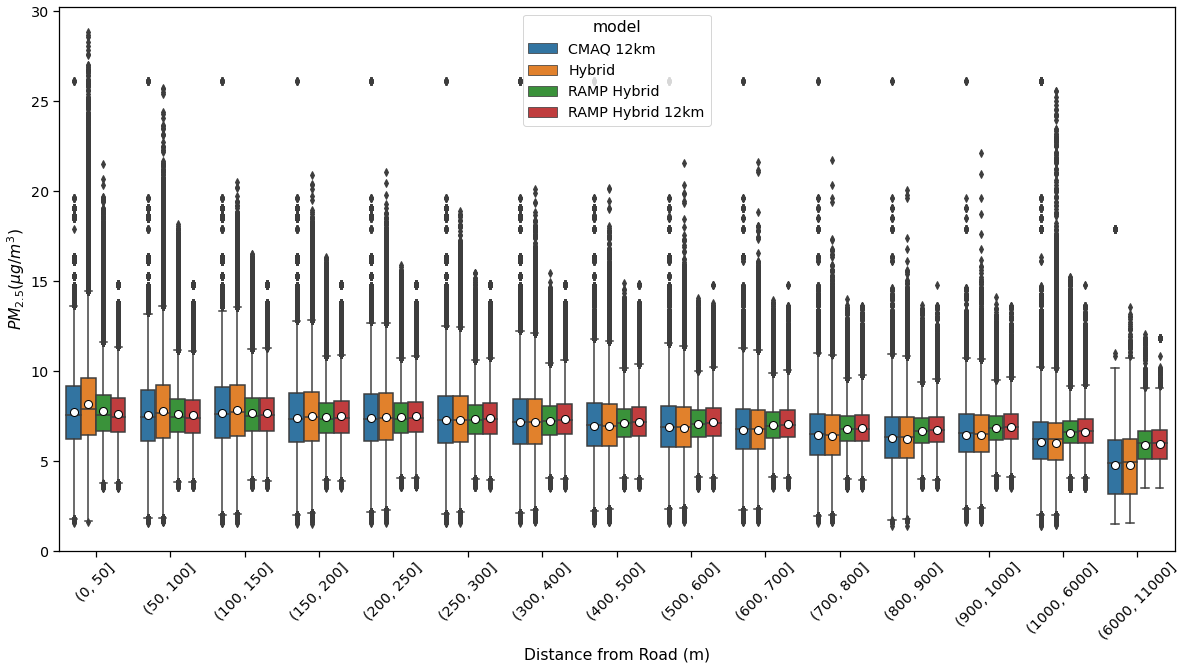


#### S8 Fig. Annual concentration of PM_2.5_ (μg/m^3^) vs binned distance from road (m). The box represents the middle 50% of the data, extending from the 25^th^ to the 75^th^ percentiles; the horizontal line through the center of the box is the median; the whiskers represent 1.5*IQR (the inter-quartile range is the range from the 25^th^ to 75^th^ percentiles); the points are outliers above and below 1.5*IQR.


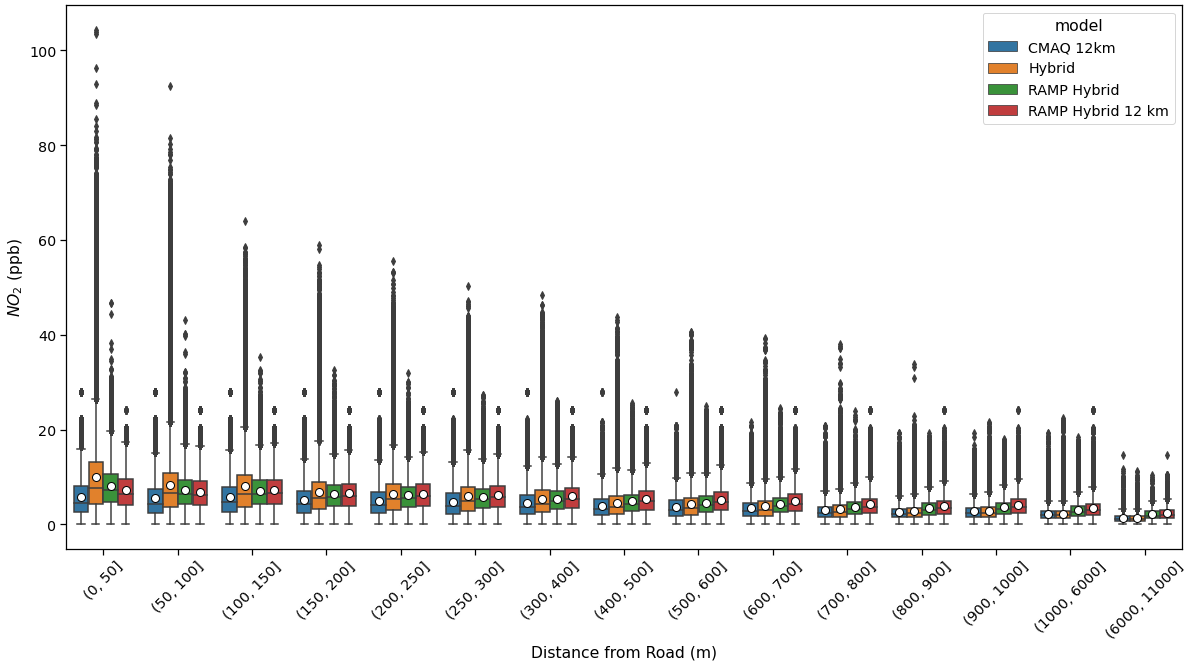


#### S9 Fig. Annual concentration of NO_2_ (ppb) vs binned distance from road (m). The box represents the middle 50% of the data, extending from the 25^th^ to the 75^th^ percentiles; the horizontal line through the center of the box is the median; the whiskers represent 1.5*IQR (the inter-quartile range is the range from the 25^th^ to 75^th^ percentiles); the points are outliers above and below 1.5*IQR.

# Health Risk Assessment

####
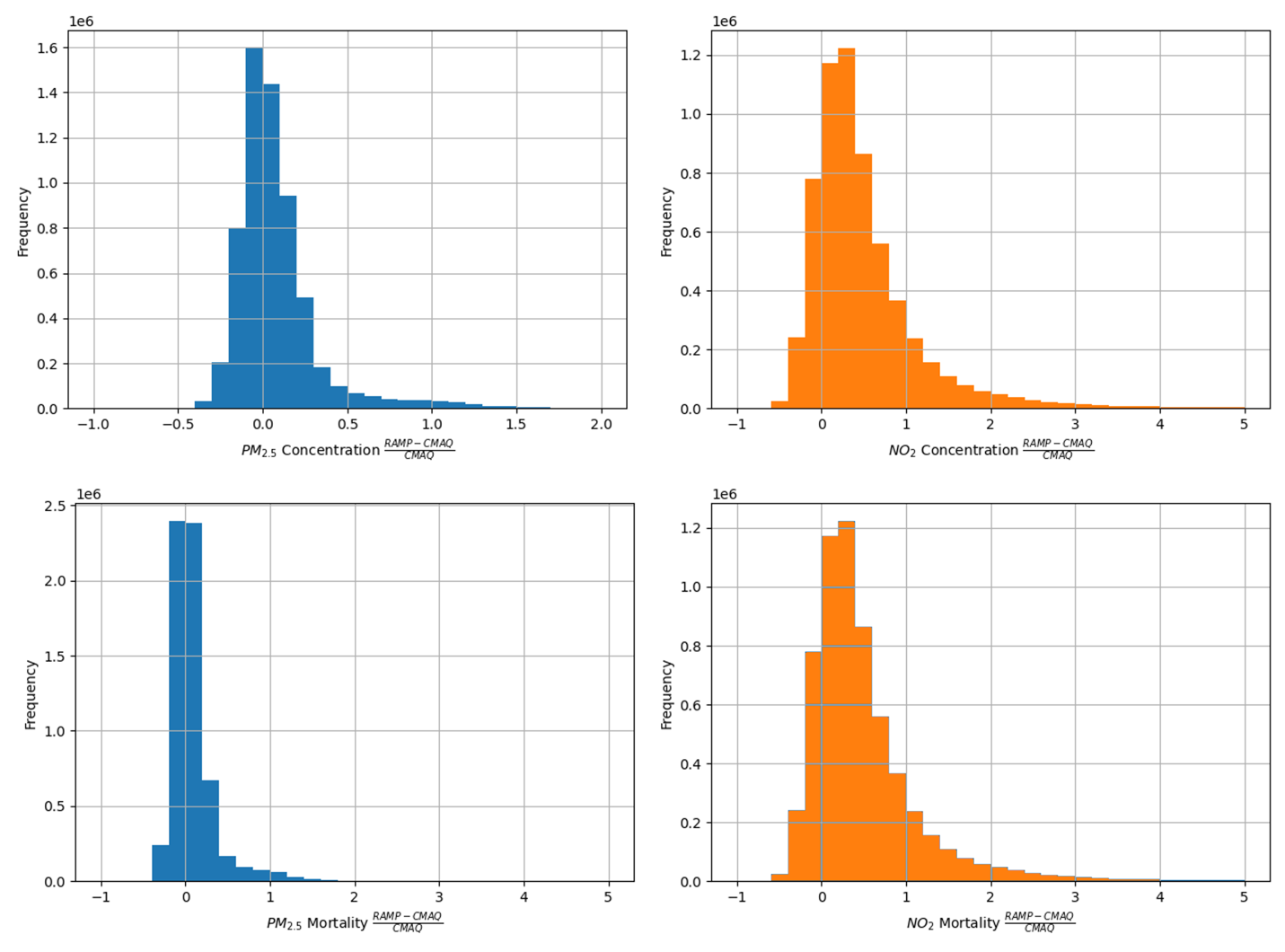
 S10 Fig. Histogram of percent change for PM_2.5_ and NO_2_ concentrations and premature mortality.

Table S1 shows the sum of differences in premature mortalities attributable to PM_2.5_ and NO_2_ between RAMP Hybrid and CMAQ across the continental U.S. Additionally, the red highlighting in Table S1 represents the sum of excess premature deaths estimated by RAMP Hybrid vs. CMAQ 12 km (12 km x 12 km). The blue highlighting represents the sum of the excess premature deaths estimated by either CMAQ 12 km or RAMP Hybrid 12 km when compared to RAMP Hybrid across the U.S.

#### Table S1. Differences in premature mortality attributable to PM_2.5_ and NO_2_ between RAMP Hybrid vs. CMAQ and RAMP Hybrid vs. RAMP Hybrid 12km aggregated to any distance from a major road and to less than 125 m from a major road. The red highlighting represents the sum of excess premature deaths estimated by RAMP Hybrid vs. either CMAQ 12 km (12 km x 12 km) or RAMP Hybrid 12 km. The blue highlighting represents the sum of the excess premature deaths estimated by either CMAQ 12 km or RAMP Hybrid 12 km when compared to RAMP Hybrid. The numbers not highlighted are the net sum of the corresponding red and blue. The numbers in brackets correspond to the 95% confidence intervals.

|  |  | All Census Blocks across U.S. | Census Blocks < 125m from Major Road |
| --- | --- | --- | --- |
| PM_2.5_ | RAMP Hybrid - CMAQ 12 km | -18,079 [-15,276 -21,023] | -7,213 [-6,095 -8,388] |
|  |  | 15,310 [12,936 17,802] | 4,269 [3,607 4,964] |
|  |  | -2,769 [-2,340 -3,221] | -2,944 [-2,488 -3,424] |
|  | RAMP Hybrid - RAMP Hybrid 12 km | -3,203 [-2,706 -3,725] | -643 [-544 -748] |
|  |  | 1,975 [1,669 2,297] | 1,250 [1,056 1,454] |
|  |  | -1,228 [-1,037 -1,428] | 607 [512 706] |
| NO_2_ | RAMP Hybrid - CMAQ 12km | -6,301 [-3,150 -9,451] | -2,498 [-1,249 -3,747] |
|  |  | 28,877 [14,438 43,315] | 10,930 [5,465 16,395] |
|  |  | 22,576 [11,288 33,864] | 8,432 [4,216 12,648] |
|  | RAMP Hybrid - RAMP Hybrid 12 km | -10,065 [-5,033 -15,098] | -1,424 [-712 -2,137] |
|  |  | 4,854 [2,427 7,281] | 3,668 [1,834 5,502] |
|  |  | -5,211 [-2,606 -7,817] | 2,244 [1,122 3,365] |

#### Table S2. Differences in premature mortality attributable to PM_2.5_ and NO_2_ between RAMP Hybrid vs. CMAQ and RAMP Hybrid vs. RAMP Hybrid 12km aggregated to less than 500 m from a major road and to less than 200 m from a major road. The red highlighting represents the sum of excess premature deaths estimated by RAMP Hybrid vs. either CMAQ 12 km (12 km x 12 km) or RAMP Hybrid 12 km. The blue highlighting represents the sum of the excess premature deaths estimated by either CMAQ 12 km or RAMP Hybrid 12 km when compared to RAMP Hybrid. The numbers not highlighted are the net sum of the corresponding red and blue.

|  |  | Census Blocks < 500 m from Major Road | Census Blocks < 200 m from Major Road |
| --- | --- | --- | --- |
| PM_2.5_ | RAMP Hybrid - CMAQ 12km | -15,687 [-13,255 -18,240] | -10,391 [-8,780 -12,082] |
|  |  | 10,853 [9,171 12,620] | 6,647 [5,617 7,729] |
|  |  | -4,834 [-4,084 -5,620] | -3,744 [-3,163 -4,353] |
|  | RAMP Hybrid - RAMP Hybrid 12km | -2,317 [-1,958 -2,695] | -1,159 [-980 -1,348] |
|  |  | 1,823 [1,540 2,119] | 1,535 [1,297 1,785] |
|  |  | -494 [-418 -576] | 376 [317 437] |
| NO_2_ | RAMP Hybrid - CMAQ 12km | -5,527 [-2,764 -8,291] | -3,638 [-1,819 -5,457] |
|  |  | 23,952 [11,976 35,927] | 16,047 [8,024 24,071] |
|  |  | 18,425 [9,212 27,636] | 12,409 [6,205 18,614] |
|  | RAMP Hybrid - RAMP Hybrid 12km | -6,576 [-3,288 -9,865] | -2,811 [-1,406 -4,217] |
|  |  | 4,765 [2,382 7,147] | 4,318 [2,159 6,477] |
|  |  | -1,811 [-906 -2,718] | 1,507 [753 2,260] |

#### Table S3. Differences in premature mortality attributable to PM_2.5_ and NO_2_ between RAMP Hybrid and RAMP Hybrid at census tract. The red highlighting represents the sum of excess premature deaths estimated by RAMP Hybrid vs. RAMP Hybrid at Census Tract. The blue highlighting represents the sum of the excess premature deaths estimated by RAMP Hybrid at Census Tract when compared to RAMP Hybrid. The numbers not highlighted are the net sum of the corresponding red and blue.

|  |  | Census Blocks < 500 m from Major Road |
| --- | --- | --- |
| PM_2.5_ | RAMP Hybrid – RAMP Hybrid Census Tract | -3,197 [-2,701 -3,717] |
|  |  | 2,080 [1,758 2,419] |
|  |  | -1,117 [-943 -1,298] |
| NO_2_ | RAMP Hybrid - RAMP Hybrid Census Tract | -10,224 [-5,112 -15,336] |
|  |  | 5,282 [2,641 7,924] |
|  |  | -4,942 [-2,471 -7,412] |

| 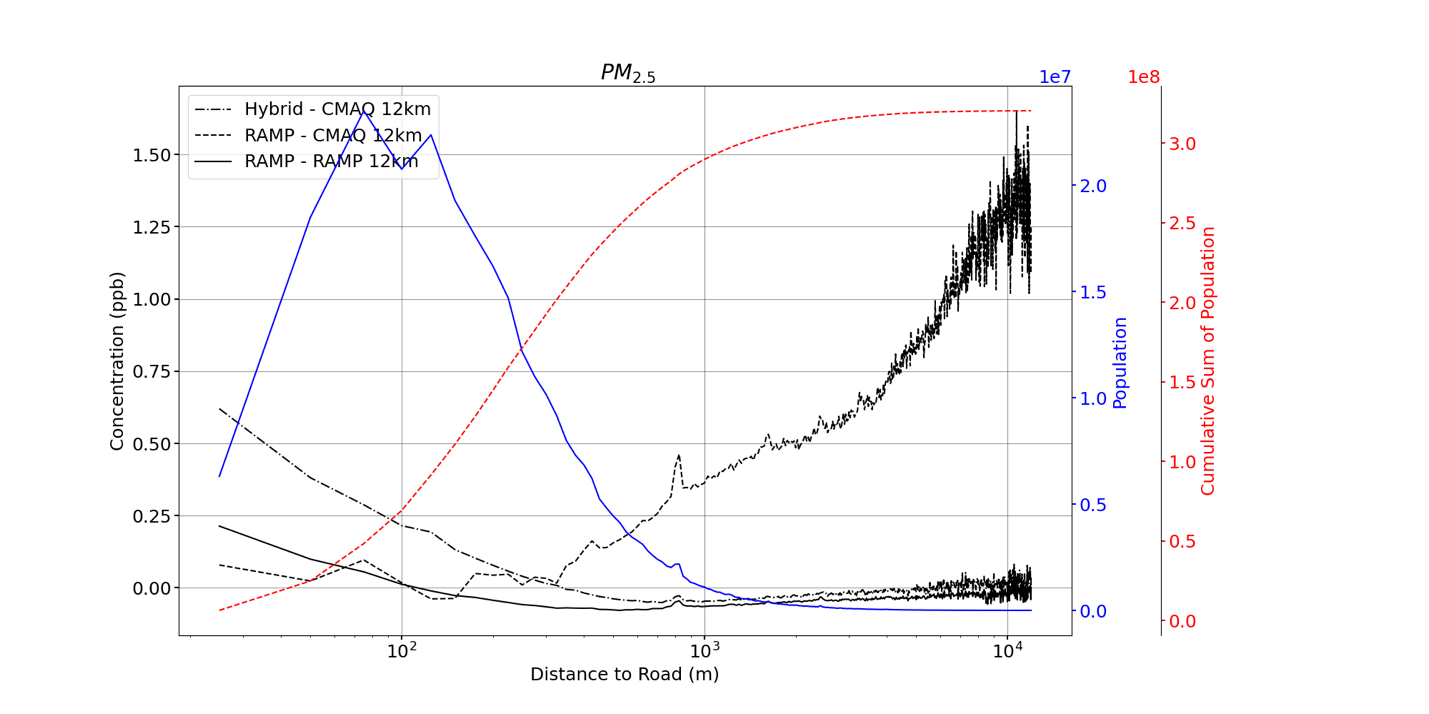 |
| --- |
| 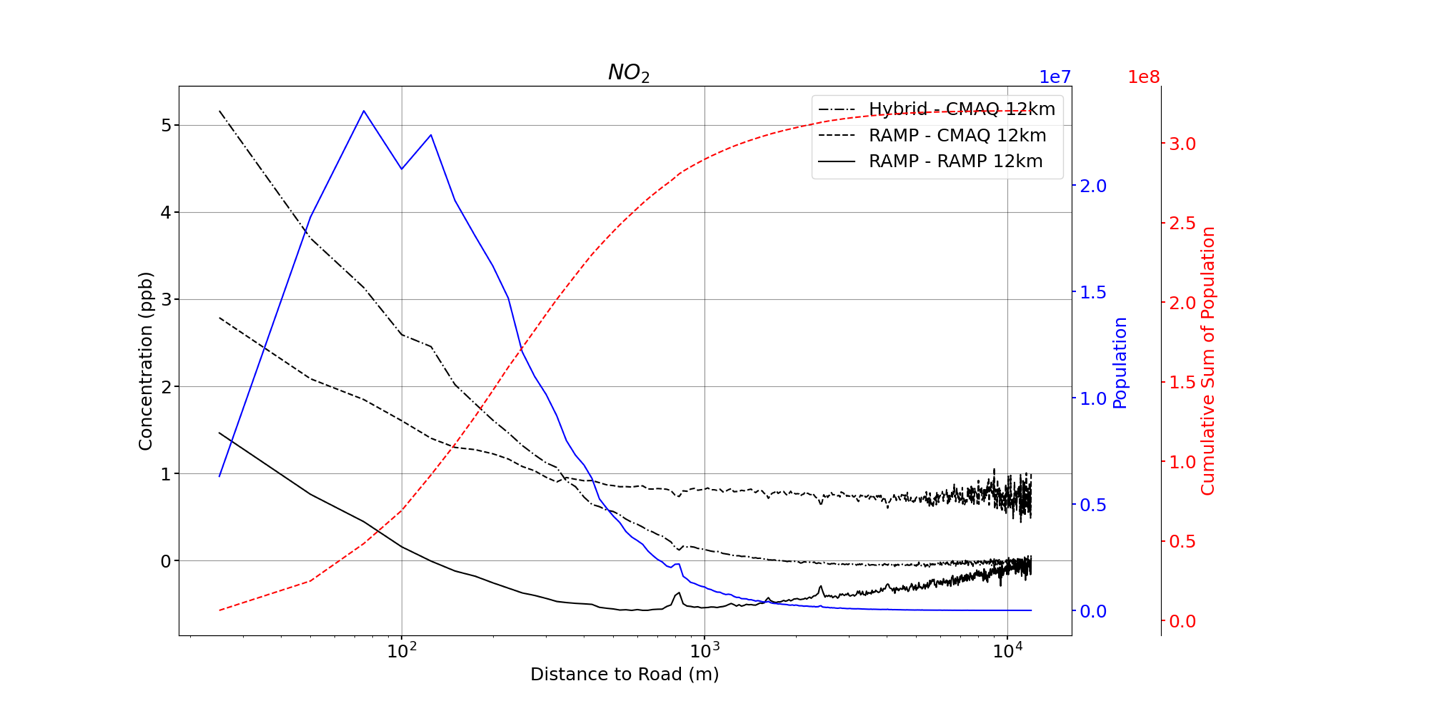 |

#### S11 Fig. Concentration difference in PM_2.5_ and NO_2_ between models with varying resolution vs distance from major road. Differences were averaged at every 25 m. The blue line represents the population aggregated at every 25 m from the road and the red line corresponds to the cumulative sum of the population.

#### Risk Assessment Comparison to Previous Hybrid Studies

Most previous studies that have implemented hybrid approaches that combine CTMs with dispersion models to increase model resolution have not performed a health impact assessment using hybrid predictions to find adverse health outcomes attributable to PM_2.5_ and NO_2_ [1–4]. Only one study that we know of considered health impacts when using these types of hybrid data fusion models [5]; we significantly improved on this study by using hourly meteorology instead of representative meteorology to obtain annual averages from the dispersion model, expanded the domain to be nationwide, and added NO_2_. We further use CMAQ at a much finer 12 km x 12 km grid resolution (instead of 36 km x 36 km); we adjusted the hybrid model to correct for nonlinear biases from the model; and finally, we consider a different CRF that accounts for all-cause mortality instead of only cardiopulmonary diseases and lung cancer. Nonetheless, the findings in the Chang et al. (2017) study suggest that there was 24% more traffic related PM_2.5_ premature mortality when using hybrid (295 [170 420] premature mortalities) vs a 36 km x 36 km CTM (237 [136 337] premature mortalities) in Central North Carolina for 2010. This difference is attributed to capturing primary on-road PM_2.5_ related near-road gradients where the hybrid approach predicted 2.5 times more primary on-road PM_2.5_ -related premature mortality than CMAQ. Primary PM_2.5_ is calculated through R-LINE which suffers from high biases under low dispersion conditions (i.e., low wind speeds or calm conditions) [6]. According to the Zhai et al. study, uncorrected R-LINE estimates increase annual PM_2.5_ concentrations by ~25%–30%. Another factor that would affect the estimates of on-road primary contributions are the emissions. According to the national emissions inventory (NEI), primary on-road emissions have been steadily decreasing from 252,603 tons/year in 2008 to 197,527 tons/year in 2011 to 114,069 tons/year in 2017 [7]. Additionally, the percentage of on-road primary PM_2.5_ to total primary PM_2.5_ emissions has also been decreasing from 0.042% in 2008 to 0.032% in 2011 to 0.020% in 2017 [7]. Thus, both these factors (R-LINE biases and emissions differences) account for larger on-road primary PM_2.5_ estimates in the Chang et al. study (2017) vs our study.

For our study, considering all-cause mortality (not just cardiopulmonary disease and lung cancer for all ambient PM_2.5_ (not just traffic-related), we estimate 4,679 [3,953 5,441] premature mortalities using RAMP Hybrid and 4,418 [3,733 5,137] premature mortalities using CMAQ for the same counties in Central North Carolina as used in the Chang et al.’s (2017) study for 2016. This amounts to a ~5% increase in premature mortality when using RAMP Hybrid rather than CMAQ. However, as mentioned before, this comparison (CMAQ vs RAMP Hybrid) considers two effects, different resolutions with each model, and the biases of two different models. In the same way, the Chang et al. study also considers these two effects when they compare CMAQ against their hybrid approach. These two distinct models also have different biases that are combined into their comparison (where R-LINE is biased high and CMAQ is biased low near roads). Thus, these types of comparison do not reflect the effect of resolution alone on premature mortality, as we have done when we compare the coarse-scale RAMP hybrid vs the fine-scale RAMP hybrid. As mentioned previously, the Punger and West [8] study emphasized the importance of comparing like models to obtain the effect of grid resolution. As aforementioned, this study used the same CTM (CMAQ) and compared results from a 36 km x 36 km grid resolution run with those of a 12 km x 12 km grid resolution and found higher estimates of premature mortality in the coarse model. This comparison considers two changes, model resolution and the effect of the model’s atmospheric processes. However, when they averaged modeled concentrations at 12 km x 12 km grid resolution to coarser resolutions (to only consider the effect of resolution), higher estimates of premature mortality were estimated at the fine-scale model. It is important to mention that this study does not analyze the effect of resolution at grid sizes under 12 km x 12 km, as we do in our study.

# Environmental Justice Assessment

#### Table S4. Population Weighted Exposure Across the US for PM_2.5_ and NO_2_.


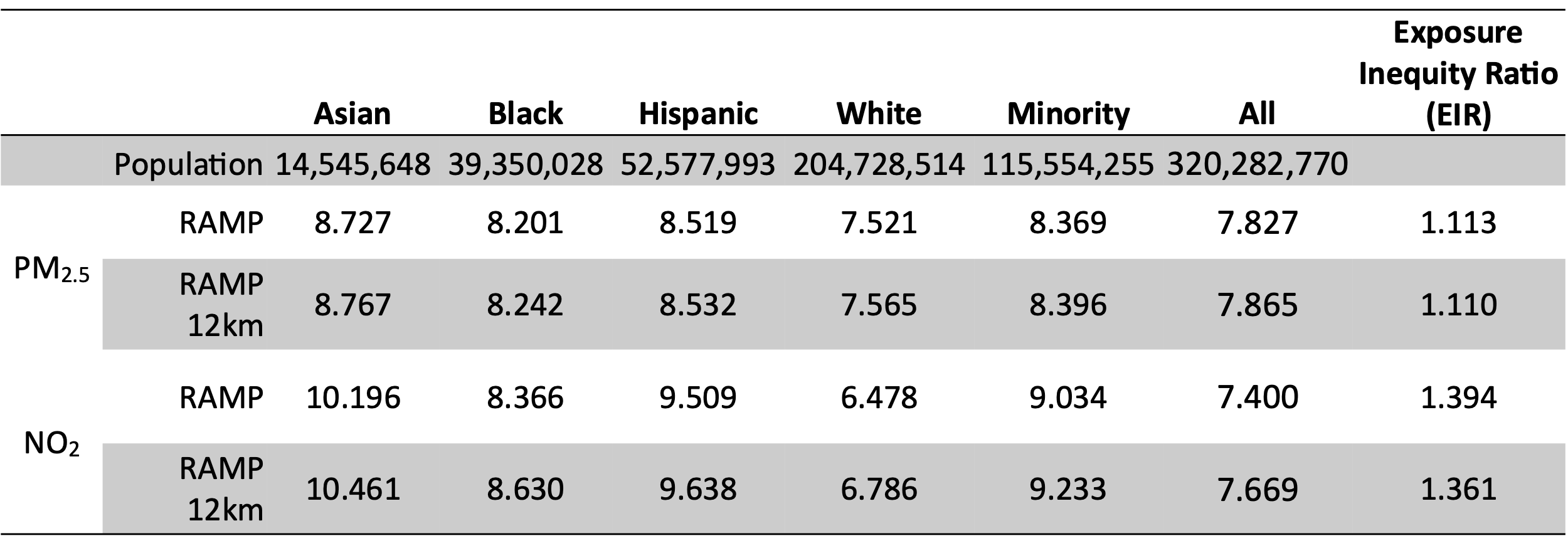


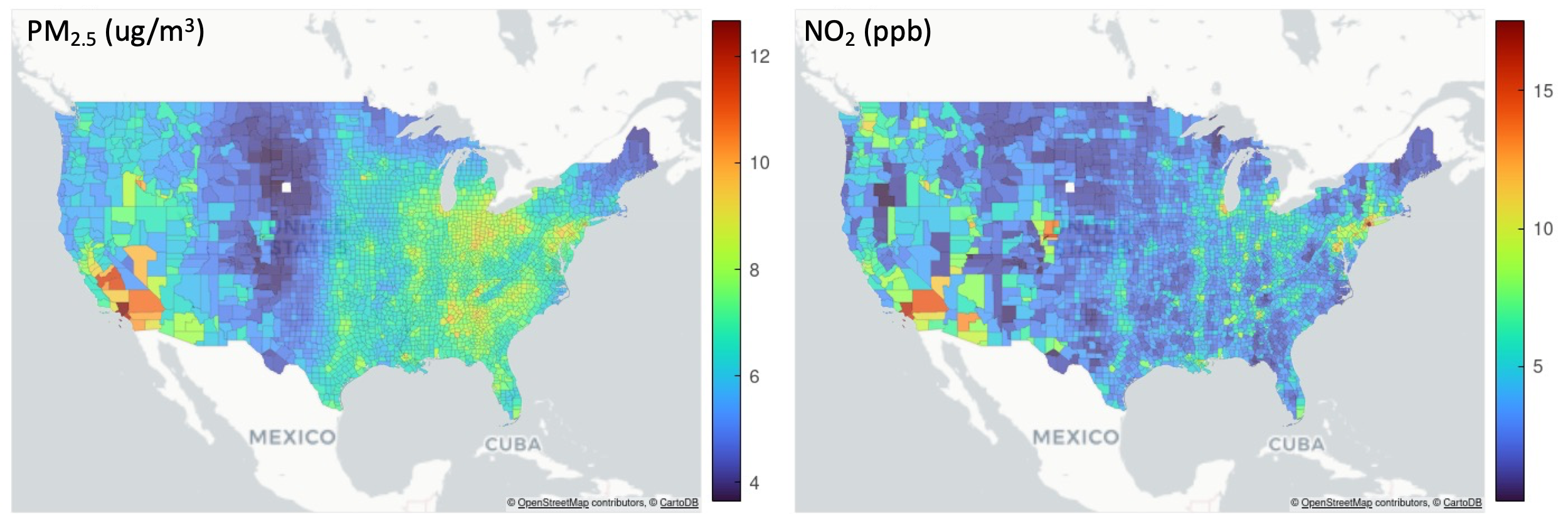


#### S12 Fig. Population weighted exposure aggregated at county level for PM_2.5_ (left) and NO_2_ (right).


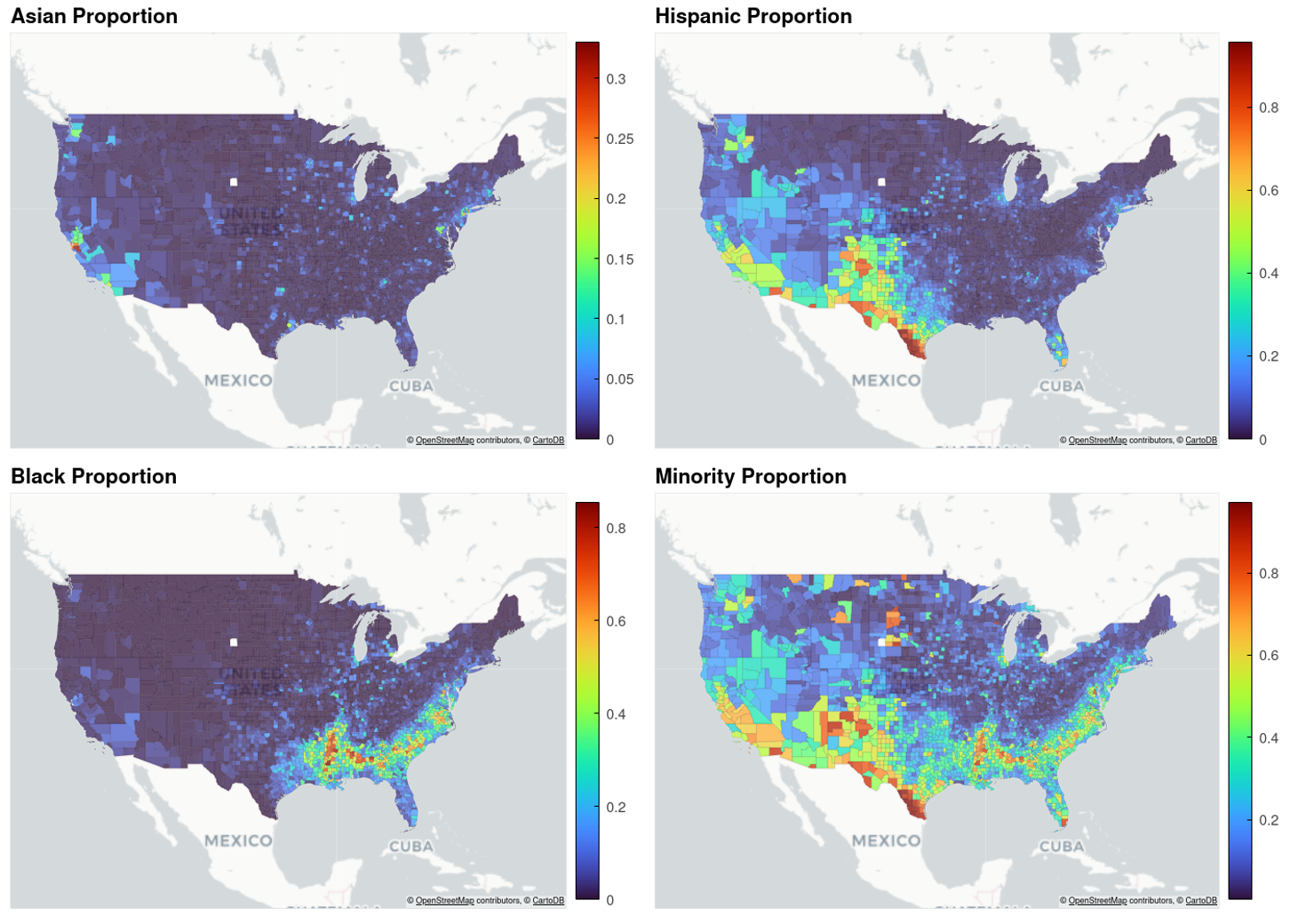


#### S13 Fig. Proportion of Minority Population across the US: Asian Population (top left), Hispanic Population (top right), Black Population (bottom left), and All Minorities (bottom right).


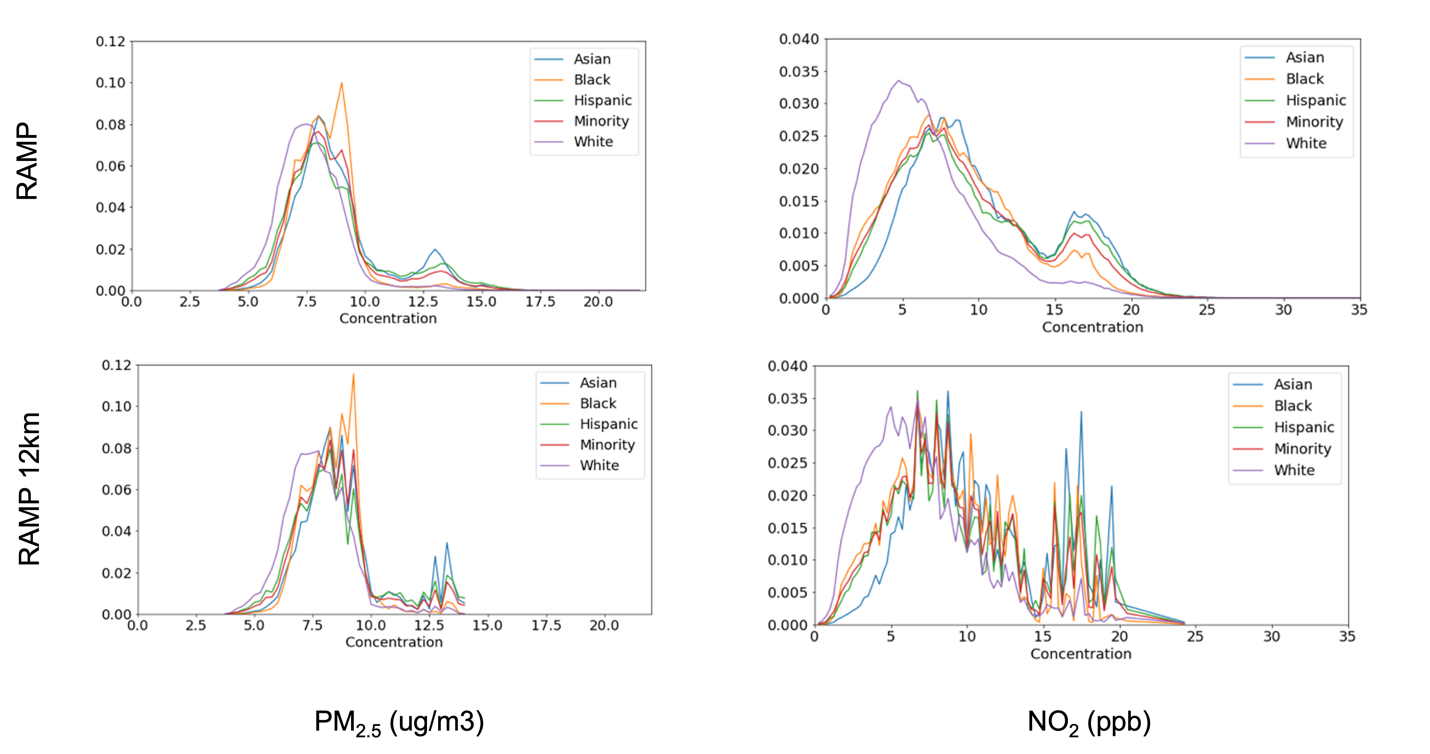


#### S14 Fig. Proportion of the population exposed to varying levels of PM_2.5_ (left) and NO_2_ (right) for RAMP (bottom) and RAMP 12km (bottom).

#### Nationwide bimodal exposure disparity

There is a bimodal effect (for PM_2.5_ and for NO_2_) that causes exposure disparity between the Minority and White population. Thus, we created histograms of concentrations (Figure S14) that show the distribution of concentrations that each population is exposed to. The distribution of these concentrations is bimodal, with both PM_2.5_ and NO_2_ having a high concentration mode, and a low concentration mode. These two modes result in two distinct effects on the exposure inequity between Minority and White populations.

Let us focus first on the inequity effect of the high concentration mode. Focusing on the mode of high concentrations between 12-15 µg/m^3^ for PM_2.5_, we see from the concentration histogram plots Figure S14 that a significant proportion of the Asian (blue line) and Hispanic (green line) population are exposed to these high concentrations, whereas the White population (purple line) and the Black population (orange line) are hardly exposed to these levels of PM_2.5_. As mentioned earlier, the high PM_2.5_ concentrations associated with the high concentration mode occurs in an area mostly located in California, and we can see from the population map Figure S14 that the proportion of Latino and Asian are significantly larger than that of the White population in that area, resulting in a Minority exposure larger than that of the White population. The Black population is not contributing to this mode since there is not a high proportion of the Black population in the area with high PM_2.5_ in California. When focusing on NO_2_, we see that the mode of high concentration ranges between 15-20 ppb Figure S14 corresponding to elevated NO_2_ not only in California (an area with high proportion of Asian and Hispanic population) but also New York (an area with a high proportion of the Black population). As a result, similarly to PM_2.5_, a high proportion of Minority is exposed to the high mode NO_2_ concentration, while that is not the case for the White population.

Let us now turn to the effect of the low concentration mode on exposure inequity. The low concentrations associated with the low concentration mode occurs in an area that comprises most of the U.S. and is not localized as was the case for the high concentration mode. Looking at the concentration histogram Figure S14 we see that for both PM_2.5_ and NO_2_, the low concentration mode shift from the left to the right as we go from White to Minority populations. This means that for most of the U.S., a second effect takes place, which is that Minorities are exposed to higher levels of concentrations of pollutions compared to their White counterparts, or in other words, that the White population concentration crest at lower concentrations than any of the Minorities or all the Minorities combined. This effect on health inequity is not identified by just looking at a nationwide map of concentration, therefore this effect on exposure inequity must occur at a fine spatial scale. In other words, the residence locations of Minority are somehow near that of the White population but with higher concentrations.

The output of the RAMP model is unique, because not only can it quantify exposure inequity nationwide, but also helps us understand exposure inequity at a finer scale within this low concentration mode. One clear indication of how lower resolution results can obfuscate the EIR is how noisy the RAMP 12 km results are, as shown in Figure S14. This noise will only be amplified when focusing on finer geographical regions.


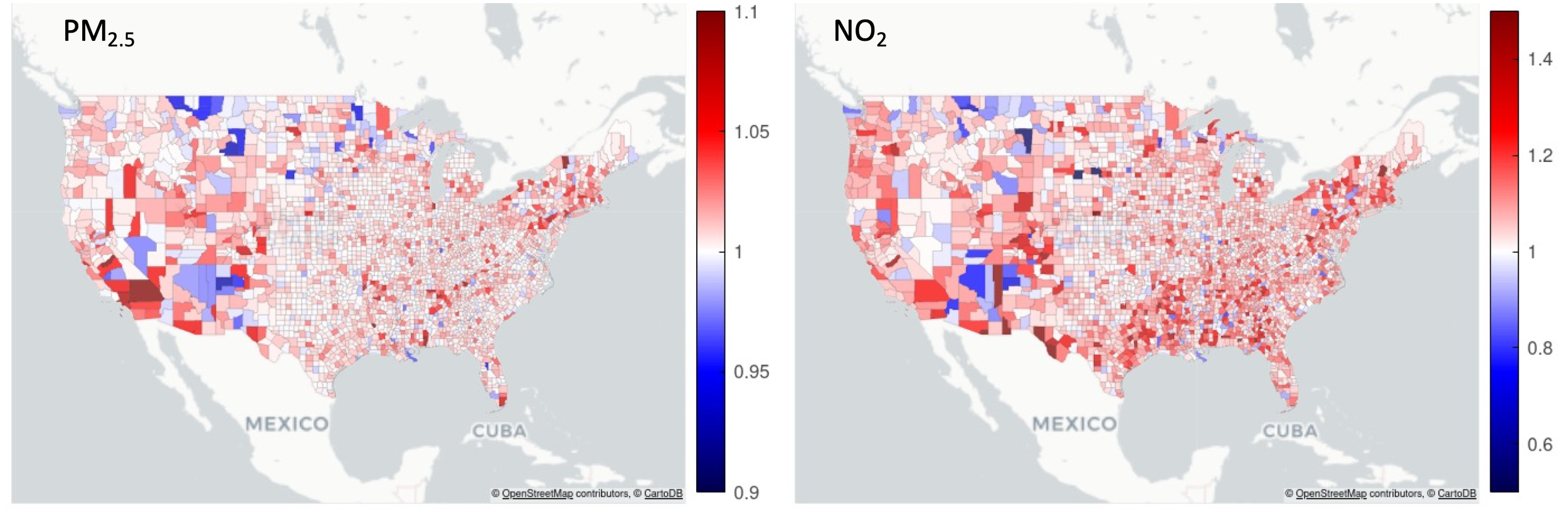


#### S15 Fig. Exposure Inequity ratio across the U.S. at each county for PM_2.5_ (left) and NO_2_ (right).

To test whether there is disparity at a finer scale, we scale down the exposure inequity analysis from nationwide to county level. We see that inequity persist in some counties as shown in Figure S15. This figure shows the EIR in each county in the US for both PM_2.5_ and NO_2_. Most of the counties show an EIR close to 1, but there are counties that have EIRs of up to 1.2 for PM_2.5_ and 1.6 for NO_2_. These counties have EIRs that are as high or higher than what we calculated for the nationwide EIR. Thus, this map provides a useful tool that shows widespread exposure disparity across the U.S, and it is the first time that inequity has been estimated using a hybrid air pollution dispersion model that accounts for bias-correction.

Zeroing into the effect of exposure inequity in a particular county, one can focus on counties with high population weighted exposure and high EIR. San Bernardino, CA is a county with a high population weighted exposure and with a population of about 2.1 million people. For PM_2.5_, this county shows the second highest EIR of counties ranked by EIR with population greater than 100,000 people (see Table S6 which lists the top 10 counties by EIR). Using the RAMP Hybrid model, we can explore the effect of EIR at a scale as fine as census tract and census block group. Figure S16 shows how EIR changes across San Bernardino at these various political/statistical geographic levels. At the county level this county has a high EIR of about 1.11 for PM_2.5_ and about 1.26 for NO_2_. Exposure inequity (EIR > 1) can also be seen at the census tract and census block group level within this county. Note that the EIR value changes from greater than 1 at the county level to either greater or lower than 1 in the finer geographic unit aggregation. This means that whether an area is inequitable or not (EIR > 1 vs EIR < 1) is a function of the aggregation scale (similarly depicted in S15 at nationwide scale). This type of analysis allows to pinpoint where the inequity is occurring within the county. It also allows to explore how exposure inequity manifests at different scales.

Focusing on the southwest portion of the county between Pasadena and Palm springs, Figure S17 shows the RAMP hybrid concentrations alongside the percent Minority at the census block level for both PM_2.5_ and NO_2_. These maps show that there exist localized areas where the exposure inequity lines up with strong proportion of Minority at a fine spatial scale. This causes localized high EIRs. These localized high EIRs (i.e., EIR over small distances) are revealed through the fine-scale granularity of the RAMP hybrid model.


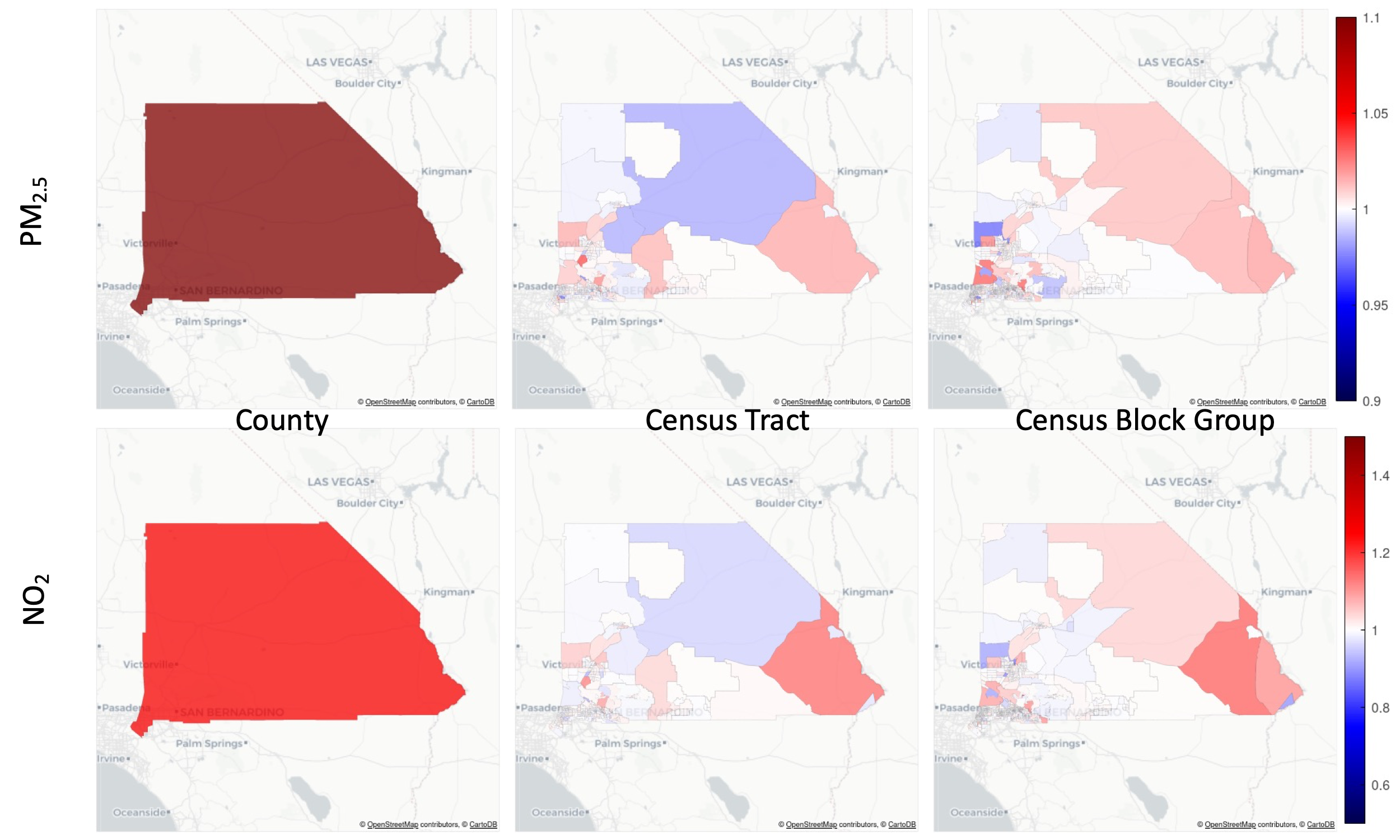


#### S16 Fig. Exposure Inequity Ratio (EIR) in San Bernardino County, CA for PM_2.5_ and NO_2_ at County (left), Census Tract (middle), and Census Block Group level (right).


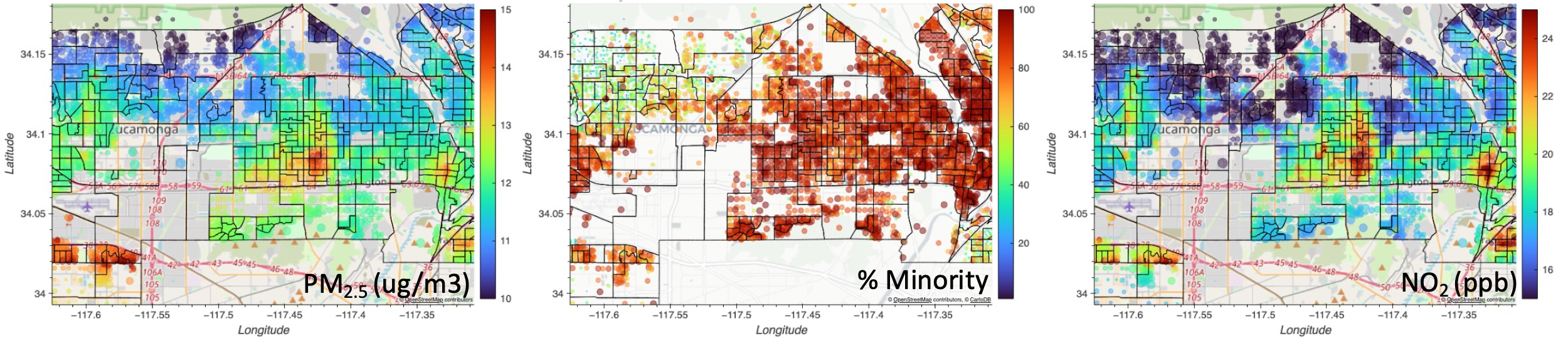


#### S17 Fig. RAMP Hybrid concentration for PM_2.5_ (left) and NO_2_ (right) at census block centroids at Rancho Cucamonga, Fontana and San Bernardino, CA. The size of the census block centroid is proportional to population, as well as the percent of Minority population (middle) at census block centroid. The size of census block is proportional to percent Minority.

####
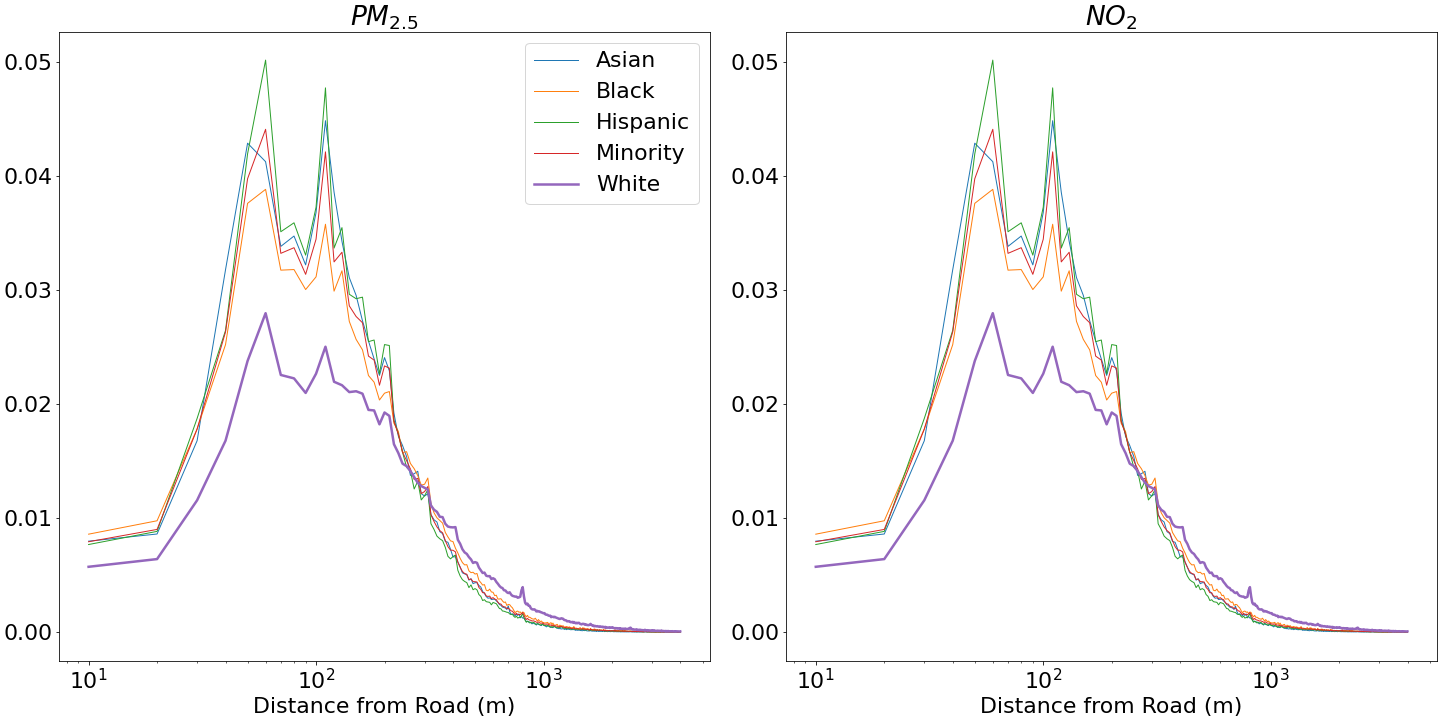
S18 Fig. Proportion of Minority and White populations as a function of distance from road aggregated at 10m from major roads across the continental U.S.

####
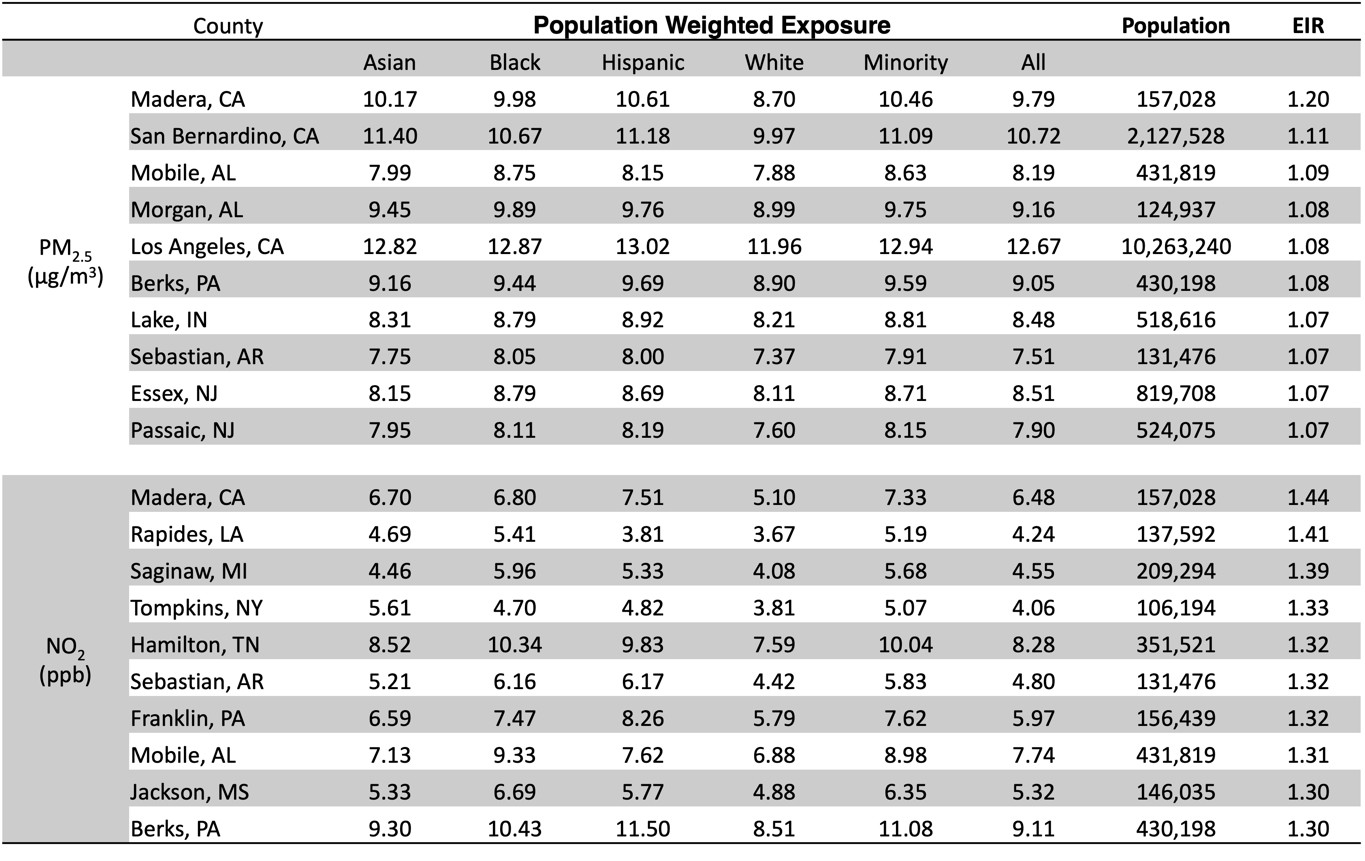
Table S5. Top 10 counties with highest exposure inequity ratio (EIR) with population greater than 100,000.

# References

1. Bates JT, Pennington AF, Zhai X, Friberg MD, Metcalf F, Darrow L, et al. Application and evaluation of two model fusion approaches to obtain ambient air pollutant concentrations at a fine spatial resolution (250m) in Atlanta. Environmental Modelling and Software. 2018;109: 182–190. doi:10.1016/j.envsoft.2018.06.008

2. Lefebvre W, Vercauteren J, Schrooten L, Janssen S, Degraeuwe B, Maenhaut W, et al. Validation of the MIMOSA-AURORA-IFDM model chain for policy support: Modeling concentrations of elemental carbon in Flanders. Atmos Environ. 2011;45: 6705–6713. doi:10.1016/j.atmosenv.2011.08.033

3. Oh I, Hwang MK, Bang JH, Yang W, Kim S, Lee K, et al. Comparison of different hybrid modeling methods to estimate intraurban NO2 concentrations. Atmos Environ. 2021;244: 117907. doi:10.1016/j.atmosenv.2020.117907

4. Scheffe RD, Strum M, Phillips SB, Thurman J, Eyth A, Fudge S, et al. Hybrid modeling approach to estimate exposures of hazardous air pollutants (HAPs) for the National air Toxics Assessment (NATA). Environ Sci Technol. 2016;50: 12356–12364. doi:10.1021/acs.est.6b04752

5. Chang SY, Vizuete W, Serre M, Vennam LP, Omary M, Isakov V, et al. Finely Resolved On-Road PM2.5and Estimated Premature Mortality in Central North Carolina. Risk Analysis. 2017;37: 2420–2434. doi:10.1111/risa.12775

6. Zhai X, Russell AG, Sampath P, Mulholland JA, Kim BU, Kim Y, et al. Calibrating R-LINE model results with observational data to develop annual mobile source air pollutant fields at fine spatial resolution: Application in Atlanta. Atmos Environ. 2016;147: 446–457. doi:10.1016/j.atmosenv.2016.10.015

7. US EPA. National Emissions Inventory (NEI). 2021 [cited 12 Aug 2021]. Available: https://www.epa.gov/air-emissions-inventories/national-emissions-inventory-nei

8. Punger EM, West JJ. The effect of grid resolution on estimates of the burden of ozone and fine particulate matter on premature mortality in the USA. Air Qual Atmos Health. 2013;6: 563–573. doi:10.1007/s11869-013-0197-8
