# Supplementary figures and images for: A hyperlocal hybrid data fusion near-road PM2.5 and NO2 annual risk and environmental justice assessment across the United States
Source: PLoS One. 2023 Jun 1;18(6):e0286406. doi: 10.1371/journal.pone.0286406 (PMC10234552; doi:10.1371/journal.pone.0286406)

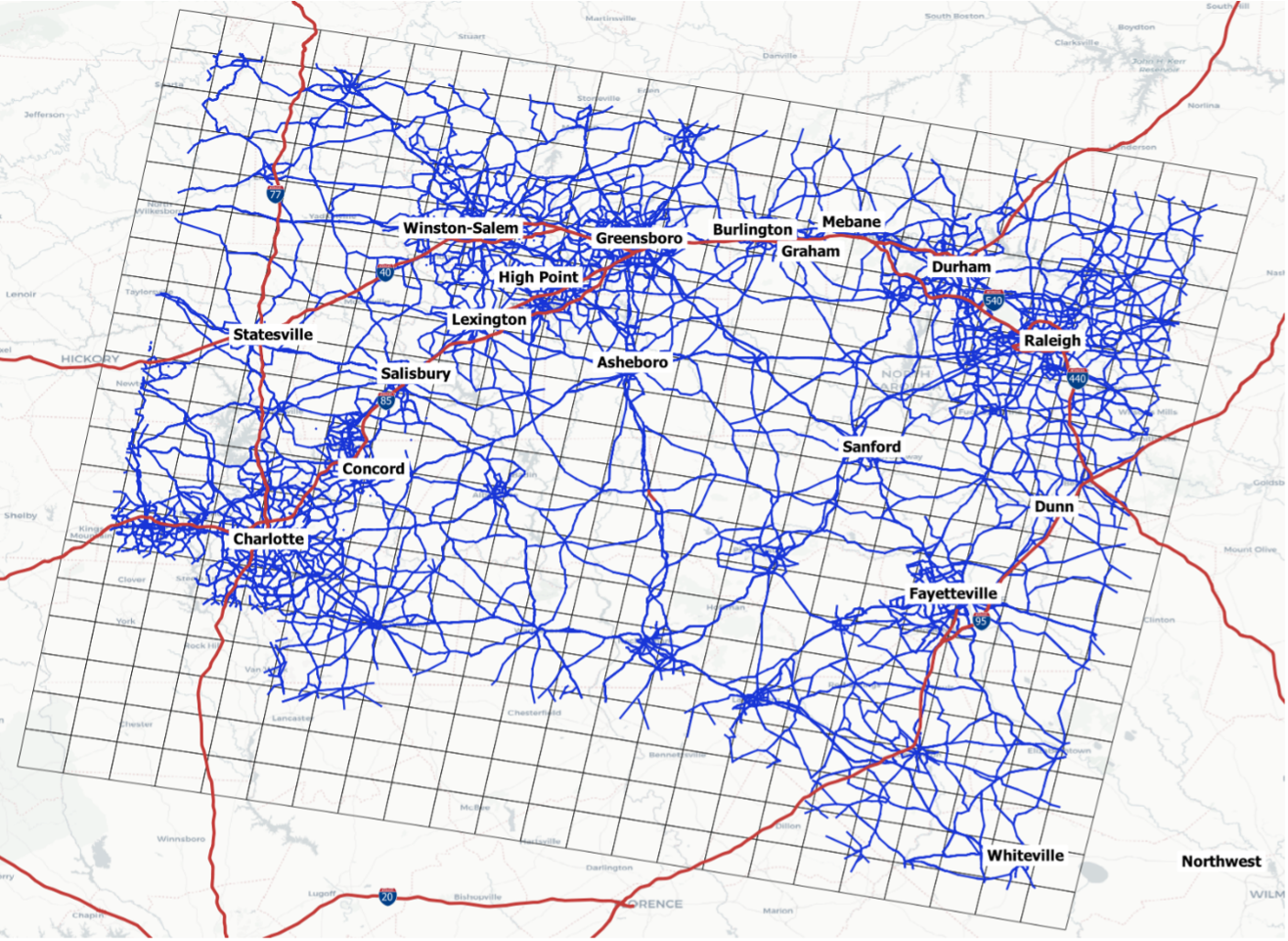

Supplement: S2 File — (ZIP) [file pone.0286406.s002.zip › SI_Figs/S1_Fig.png]

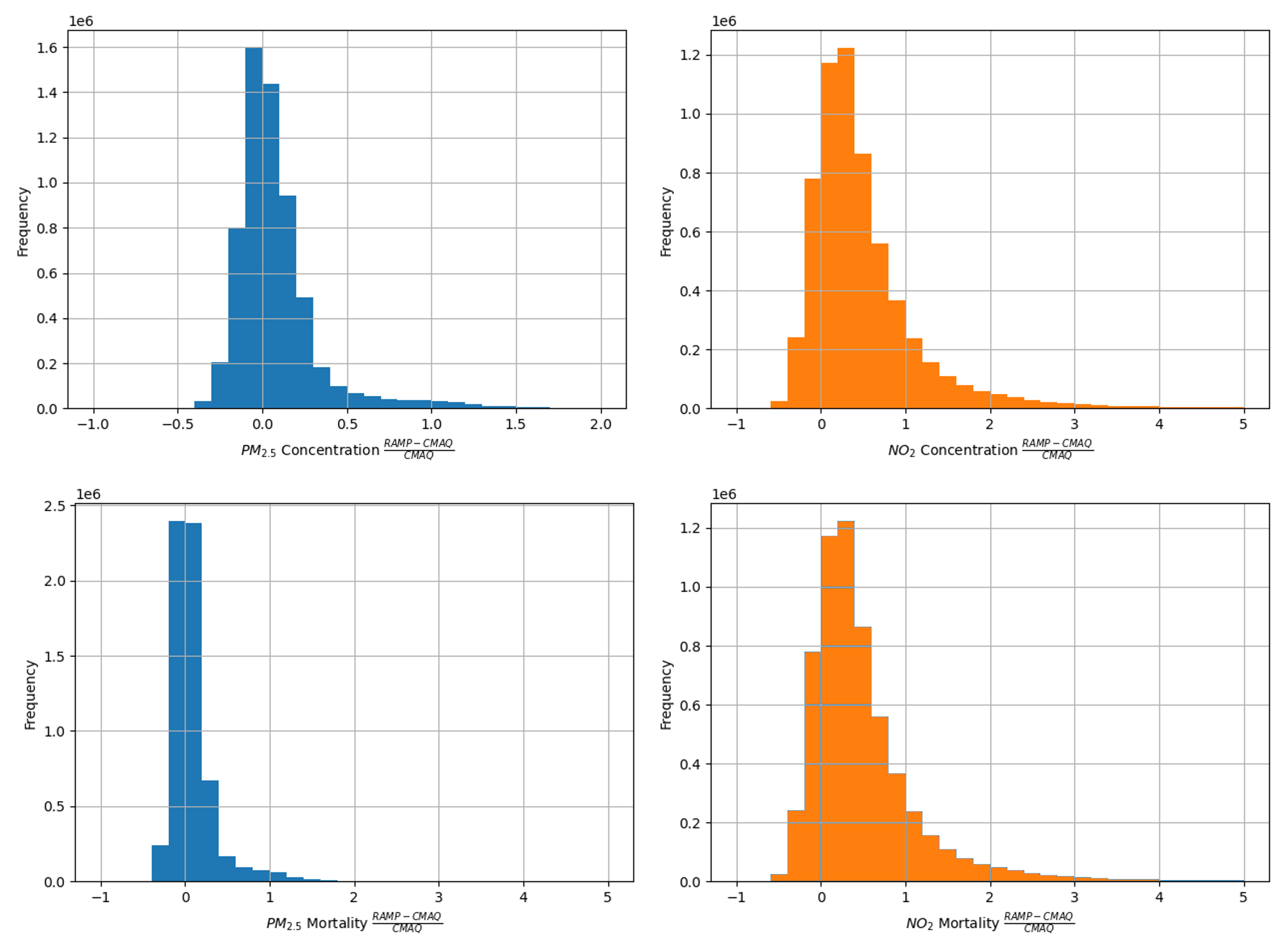

Supplement: S2 File — (ZIP) [file pone.0286406.s002.zip › SI_Figs/S10_Fig.png]

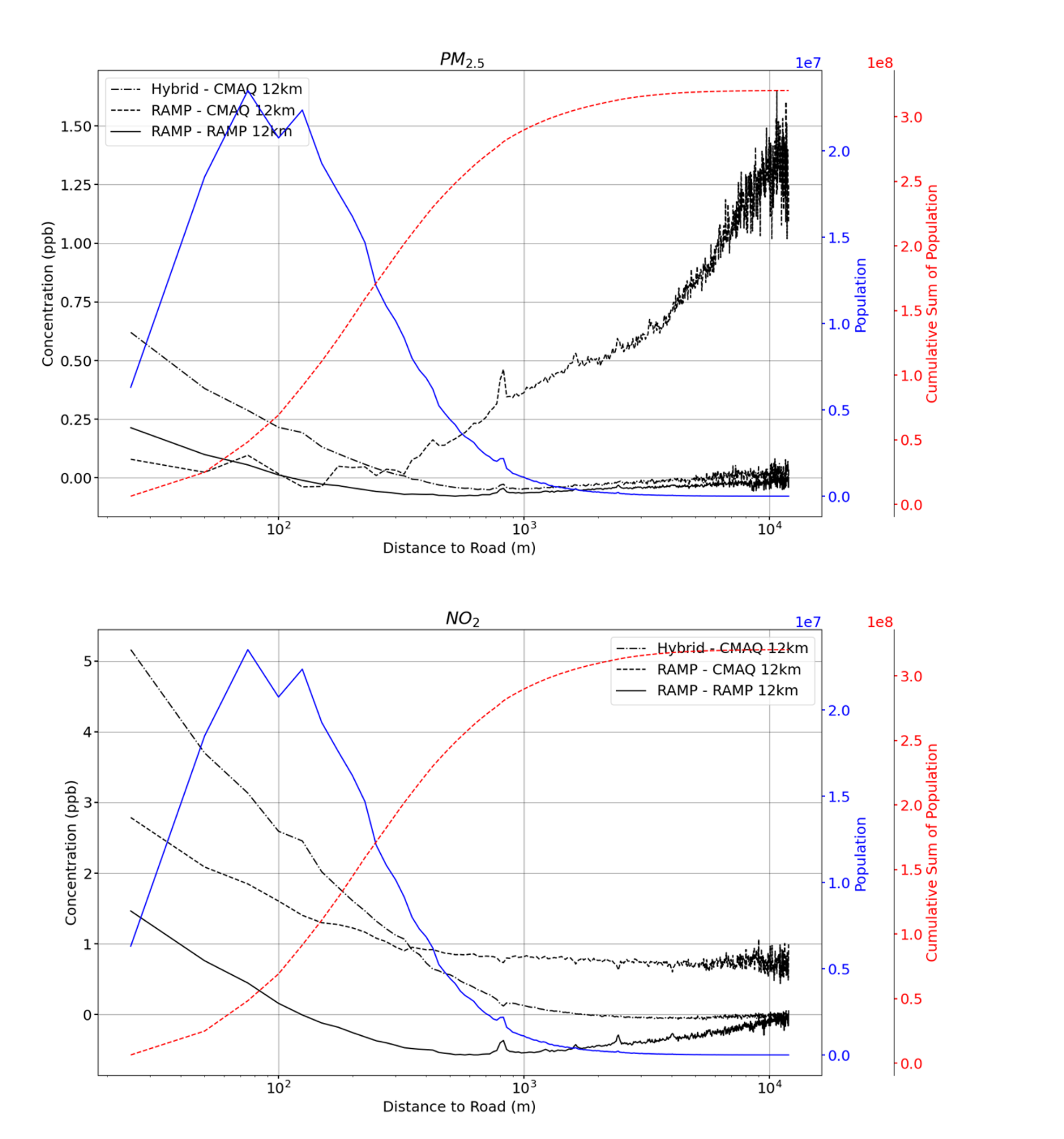

Supplement: S2 File — (ZIP) [file pone.0286406.s002.zip › SI_Figs/S11_Fig.png]

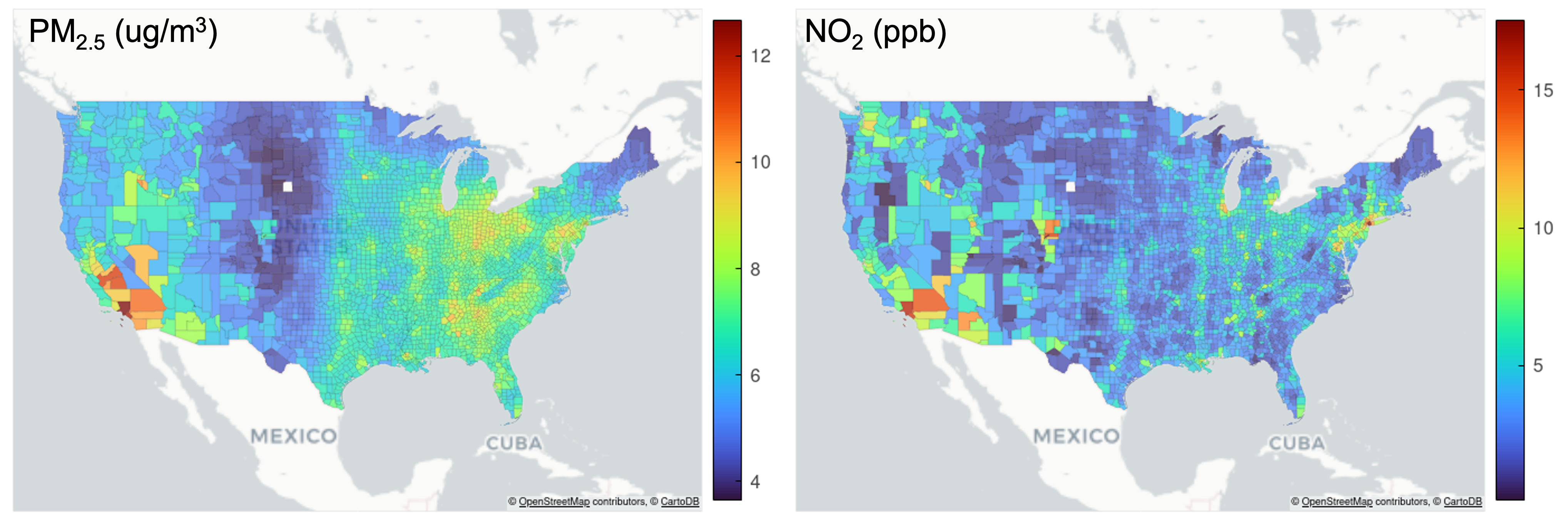

Supplement: S2 File — (ZIP) [file pone.0286406.s002.zip › SI_Figs/S12_Fig.png]

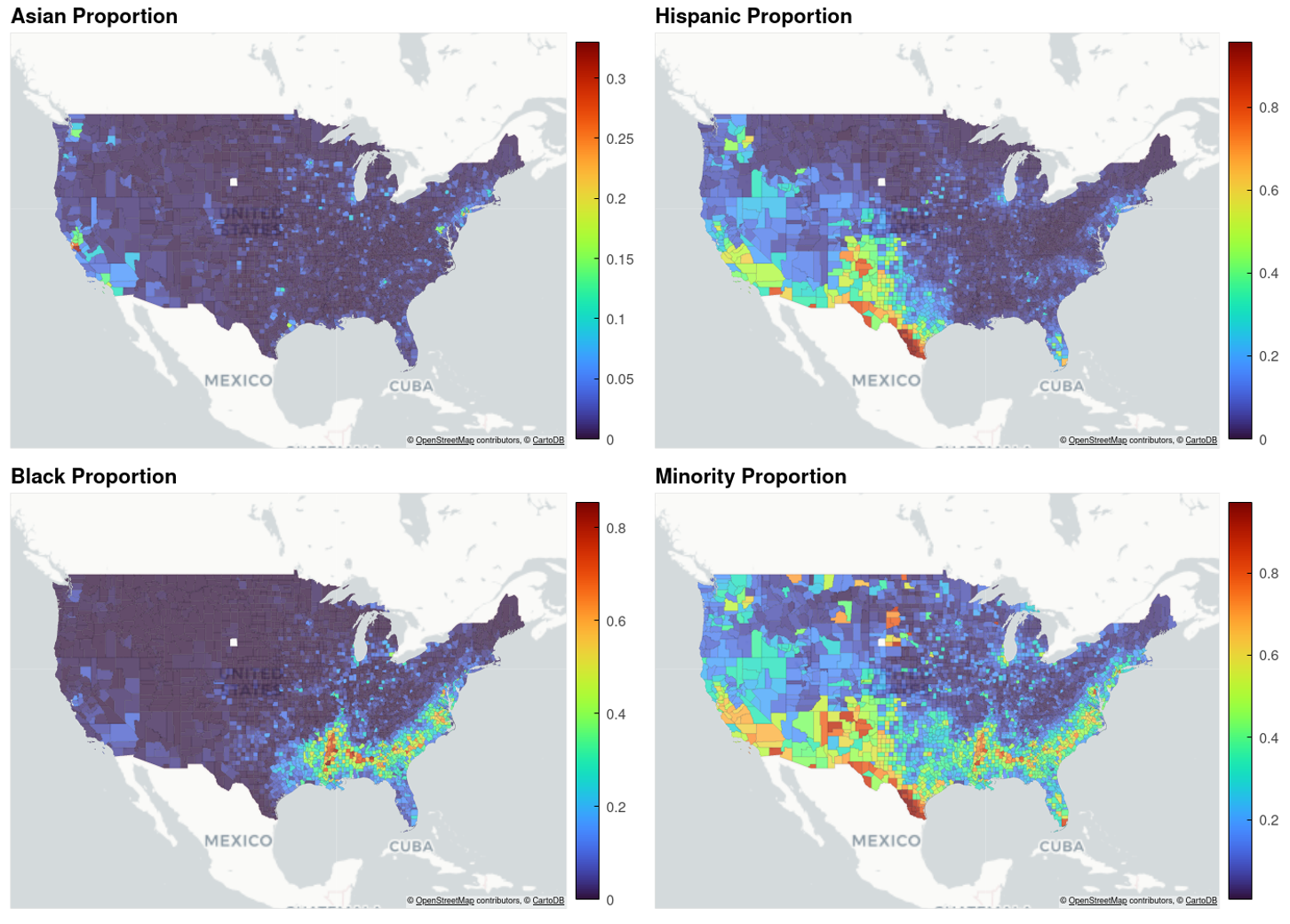

Supplement: S2 File — (ZIP) [file pone.0286406.s002.zip › SI_Figs/S13_Fig.png]

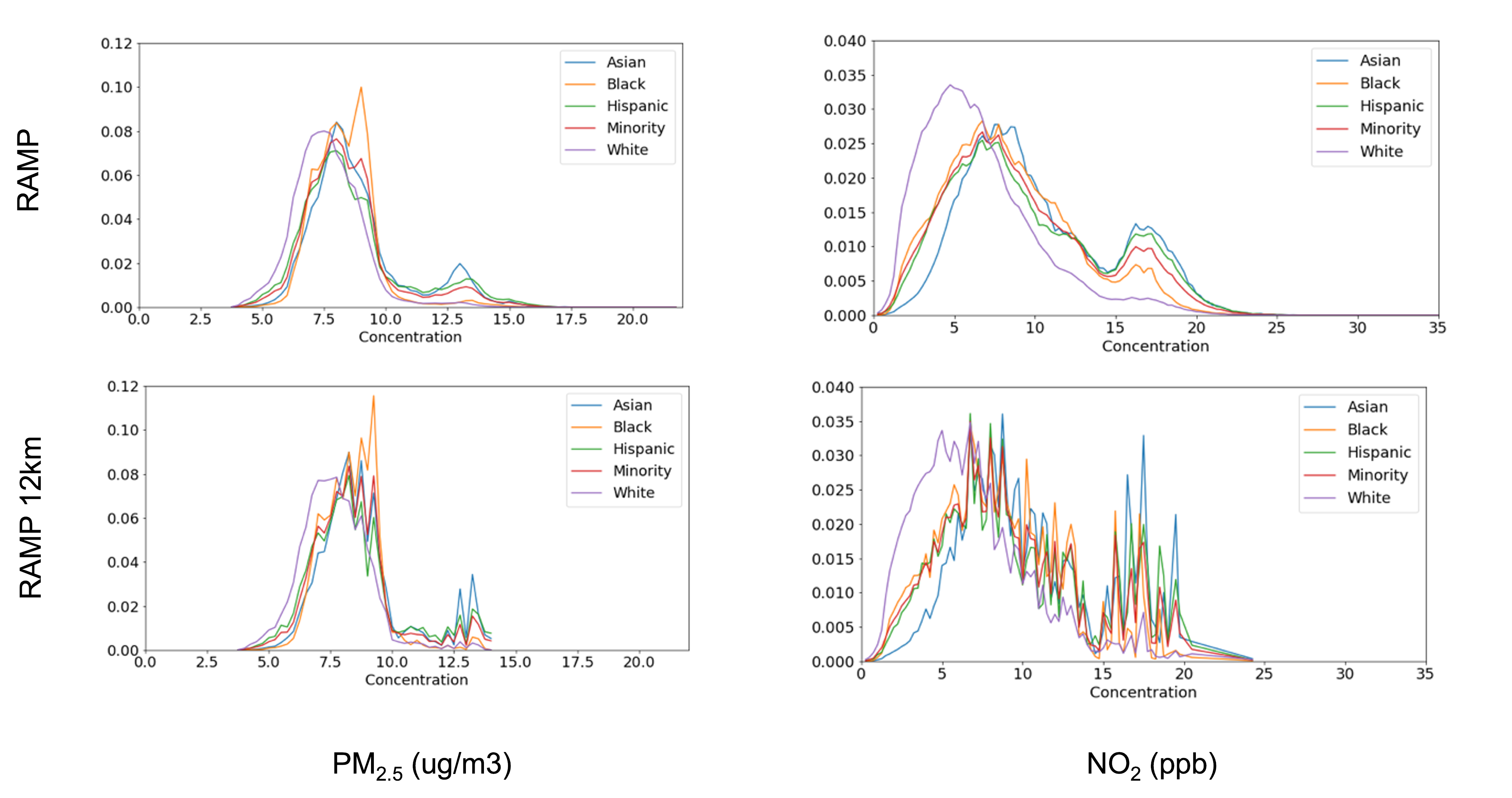

Supplement: S2 File — (ZIP) [file pone.0286406.s002.zip › SI_Figs/S14_Fig.png]

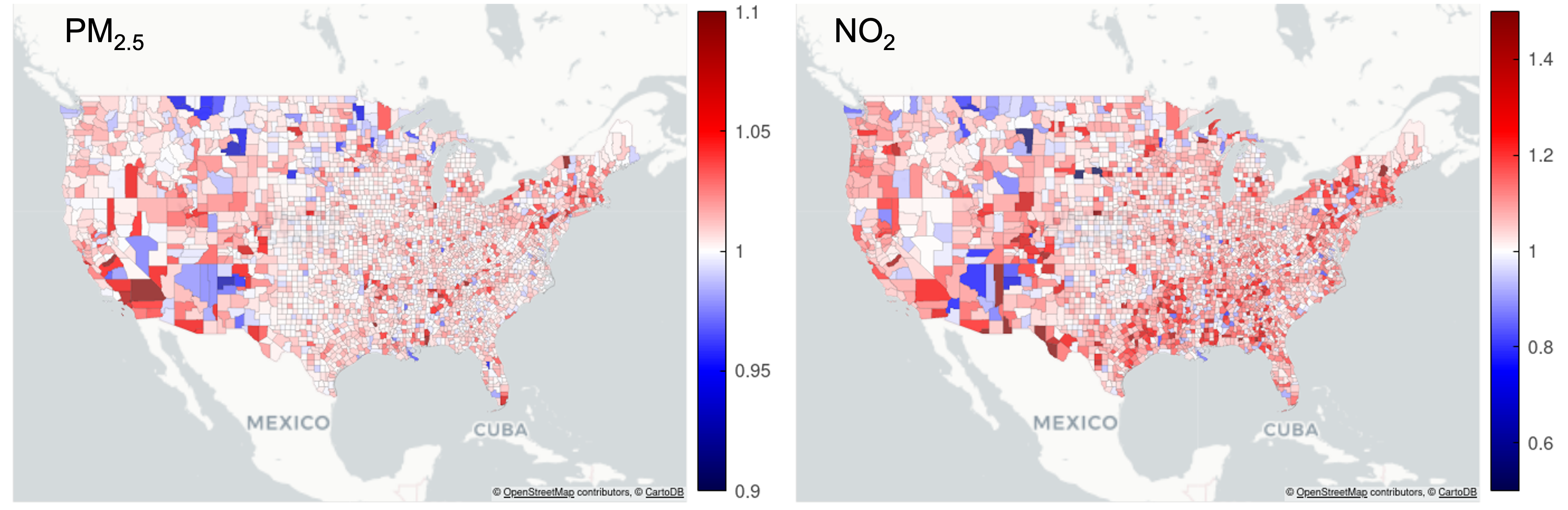

Supplement: S2 File — (ZIP) [file pone.0286406.s002.zip › SI_Figs/S15_Fig.png]

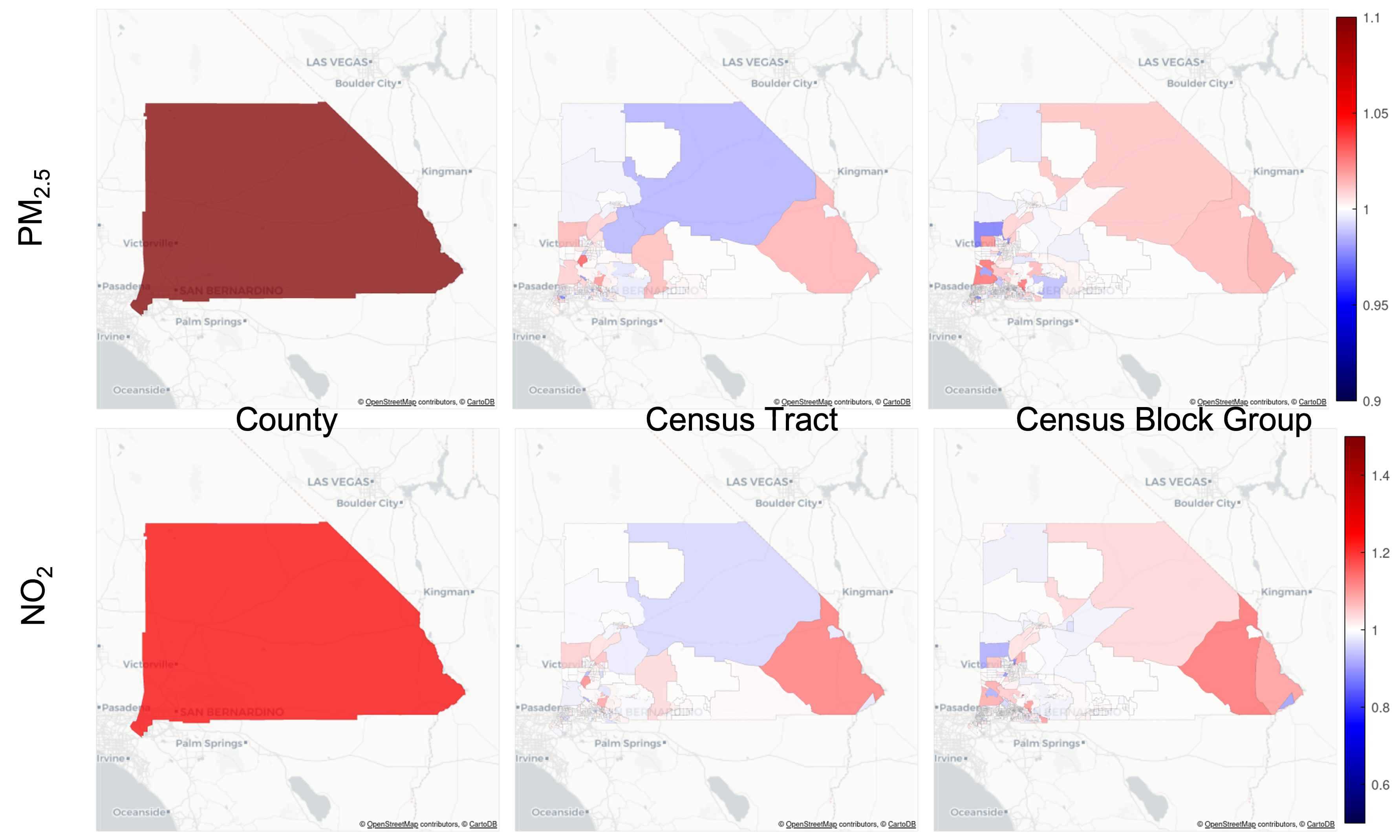

Supplement: S2 File — (ZIP) [file pone.0286406.s002.zip › SI_Figs/S16_Fig.png]

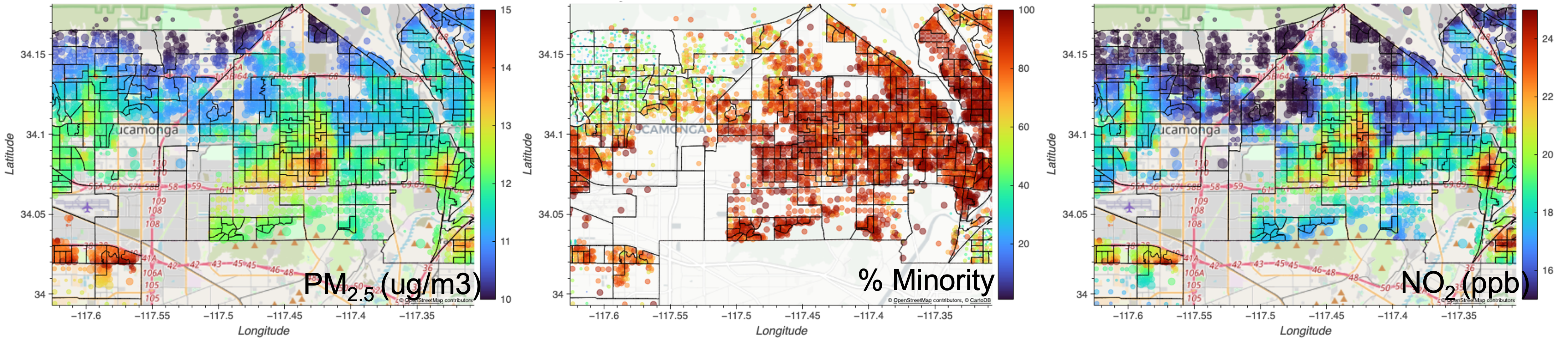

Supplement: S2 File — (ZIP) [file pone.0286406.s002.zip › SI_Figs/S17_Fig.png]

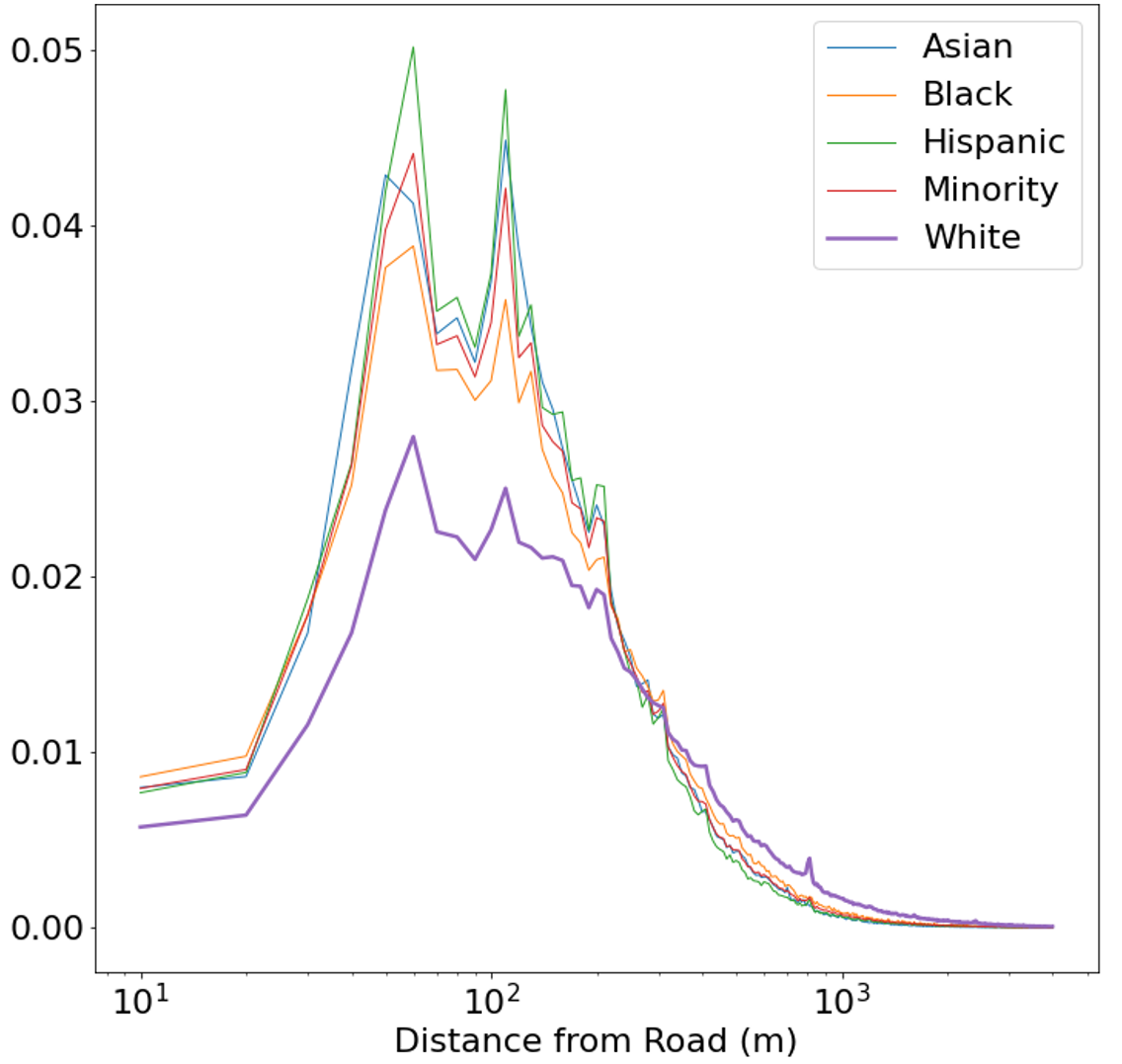

Supplement: S2 File — (ZIP) [file pone.0286406.s002.zip › SI_Figs/S18_Fig.png]

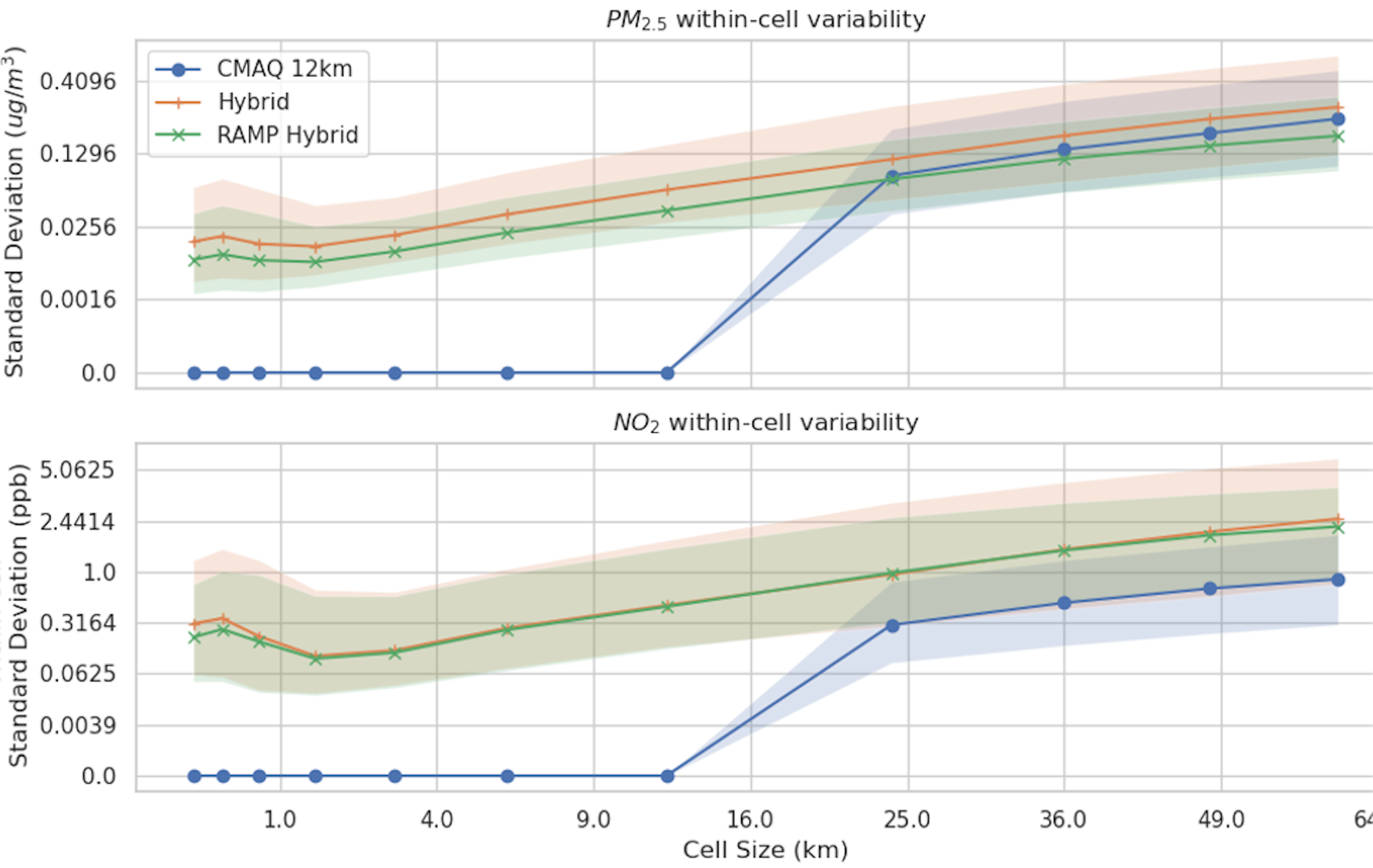

Supplement: S2 File — (ZIP) [file pone.0286406.s002.zip › SI_Figs/S2_Fig.png]

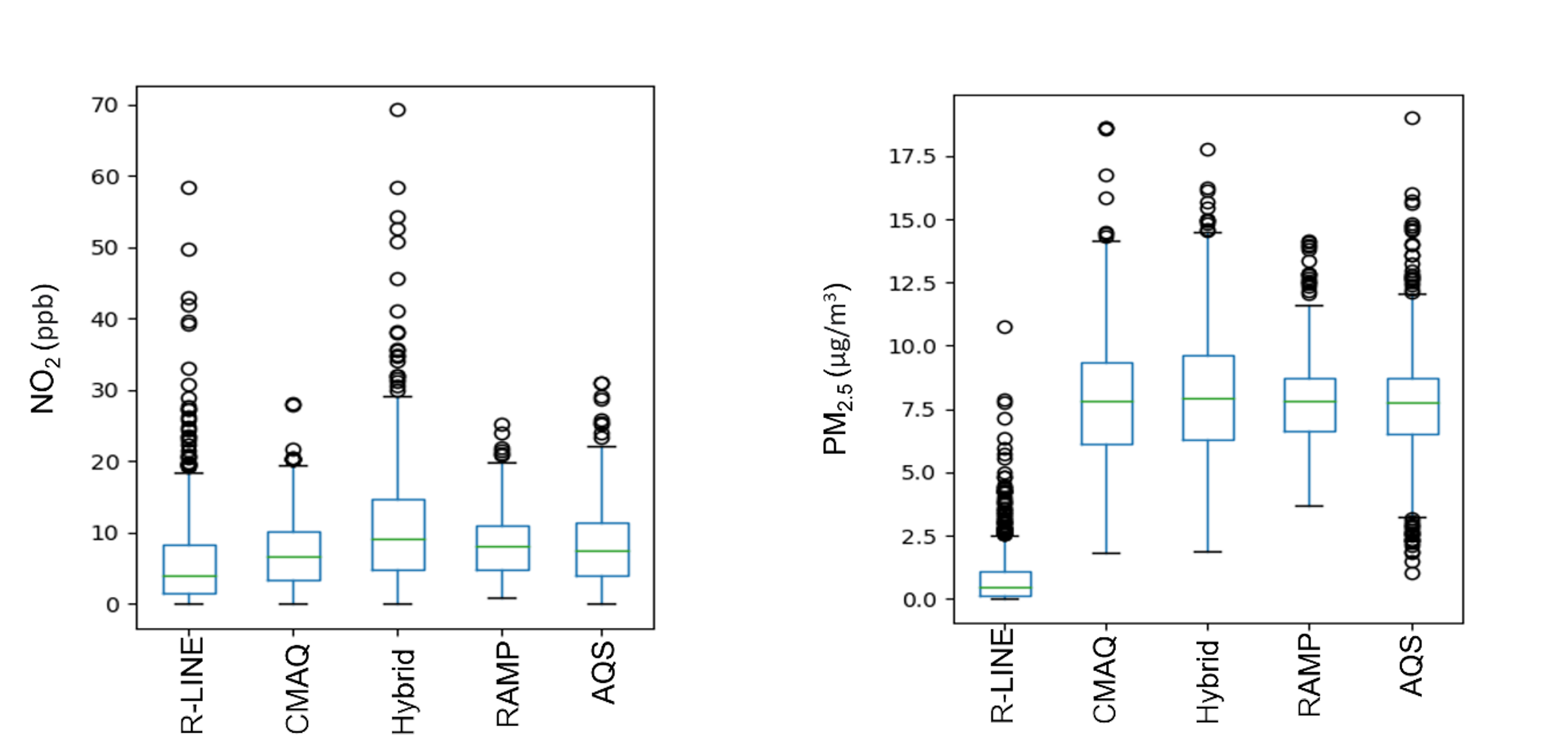

Supplement: S2 File — (ZIP) [file pone.0286406.s002.zip › SI_Figs/S3_Fig.png]

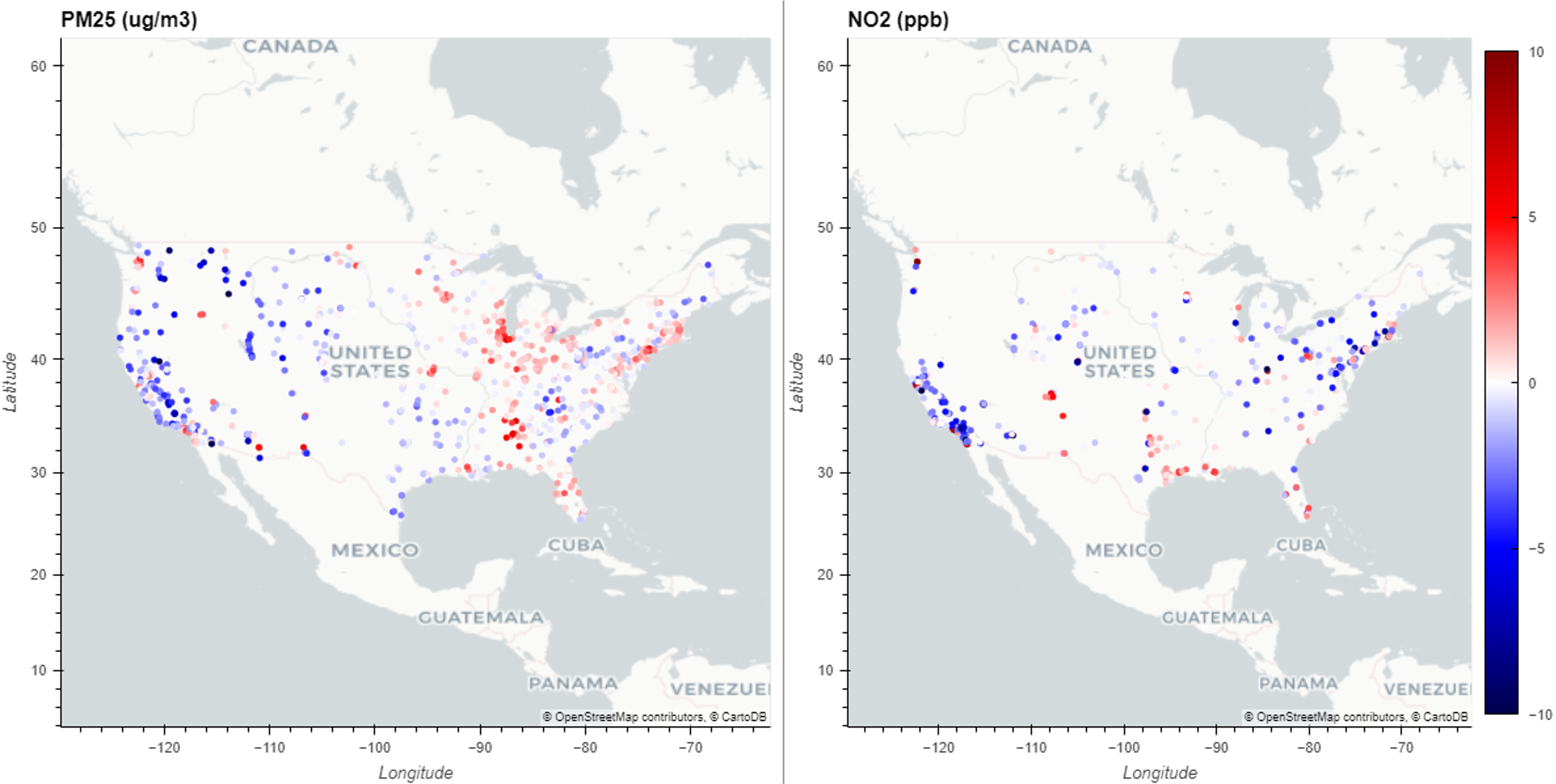

Supplement: S2 File — (ZIP) [file pone.0286406.s002.zip › SI_Figs/S4_Fig.png]

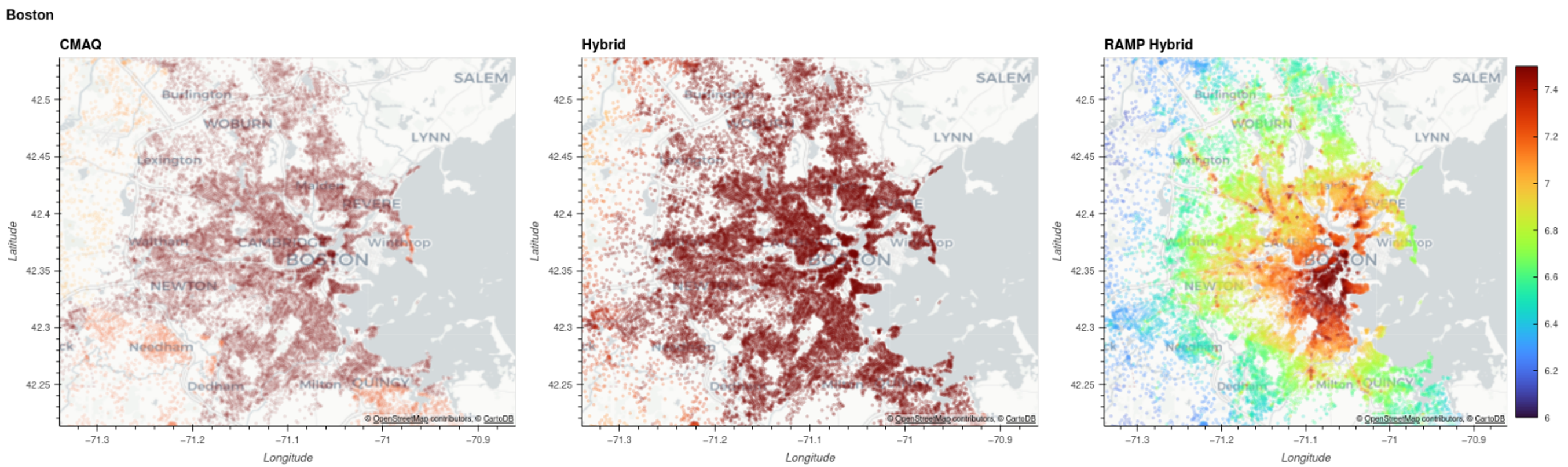

Supplement: S2 File — (ZIP) [file pone.0286406.s002.zip › SI_Figs/S5_Fig.png]

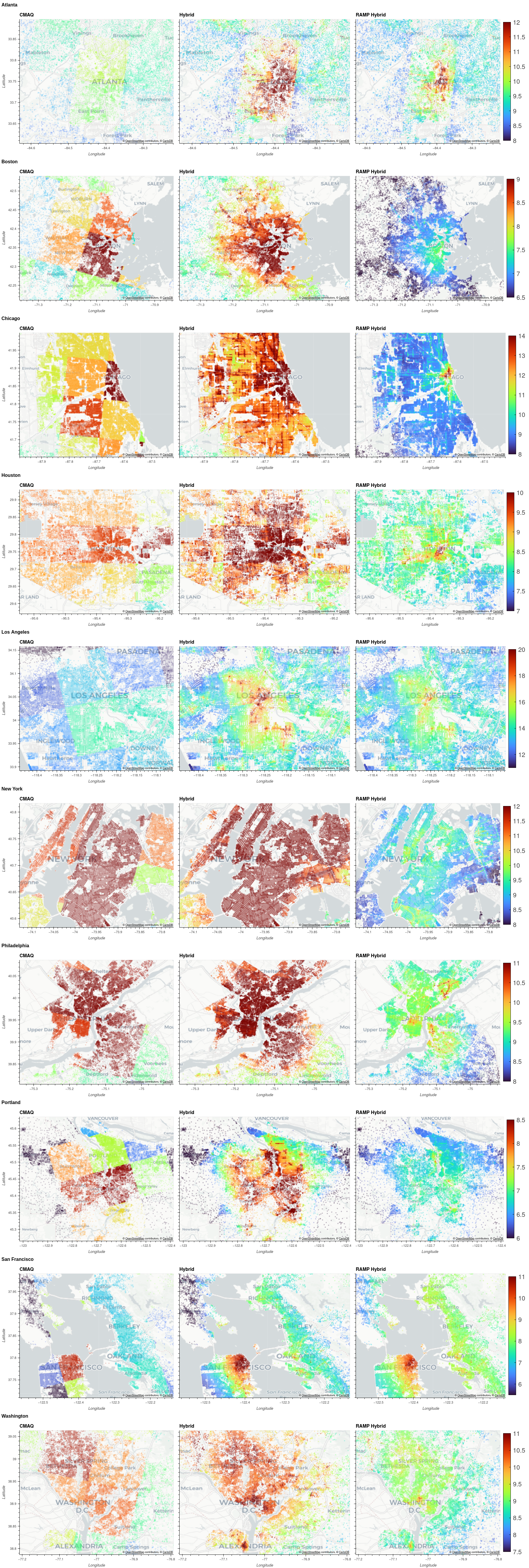

Supplement: S2 File — (ZIP) [file pone.0286406.s002.zip › SI_Figs/S6_Fig.png]

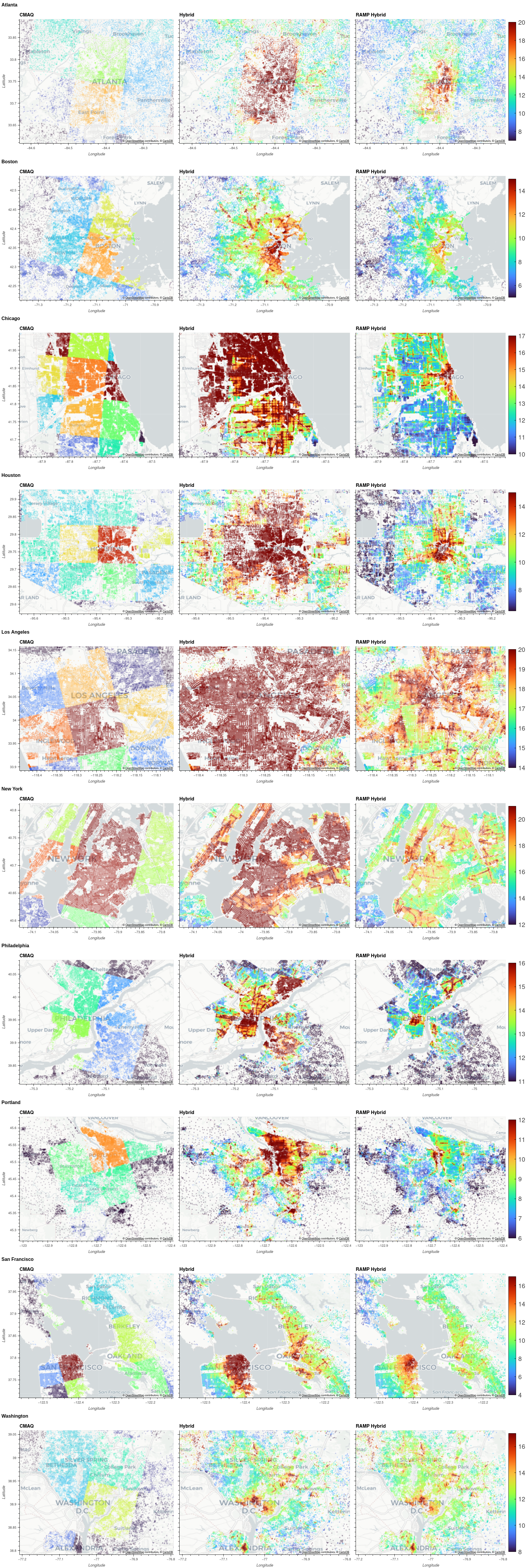

Supplement: S2 File — (ZIP) [file pone.0286406.s002.zip › SI_Figs/S7_Fig.png]

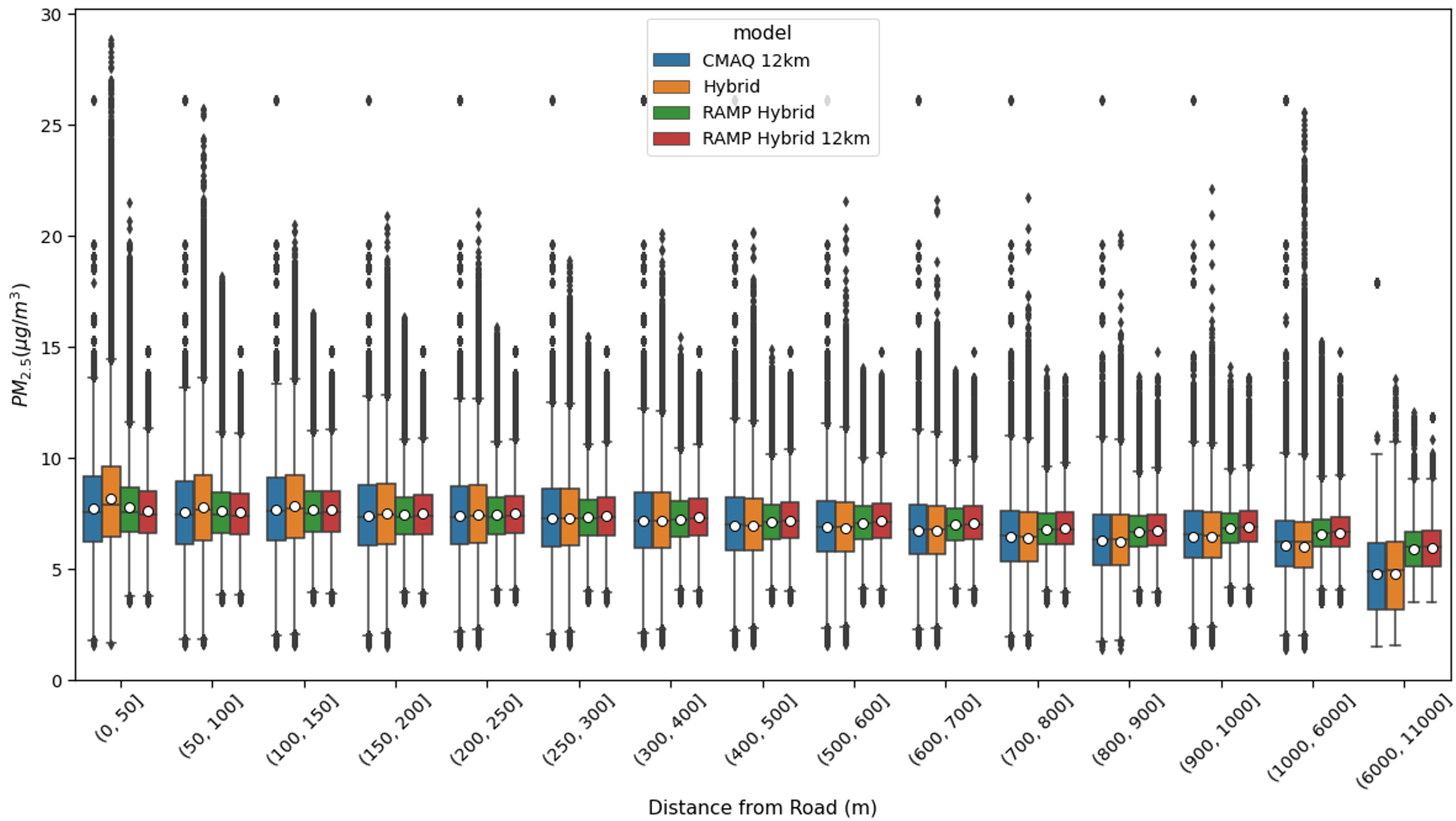

Supplement: S2 File — (ZIP) [file pone.0286406.s002.zip › SI_Figs/S8_Fig.png]

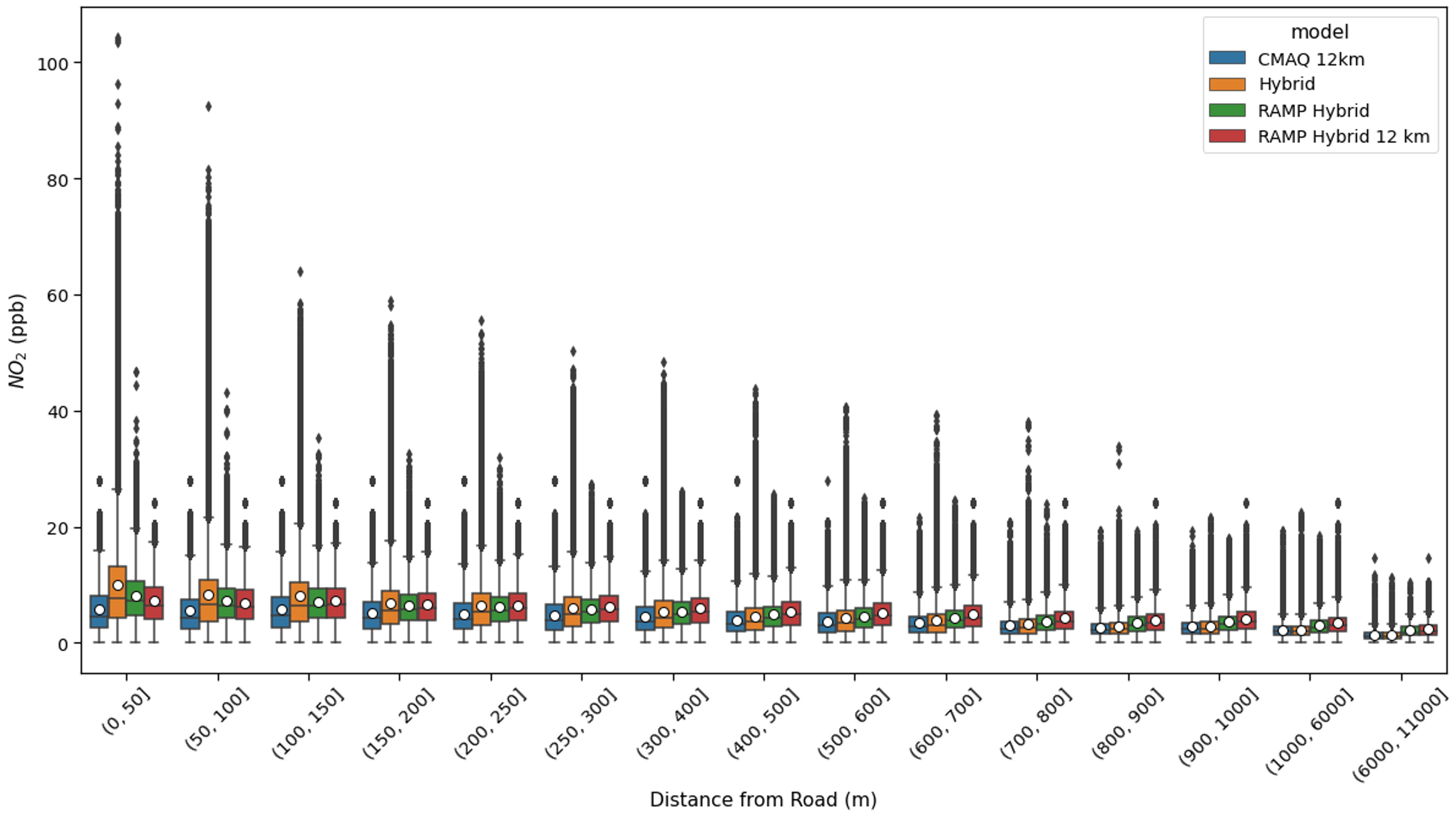

Supplement: S2 File — (ZIP) [file pone.0286406.s002.zip › SI_Figs/S9_Fig.png]
